# Comparative accuracy of cervical cancer screening strategies in healthy asymptomatic women: a systematic review and network meta-analysis

**DOI:** 10.1038/s41598-021-04201-y

**Published:** 2022-01-07

**Authors:** Teruhiko Terasawa, Satoyo Hosono, Seiju Sasaki, Keika Hoshi, Yuri Hamashima, Takafumi Katayama, Chisato Hamashima

**Affiliations:** 1 Section of General Internal Medicine, Department of Emergency and General Internal Medicine, Fujita Health University School of Medicine, 1-98 Dengakugakubo, Kutsukakecho, Toyoake, Aichi 470-1192 Japan; 2 Division of Cancer Screening Assessment and Management, Center for Public Health Science, National Cancer Center, Tokyo, Japan; 3 Center for Preventive Medicine, St. Luke’s International Hospital Affiliated Clinic, Tokyo, Japan; 4 Center for Public Health Informatics, National Institute of Public Health, Wako, Japan; 5 Department of Population Health Science, Bristol Medical School, University of Bristol, Bristol, UK; 6 Department of Statistics and Computer Science, College of Nursing Art and Science, University of Hyogo, Hyogo, Japan; 7 Department of Nursing, Faculty of Medical Technology, Teikyo University, Tokyo, Japan

**Keywords:** Cancer screening, Cervical cancer, Health care

## Abstract

To compare all available accuracy data on screening strategies for identifying cervical intraepithelial neoplasia grade ≥ 2 in healthy asymptomatic women, we performed a systematic review and network meta-analysis. MEDLINE and EMBASE were searched up to October 2020 for paired-design studies of cytology and testing for high-risk genotypes of human papillomavirus (hrHPV). The methods used included a duplicate assessment of eligibility, double extraction of quantitative data, validity assessment, random-effects network meta-analysis of test accuracy, and GRADE rating. Twenty-seven prospective studies (185,269 subjects) were included. The combination of cytology (atypical squamous cells of undetermined significance or higher grades) and hrHPV testing (excepting genotyping for HPV 16 or 18 [HPV16/18]) with the either-positive criterion (OR rule) was the most sensitive/least specific, whereas the same combination with the both-positive criterion (AND rule) was the most specific/least sensitive. Compared with standalone cytology, non-HPV16/18 hrHPV assays were more sensitive/less specific. Two algorithms proposed for primary cytological testing or primary hrHPV testing were ranked in the middle as more sensitive/less specific than standalone cytology and the AND rule combinations but more specific/less sensitive than standalone hrHPV testing and the OR rule combination. Further research is needed to assess these results in population-relevant outcomes at the program level.

## Introduction

Cervical cancer is the fourth most frequently diagnosed cancer and fourth most common cause of cancer-specific mortality in women, with a worldwide estimated prevalence of 570,000 cases and 311,000 associated deaths in 2018^1,2^. Observational studies have clearly demonstrated a reduction in the invasive cancer incidence and mortality in well-organized screening programs using cervical cytological testing that have been implemented^3^. Moreover, randomized controlled trials (RCTs) of well-screened populations have shown that strategies incorporating testing for high-risk human papillomavirus (hrHPV) subtypes, which are the central etiological agents of cervical cancer pathogenesis^4^, were, in aggregate, associated with a reduction in the invasive cancer incidence relative to that shown by cytological screening alone^5^. Therefore, current guidelines recommend three primary screening options: cytological testing alone, standalone hrHPV testing, and cytological + hrHPV combination testing (co-testing)^6–10^. However, subsequent management strategies for women with positive primary testing are complex. Although specific triage and/or follow-up testing algorithms for primary cytology and co-testing^11^ and for primary hrHPV testing^9^ have been proposed, the evidence base to improve patient-important outcomes with these algorithms is immature.

The comparative effectiveness of alternative screening strategies should be based on a comprehensive assessment of benefits and harms. Given the low incidence and mortality due to cervical cancer in high-income countries and the challenges associated with conducting de novo large and long-term RCTs, decision modeling is an alternative realistic option to better understand the theoretical utility of the screening options^12^. In this regard, comprehensive synthesis of the screening accuracy, a key model parameter of cytological and hrHPV testing and their available combination algorithms reported in rigorously conducted paired-design studies, is a valuable intermediate step. However, recent meta-analyses have focused on either standalone cytological and/or hrHPV testing^13–15^ or a comparison of cytological testing with a specific combination algorithm not proposed in guidelines only^16^.

For those studies that assessed the diagnostic accuracy of selected and different pairs of tests of interest and their combination algorithms, network meta-analysis of diagnostic test accuracy studies is a useful approach that can compare all the assessed tests and combination algorithms in a single analysis^17^. The current study aimed to perform network meta-analysis to quantitatively compare and rank the cross-sectional accuracy of all reported screening algorithms based on cytological and hrHPV testing. We specifically focused on the comparative accuracy of guideline-proposed combination algorithms by examining data derived from primary studies of healthy asymptomatic women that addressed verification bias because such bias is commonly observed in cancer screening accuracy studies.

## Methods

This extended systematic review is based on an update evidence review conducted for revision of the Japanese Guidelines for Cervical Cancer Screening^18,19^. Although the complete evidence review was planned before analysis, no protocol was registered for this extended review. This report followed PRISMA guidelines for diagnostic test accuracy (PRISMA-DTA)^20^ and did not require ethics review or patient consent.

### Search strategy

We searched OVID MEDLINE and EMBASE for publications between January 1, 1992, and October 14, 2020, with no language restrictions. The search strategies are detailed in the Supplementary methods. Complementarily, the reference lists of eligible studies and relevant review articles were also screened for other appropriate studies.

### Study eligibility

Three paired reviewers independently double screened the first 3000 abstracts in a calibration phase. The same reviewers single screened the remaining abstracts. Two reviewers independently determined the eligibility of potential full-text articles, with discrepancies adjudicated by a third reviewer. Only fully paired-design screening studies of cytology and hrHPV testing, either opportunistic or organized screening, aimed at detecting cervical intraepithelial neoplasia ≥ grade 2 (CIN2+) in healthy asymptomatic women were eligible for inclusion. We included all studies that performed either routine colposcopy-directed biopsy or colposcopy and selective biopsy in all screened women to verify target lesions along with studies that performed either of the colposcopy methods among women with protocol-specified screening results and statistical corrections for data from unverified samples. In studies that analyzed both eligible and ineligible populations, only those with relevant and extractable data were included. In case of multiple publications, we included the publication with the largest sample size (see Supplementary methods for more details)**.**

### Data extraction

One reviewer extracted descriptive data, which were independently confirmed by another reviewer. Next, two reviewers independently extracted numerical data, with discrepancies resolved by consensus. We preferred cross-tabulated count data over reported accuracy estimates when both data types were extractable (see Supplementary methods for more details).

### Operationalization

Cytology results were standardized according to the Bethesda system^21,22^ if other classification systems had been used. For studies that used both conventional and liquid-based cytology tests (CC and LBC, respectively), we favored LBC data over CC data; we jointly analyzed both smear preparation methods.

Operationally, hrHPV assays were categorized into four groups: hybridization with signal amplifications of DNA (e.g., Hybrid Capture 2 [HC2], Qiagen, Gaithersburg, MD), polymerase chain reaction (PCR) of DNA from ≥ 13 hrHPV genotypes, amplification of E6/E7 viral messenger RNA (mRNA), and assays identifying DNA or RNA of genotypes, either HPV16 or HPV18 or both (HPV16/18)^23^. For mRNA-based genotyping, since the genotype HPV45 was additionally targeted with HPV16 and HPV18 (HPV16/18/45), we adopted these results. HC2 positivity was defined as ≥ 1.0 relative light units. We did not assess point-of-care testing platforms (e.g., careHPV, Qiagen, Gaithersburg, MD).

We operationally categorized combination tests as follows: (i) combination algorithms based on the OR rule (women with either test positive were categorized as screening positive while women with both tests negative as screening negative) or the AND rule (women with both tests positive were categorized as screening positive while women with at least 1 negative test as negative)^24^; (ii) thresholds for cytological testing as, e.g., undetermined significance or worse grades (≥ ASCUS), or low- or high-grade squamous intraepithelial lesions or worse grades (≥ LSIL or ≥ HSIL, respectively); and (iii) hrHPV assays (Table 1)^6–9,25^. As cross-sectional representation of guidelines-proposed algorithms, we assessed two specific strategies: “ ≥ LSIL OR [hrHPV AND ASCUS]”, which classified only women with cytologic testing ≥ LSIL, or both by cytologic testing ASCUS and hrHPV testing positive as screening positive; and “HPV16/18(/45) OR [hrHPV AND ≥ ASCUS]”, which classified only women with HPV genotypes 16 or 18 (or 45) positive, or both cytologic testing ≥ ASCUS and hrHPV testing positive for non-16/18(/45) hrHPV genotypes as screening positive (Table 1).

**Table 1:** Operational categorizations of cytological testing, assays for hrHPV testing, and their combination algorithms.

	Methods	FDA-approved systems
**Standalone or component tests**		
Cytology		
Conventional or liquid-based cytology	A specified cytological grade, ASCUS, ASCH, LSIL, or HSIL (and higher grades if indicated) defined by the Bethesda system is used as the positive criterion	SurePath (TriPath Imaging, Inc., Burlington, NC) and ThinPrep (Cytyc Corp, Marlborough, MA)
hrHPV assays		
Signal amplifications	Nucleic acid hybridization with signal amplification or cleavage-based signal amplification of ≥ 13 hrHPV DNA	Hybrid Capture 2 (Qiagen, Gaithersburg, MD); Cervista HPV HR (Hologic, Madison, WI)
PCR-based tests	PCR of DNA from ≥ 13 hrHPV genotypes	Cobas HPV test (Roche Molecular Systems, Pleasanton, CA)
mRNA-based tests	Amplification of E6/E7 viral mRNA	Aptima (Hologic, Bedford, MA)
HPV 16/18(/45)	Genotyping assays identifying DNA or RNA of HPV genotypes either 16 or 18 or both	Cobas HPV test (Roche Molecular Systems, Pleasanton, CA); Aptima HPV16 and HPV18/45 (Hologic, Bedford, MA); Cervista HPV 16/18 (Hologic, Madison, WI)

Specific algorithms	Test operation system	Definition of screening strategy positive
**Combination tests**		
AND rule (e.g., hrHPV AND ≥ ASCUS)	Co-testing or serial testing	Women with both positive tests as screening positive and women with ≥ 1 negative test as screening negative
OR rule (e.g., hrHPV OR ≥ ASCUS)	Co-testing only	Women with ≥ 1 positive test as screening positive and women with both negative tests as screening negative
≥ LSIL OR [hrHPV AND ASCUS]	Co-testing or primary cytologic testing with reflex to hrHPV testing by a joint guideline published in 2012 among ACS, ASCCP, and ASCP	Cytologic testing ≥ LSIL, or both by cytologic testing ASCUS and hrHPV testing positive
HPV16/18(/45) OR [hrHPV AND ≥ ASCUS]	Co-testing or primary hrHPV testing with HPV genotyping with reflex to cytologic testing proposed by the SGO 2015	HPV genotypes 16 or 18 positive, or both cytologic testing ≥ ASCUS and hrHPV testing positive for non-16/18 hrHPV genotypes

*ACS* American Cancer Society, *ASCCP* American Society for Colposcopy and Cervical Pathology, *ASCH* atypical squamous cells, cannot exclude HSIL, *ASCP* American Society for Clinical Pathology, *ASCUS* atypical squamous cells of undetermined significance, *BPR* both-positive rule, *DNA* deoxyribonucleic acid, *EPR* either-positive rule, *hrHPV* high-risk human papillomavirus, *HPV* human papillomavirus, *HR* high risk, *HSIL* high-grade squamous intraepithelial lesion, *LSIL* low-grade squamous intraepithelial lesion, *mRNA* messenger ribonucleic acid, *SGO* Society for Gynecologic Oncology.

### Quality assessment

Paired independent reviewers double rated the validity of a study using a risk of bias tool for comparative diagnostic accuracy studies (QUADAS-C)^26^, an extension to the existing Quality Assessment of Diagnostic Accuracy Studies 2 tool^27^. Discrepancies were resolved via consensus. Operationally, a study was defined to have low risk of verification bias only when all screened samples had been histologically verified.

### Data synthesis and statistical analysis

The primary outcome was sensitivity and specificity for detecting CIN2+. We used their relative risk values for and absolute differences in (Δ) sensitivity and specificity for any paired alternative screening algorithms (e.g., a standalone test *vs.* a combination algorithm) as measures of comparative accuracy.

Between-study heterogeneity was assessed visually by using crosshair plots of sensitivity and specificity estimates in the receiver operating characteristic (ROC) space^28^. We calculated the average sensitivity and specificity estimates and their derived relative and Δ sensitivity and specificity values with their corresponding 95% credible intervals (CrIs) by using an arm-based, two-stage hierarchical, Bayesian bivariate random-effects network meta-analysis model^29^. Credible regions for the average estimates were constructed by using the standard method^30^. For comparison, we also calculated average sensitivity and specificity estimates separately by using the standard bivariate meta-analysis model for diagnostic accuracy^31^. Hierarchical summary ROC (HSROC) curves were derived on the basis of the estimated parameters^32^.

We performed study-level univariable meta-regression for the following prespecified binary predictors when ≥ 10 studies were available: study location (countries ranked as “very high human development” by the Human Development Index 2017^33^ *vs.* those that were not), study design (histology-based *vs.* colposcopy-based verification), and type of sample collectors (physicians *vs.* nonphysicians). Scarce data on young individuals (< 30 years old) precluded meta-regression based on age. Complete details of the methodology, model fitting, choice of prior distributions for parameters assessed, and operational definitions used in sensitivity analyses are provided in the Supplementary methods.

We used the Grading of Recommendation Assessment, Development, and Evaluation (GRADE) tool^34^ to assess the certainty of evidence and focused on the comparisons among cytological testing (≥ ASCUS) alone, standalone hrHPV assays, and the guideline-proposed combination algorithms. For calculating false negatives (FNs) and false positives (FPs), we assumed a healthy screening population of 1,000 women in which 20 are CIN2 + (i.e., a prevalence of 2%)^13^.

We did not evaluate funnel-plot asymmetry because the required tests did not permit valid assessment of the extent and impact of missing studies^20^. All analyses were performed by using WinBUGS 1.4.3 (MRC Biostatistics Unit, Cambridge, UK) and Stata/SE 16.1 (Stata Corp, College Station, TX)^35^. We estimated the probability that the true value (i.e., posterior distribution) of relative sensitivity or specificity was ≥ 1 (or ≤ 1) as a measure of superiority of a test over a comparator test. A conventional, frequentist, two-tailed *P-*value of 0.05 corresponds to a Bayesian posterior probability of 0.025, which we considered to be the threshold of statistical significance.

## Results

### Study selection

Our literature search identified 15,488 citations, of which 27 prospective studies reported in 35 publications corresponding to 185,269 women were included for the meta-analysis (Supplementary Fig. S1)^36–70^. Supplementary material provides a list of excluded studies.

### Characteristics of included studies

All included studies had a prospective design, and 14 studies (52%) were from high-income countries (Table 2). The average age of study participants ranged from 25 to 47 years. Data on type of sample collectors was available for 20 studies (74%), with physician collectors in 14 studies and nonphysician providers, typically trained nurses or midwives, in 6 studies. Thirteen studies had used only CC, and 12 had adopted only LBC, whereas two other studies had used both CC and LBC (Table 2). Of the four available hrHPV testing subgroups, HC2 was the most commonly reported hrHPV assay (assessed in 20 studies), whereas six studies assessed PCR-based tests, four genotyped for HPV16/18, and three used mRNA-based tests, of which also genotyped for HPV16/18/45. Data on one or more combination algorithm(s) were available in 19 studies (reported in 20 publications; 70%). The most commonly assessed combinations were HC2 AND ≥ ASCUS, which were reported in 10 studies. Reference standards were used for all participants with routine colposcopy-directed biopsy in three studies^36,39,40^ and colposcopy and selective biopsy in six studies (Table 2)^54,56,58–61^. Other studies performed statistical corrections for data from unverified samples based on the verified samples with colposcopy-directed biopsy in nine studies^41,42,44,45,47,48,50,51,53^ and colposcopy and selective biopsy in nine studies^62–70^. See Supplementary results and Supplementary Tables S1–S3 for more details on study, test, and reference standard characteristics.

**Table 2:** Study, participant, and screening test characteristics.

First author and publication year	Country	Enrollment year	N	Target age	Cytologic test	HPV assay
**Histology-based studies**						
Belinson (2001)^36–38^	China	1999	1997	35–45	LBC	HC2
Cárdenas-Turanzas (2008)^39^	USA; Canada	1998–2005	957	≥ 30	CC	HC2
Hovland (2010)^40^	Congo	2003	313	25–60	CC; LBC	PCR
**Histology-based correction studies**						
Schneider (2000)^41^	Germany	1996–1998	4761	18–70	CC	PCR
Kulasingam (2002)^42,43^	USA	1997–2000	4075	18–50	LBC	HC2
Bigras (2005)^44^	Switzerland	2002–2004	13,842	17–93	LBC	HC2
Mayrand (2007)^45,46^	Canada	2002–2004	10,154	30–69	CC	HC2
Li (2009)^47^	China	2004–2005	2562	15–59	LBC	HC2
Castle (2011)^48,49^	USA	2008–2009	41,026	25–93	LBC	HPV16/18
Mahmud (2012)^50^	Congo	2003–2004	1528	≥ 30	CC	HC2
Sangrajrang (2017)^51,52^	Thailand	2014–2015	5046	30–60	CC	PCR; mRNA; HPV16/18
Kurokawa (2018)^53^	Japan	2015–2016	7585	25–69	LBC	PCR; HPV16/18
**Colposcopy-based studies**						
Blumenthal (2001)^54,55^	Zimbabwe	1995–1997	2073	25–55	CC	HC2
Coste (2003)^56,57^	France	1999–2000	1324	18–	CC; LBC	HC2
Sankaranarayanan (2004)^58^	India	1999–2003	18,085	25–65	CC	HC2
Qiao (2008)^59^	China	2007	2388	30–54	LBC	HC2
McAdam (2010)^60^	Vanuatu	2006	494	30–50	CC	HC2
Quincy (2012)^61^	Nicaragua	ND	245	25–60	LBC	HC2
**Colposcopy-based correction studies**						
Cuzick (2003)^62^	UK	1998–2001	10,358	30–60	CC	HC2
Petry (2003)^63^	Germany	1998–2000	7908	≥ 30	CC	HC2
Gravitt (2010)^64^	India	2005–2007	2331	≥ 25	CC	HC2
Moy (2010)^65^	China	2003–2006	9057	30–54	LBC	HC2
Monsonego (2011)^66^	France	2008–2009	4429	20–65	LBC	HC2; mRNA
Ferreccio (2013)^67^	Chile	2009–2010	8265	25–64	CC	HC2
Agorastos (2015)^68^	Greece	2011–2013	3993	25–55	LBC	PCR
Iftner (2015)^69^	Germany	ND	9451	30–60	LBC	HC2; mRNA; HPV16/18
Wu (2017)^70^	China	2015	11,064	21–65	LBC	PCR

*CC* conventional cytology, *HC2* Hybrid Capture 2, *HPV* human papillomavirus, *LBC* liquid-based cytology, *mRNA* messenger ribonucleic acid, *PCR* polymerase chain reaction.

### Risk of bias

Although the studies were predominantly well conducted, their designs varied substantially, and several sources of bias were observed (Supplementary Fig. S2), such as lack of blinding of the colposcopists or grading pathologists to the screening results. Additionally, verification bias could not be ruled out in studies that did not perform histological evaluation of all samples.

### Topology of direct comparisons of alternative screening algorithms

**Figure 1:**
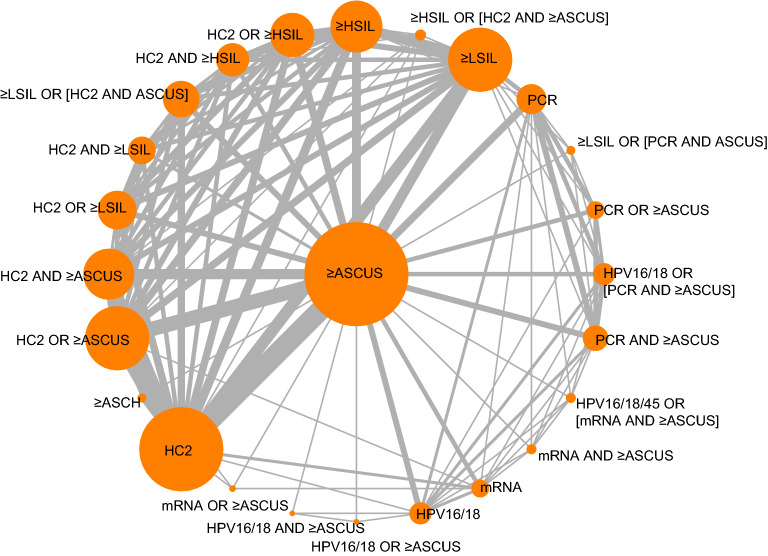
Network of eligible comparisons of cervical cancer screening algorithms. The line thickness is proportional to the number of studies comparing the linked pair of screening algorithms. The size of each node is proportional to the number of study participants. *ASCH* atypical squamous cells cannot exclude high-grade lesion, *ASCUS* atypical squamous cells of undetermined significance, *HC2* Hybrid Capture 2, *HPV16/18(/45)* genotyping for HPV types 16 or 18 (or 45), *HSIL* high-grade squamous intraepithelial lesion, *LBC* liquid-based cytology, *LSIL* low-grade squamous intraepithelial lesion, *mRNA* messenger ribonucleic acid, *PCR* polymerase chain reaction.

Figure 1 shows the network of compared algorithms available from the 27 studies, and Supplementary Table S4 shows the numbers of studies and participating women contributing to each comparison. From 25 screening strategies, 300 pairwise comparisons are theoretically constructable. However, the 27 studies provided 337 contrast data (median 6 [min–max, 1–55] contrasts per study) on only 123 unique pairwise comparisons (41% of all theoretically constructable contrasts). A comparison was based on a median of two studies (min–max, 1–14), and only 18 (15%) of 123 comparisons were based on five or more studies. The three most common comparisons were derived from studies that assessed HC2 and ≥ ASCUS; that is, the comparisons on standalone HC2 *vs.* standalone ≥ ASCUS (14 studies; 84,330 women), ≥ ASCUS alone *vs.* HC2 OR ≥ ASCUS (10 studies; 53,337 women), and HC2 alone *vs.* HC2 OR ≥ ASCUS (10 studies; 53,303 women).

### Sensitivity and specificity

The sensitivity estimates varied substantially across studies with broad confidence intervals (CIs); the specificity values also varied although their CIs were narrow (Supplementary Fig. S3). Large between-study heterogeneity was visually noted in studies of HC2, all thresholds of cytological testing, and their combinations. These results were also reflected in large credible and predictive regions of the average sensitivity and specificity in the separately performed standard bivariate meta-analyses (Supplementary Fig. S4). Although data points were limited, heterogeneity was less prominent in PCR and PCR-based combinations. See Supplementary Fig. S5 for the average estimates of screening accuracy based on the standard meta-analysis.

**Figure 2:**
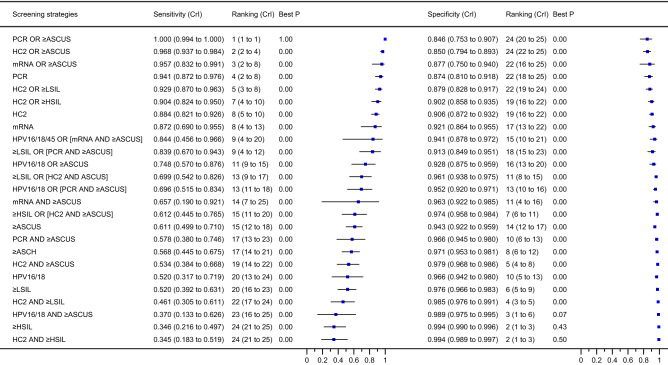
Average sensitivity and specificity and ranking of standalone tests and combination algorithms for cervical cancer screening for detecting CIN2+. Point estimates (blue squares) and CrIs (extending lines) are presented (ordered by the ranking of each test/combination’s sensitivity). See Table 1 for the definition of each strategy. *ASCH* atypical squamous cells cannot exclude high-grade lesion, *ASCUS* atypical squamous cells of undetermined significance, *CrI* 95% credible interval, *HC2* Hybrid Capture 2, *HPV16/18(/45)* genotyping for HPV types 16 or 18 (or 45), *HSIL* high-grade squamous intraepithelial lesion, *LBC* liquid-based cytology, *LSIL* low-grade squamous intraepithelial lesion, *mRNA* messenger ribonucleic acid, *PCR* polymerase chain reaction.

Figure 2 provides the average accuracy estimates and ranking estimated through the network meta-analysis. Overall, the combinations with the OR rule of hrHPV and cytological testing were most sensitive and least specific, whereas combinations with the AND rule of hrHPV and cytological testing were most specific and least sensitive. The rankings estimated in the network meta-analysis reflected the trade-off between sensitivity and specificity by altering the thresholds; lowering the thresholds of cytological testing (e.g., from ≥ HSIL to ≥ ASCUS) led to higher sensitivity but at the cost of reduced specificity, and tightening the thresholds increased specificity at the cost of reduced sensitivity. This behavior resulted in average estimates and rankings for tests or combination algorithms relying on few studies (e.g., HPV16/18- and mRNA-based combinations assessed in only one study each), which were inconsistent with the standard meta-analysis.

In the network meta-analysis, PCR OR ≥ ASCUS was most sensitive (1.0; CrI: 0.994–1.0; probability of best sensitivity: 1.0) but was one of the two least specific screening algorithms (0.846; CrI: 0.753–0.907). In contrast, standalone ≥ HSIL and HC2 AND ≥ HSIL were the two most specific (respectively, 0.994 [CrI: 0.990–0.996; probability of best specificity: 0.43] and 0.994 [CrI: 0.989–0.997]; probability of best specificity: 0.50) but were the two least sensitive (respectively, 0.346 [95% CrI: 0.216–0.497] and 0.345 [CrI: 0.183–0.519]) algorithms.

### Comparative accuracy

Supplementary Figure S6, Supplementary Tables S5 and S6, respectively, summarize the average relative sensitivity and specificity and ΔFNs and ΔFPs estimated based on a population of 1000 healthy women, with a 2% prevalence of CIN2+, across all possible paired comparisons of available standalone tests and combination algorithms.

### Comparative accuracy of standalone tests

**Figure 3:**
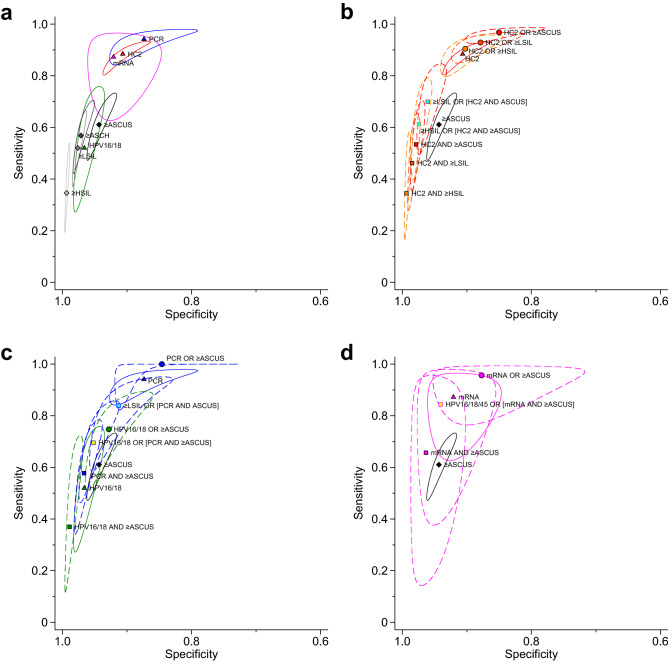
Network meta-analysis of standalone tests and combination algorithms for cervical cancer screening for detecting CIN2+. Average sensitivity and specific and their 95% credible regions for (**a**) standalone cytology or hrHPV testing, (**b**) HC2-based combination algorithms, (**c**) PCR-based combination algorithms (including PCR-based genotyping for HPV16/18), and (**d**) mRNA-based combination algorithms (including mRNA-based genotyping for HPV16/18/45). Graded colors (black, dark gray, gray, and light gray) indicate cytology with a specific threshold, red indicates HC2, blue indicates PCR-based tests, green indicates HPV16/18, and magenta indicates mRNA-based tests. Triangles and diamonds represent standalone hrHPV testing and cytology, respectively. Circles and squares represent combinations based on the OR-rule and the AND-rule, respectively. For combination algorithms (**b–d**), standalone component hrHPV testing and cytology (≥ ASCUS) are also presented as reference. See Table 1 for the definition of each strategy. *ASCH* atypical squamous cells cannot exclude high-grade lesion, *ASCUS* atypical squamous cells of undetermined significance, *HC2* Hybrid Capture 2, *HPV16/18(/45)* genotyping for HPV types 16 or 18 (or 45), *HSIL* high-grade squamous intraepithelial lesion, *LBC* liquid-based cytology, *LSIL* low-grade squamous intraepithelial lesion, *mRNA* messenger ribonucleic acid, *PCR* polymerase chain reaction.

For cytological testing, the average relative estimates of screening accuracy reflected the effect of altering the thresholds (Fig. 3a, Supplementary Table S7). For example, ≥ ASCUS was more sensitive than ≥ LSIL (relative sensitivity: 0.86 [CrI: 0.69–0.97; Bayesian *P*(≥ 1) < 0.001]) but less specific than ≥ LSIL (relative specificity: 1.03 [CrI: 1.05–1.02; Bayesian *P*(≤ 1) < 0.001]). Two studies that directly compared the alternative smear preparation methods showed identical sensitivity and specificity for CC and LBC for each threshold (Supplementary Fig. S7).

HPV16/18 was more specific but less sensitive than the other hrHPV assays (Fig. 3a, Supplementary Table S7). For example, for comparing HPV16/18 with HC2, the relative specificity was 1.06 [CrI: 1.10–1.04; Bayesian *P*(≤ 1) < 0.001] and relative sensitivity was 0.59 [CrI: 0.36–0.81; Bayesian *P*(≥ 1) < 0.001]. Among HC2, PCR-based tests, and mRNA-based tests, data were limited as to whether a specific hrHPV assay was more sensitive or specific than any other. For example, although the PCR-based tests appeared more sensitive but less specific than HC2, the CrIs for the relative accuracy crossed 1, the null value (i.e., the relative sensitivity of PCR *vs.* HC2 was 1.06 [CrI: 0.98–1.15]; Bayesian *P*(≤ 1) = 0.06) and relative specificity of HC2 *vs.* PCR was 1.04 [CrI: 0.99–1.11; Bayesian *P*(≤ 1) = 0.08]).

Compared with standalone cytological testing irrespective of the thresholds, all standalone hrHPV assays other than HPV16/18 were more sensitive but less specific in general (Fig. 3a, Supplementary Table S7). In contrast, the accuracy of HPV16/18 was comparable to cytological testing. For example, the relative specificity for comparing ≥ LSIL with HPV16/18 was 1.0 [CrI: 0.68–1.62; Bayesian *P*(≥ 1) = 0.50] and relative specificity was 1.01 (CrI: 1.00–1.03; Bayesian *P*(≤ 1) = 0.10).

### Comparative accuracy among combination algorithms based on specific hrHPV assays

The ROC plots of the average accuracy estimates and their credible regions reflected the effect of altering the thresholds in combined cytological testing (i.e., lower thresholds with increased sensitivity and decreased specificity, and higher thresholds with increased specificity and decreased sensitivity) and the effect of combination methods (i.e., the OR rule with increased sensitivity and decreased specificity, and the AND rule with increased specificity and decreased sensitivity) across the subgroups based on alternative hrHPV assays (Fig. 3b–d). Among 45 pairwise comparisons based on cytology, HC2, and their combinations, most (40 [89%] for sensitivity and 42 [93%] for specificity) showed a significant difference, reflecting the effect of the thresholds and combination methods (Fig. 3b, Supplementary Table S8). Similarly, among 36 pairwise comparisons based on cytology, PCR-based tests, and their combinations, 28 (78%) for sensitivity and 27 (75%) for specificity showed a significant difference (Fig. 3c, Supplementary Table S9). In contrast, 10 pairwise comparisons based on mRNA-based combinations (Fig. 3d, Supplementary Table S10), only five (50%) and four (40%) contrasts for sensitivity and specificity, respectively, were significantly different.

### Comparative accuracy and GRADE assessment of guideline-proposed combination algorithms

Data on the guideline-proposed algorithms are available for HC2 and PCR-based tests on “≥ LSIL OR [hrHPV AND ASCUS]” and for mRNA-based tests and PCR-based tests on “HPV16/18(/45) OR [hrHPV AND ≥ ASCUS]”. Table 3 summarizes the comparative accuracy, and Supplementary Table S11 and Table 4 show the GRADE summary of findings on specific tests or combination algorithms and their comparisons, respectively.

**Table 3:** Comparative accuracy of guideline-proposed combination algorithms.

Index and comparator tests or combination algorithms	Index (for specificity) and comparator (for sensitivity) tests or combination algorithms							
	PCR	HC2	mRNA	HPV16/18/45 OR [mRNA AND ≥ ASCUS]	≥ LSIL OR [PCR AND ASCUS]	≥ LSIL OR [HC2 AND ASCUS]	HPV16/18 OR [PCR AND ≥ ASCUS]	≥ ASCUS
**Index (for sensitivity) and comparator (for specificity) tests or combination algorithms**								
PCR	–	0.94 (0.87 to 1.02) [0.06]	0.93 (0.73 to 1.04) [0.10]	0.90 (0.49 to 1.05) [0.12]	0.89 (0.72 to 1.01) [0.04]	0.75 (0.58 to 0.89) [< 0.001]	0.74 (0.56 to 0.89) [< 0.001]	0.65 (0.54 to 0.75) [< 0.001]
HC2	1.04 (0.99 to 1.11) [0.08]	–	0.99 (0.79 to 1.10) [0.42]	0.96 (0.52 to 1.11) [0.35]	0.95 (0.76 to 1.08) [0.26]	0.79 (0.63 to 0.93) [0.001]	0.79 (0.59 to 0.95) [0.003]	0.69 (0.58 to 0.79) [< 0.001]
mRNA	1.05 (0.99 to 1.13) [0.05]	1.02 (0.96 to 1.06) [0.26]	–	0.97 (0.53 to 1.24) [0.41]	0.97 (0.77 to 1.24) [0.37]	0.81 (0.62 to 1.05) [0.049]	0.81 (0.59 to 1.05) [0.049]	0.71 (0.58 to 0.89) [0.005]
HPV16/18/45 OR [mRNA AND ≥ ASCUS]	1.07 (1.00 to 1.16) [0.02]	1.04 (0.97 to 1.08) [0.10]	1.02 (0.96 to 1.08) [0.23]	–	1.00 (0.77 to 1.82) [0.49]	0.84 (0.62 to 1.54) [0.19]	0.84 (0.60 to 1.50) [0.19]	0.73 (0.58 to 1.33) [0.09]
≥ LSIL OR [PCR AND ASCUS]	1.04 (0.97 to 1.12) [0.10]	1.01 (0.94 to 1.05) [0.39]	0.99 (0.92 to 1.06) [0.40]	0.97 (0.91 to 1.04) [0.18]	–	0.84 (0.64 to 1.08) [0.08]	0.84 (0.65 to 0.97) [< 0.001]	0.73 (0.59 to 0.92) [0.004]
≥ LSIL OR [HC2 AND ASCUS]	1.10 (1.05 to 1.18) [< 0.001]	1.06 (1.03 to 1.09) [< 0.001]	1.04 (1.00 to 1.11) [0.02]	1.02 (0.98 to 1.09) [0.16]	1.05 (1.01 to 1.13) [0.007]	–	1.00 (0.72 to 1.33) [0.49]	0.88 (0.71 to 1.10) [0.11]
HPV16/18 OR [PCR AND ≥ ASCUS]	1.09 (1.04 to 1.16) [< 0.001]	1.05 (1.02 to 1.09) [0.004]	1.03 (0.99 to 1.09) [0.06]	1.01 (0.97 to 1.08) [0.29]	1.04 (1.01 to 1.11) [< 0.001]	0.99 (0.96 to 1.02) [0.25]	–	0.88 (0.71 to 1.16) [0.16]
≥ ASCUS	1.08 (1.03 to 1.15) [< 0.001]	1.04 (1.02 to 1.07) [< 0.001]	1.02 (0.99 to 1.08) [0.10]	1.00 (0.97 to 1.07) [0.45]	1.03 (0.99 to 1.10) [0.06]	0.98 (0.96 to 1.00) [0.04]	0.99 (0.97 to 1.02) [0.23]	–

Above the diagonal line (formed by cells with an en dash) represents relative sensitivity (95% CrI) [probability that relative sensitivity is ≥ 1] and below the diagonal line represents relative specificity (95% CrI) [probability that relative specificity is ≤ 1]. For relative sensitivity, the rows and columns, respectively, represent the index (the test of interest) and comparator (the test in comparison) tests or combination algorithms. For relative sensitivity, the columns and rows, respectively, represent the index and comparator tests or combination algorithms.

*ASCUS* atypical squamous cells of undetermined significance, *CrI* credible interval, *HC2* Hybrid Capture 2, *HPV16/18(/45)* genotyping for HPV types 16 or 18 (or 45), *HSIL* high-grade squamous intraepithelial lesion, *LBC* liquid-based cytology, *LSIL* low-grade squamous intraepithelial lesion, *mRNA* messenger ribonucleic acid, *PCR* polymerase chain reaction.

**Table 4:**
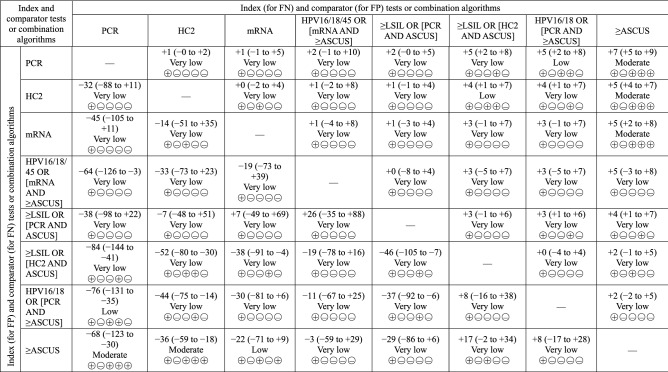
The GRADE summary of findings table for comparative evidence.

Above the diagonal line (formed by cells with an em dash) represents the number of the difference in (Δ) FNs (95% CrI) and below the diagonal line represents Δ FPs (95% CrI). For Δ FPs, the rows and columns, respectively, represent the index (the test of interest) and comparator (the test in comparison) tests or combination algorithms. For Δ FNs, the columns and rows, respectively, represent the index and comparator tests or combination algorithms. Results are based on a healthy screening population of 1000 women in which 20 are CIN2+ (2%).

*ASCUS* atypical squamous cells of undetermined significance, *CIN2*+*, CrI* cervical intraepithelial neoplasia grade 2 or higher grades; 95% credible interval, *FN* false negative, *FP* false positive, *GRADE* Grading of Recommendations Assessment, Development and Evaluation, *HC2* Hybrid Capture 2, *HPV16/18(/45)* genotyping for HPV types 16 or 18 (or 45), *HSIL* high-grade squamous intraepithelial lesion, *LBC* liquid-based cytology, *LSIL* low-grade squamous intraepithelial lesion, *mRNA* messenger ribonucleic acid, *PCR* polymerase chain reaction, *TN* true negative, *TP* true positive.

In general, the proposed algorithms were less sensitive but more specific than the standalone component hrHPV assays. However, only HC2-based “≥ LSIL OR [hrHPV AND ASCUS]” and PCR-based “HPV16/18 OR [hrHPV AND ≥ ASCUS]” were significantly less sensitive (the average relative sensitivity ranged from 0.74 to 0.79; Bayesian *P*(≥ 1) ranged from < 0.001 to 0.003) and more specific (the average relative specificity ranged from 1.04 to 1.10; Bayesian *P*(≤ 1) ranged from < 0.001 to 0.004). These results suggested that the proposed algorithms, compared with their standalone component hrHPV tests, decreased by an average of 44 to 88 FPs but increased 4 to 5 more FNs (very low to low certainty of evidence).

In contrast, the proposed algorithms were in general equally specific but more sensitive than standalone ≥ ASCUS. However, only PCR-based “LSIL OR [hrHPV AND ASCUS]” was significantly less sensitive than ≥ ASCUS alone (the relative sensitivity = 0.73 [CrI: 0.59–0.92; Bayesian *P*(≥ 1) = 0.004]; four more FNs [CrI: 1–7]; very low certainty of evidence), but evidence as to whether this combination was more specific or less specific than ≥ ASCUS alone was insufficient (relative sensitivity = 0.98 [CrI: 0.96–1.00; Bayesian *P*(≥ 1) = 0.04]).

Comparative evidence across alternative guideline-proposed algorithms was generally limited. PCR-based “LSIL OR [hrHPV AND ASCUS]” was significantly more specific and less specific than “HPV16/18 OR [hrHPV AND ≥ ASCUS]” (relative sensitivity; 1.04 [CrI: 1.01–1.11]; Bayesian *P*(≤ 1) < 0.001]; 37 fewer FPs [CrI: 6–92] and relative specificity: 0.84 [CrI: 0.65–0.97]; Bayesian *P*(≥ 1) < 0.001; three more FNs [CrI: 1–6]; very low certainty of evidence). Although only HC2-based “LSIL OR [hrHPV AND ASCUS]” was more specific than PCR-based “LSIL OR [hrHPV AND ASCUS]” (relative specificity: 1.05 [CrI: 1.01–1.13]; Bayesian *P*(≤ 1) = 0.007; 46 fewer FPs [CrI: 7–105]; very low certainty of evidence) across-hrHPV assays, comparative data on the guideline-proposed algorithms were insufficient.

### Meta-regression and sensitivity analyses

Due to data paucity, meta-regression was undertaken for only HC2, cytological testing, and their OR combination separately. Although high-income countries (*vs.* non-high-income countries) for sensitivity of HC2 and sample collection by physicians (*vs.* nonphysician collectors) for sensitivity and specificity of ≥ ASCUS were associated with higher estimates, these covariates were no longer associated with higher (or lower) sensitivity or specificity in their combination, HC2 OR ≥ ASCUS (Supplementary Fig. S8).

The sensitivity analysis using the model with a common correlation parameter across tests yielded results comparable to those of the main analysis based on the model with test-specific correlation parameters (Supplementary Table S12). Relaxing threshold constraints yielded results not compliant with the expected threshold effects in two specific thresholds for cytological testing (≥ LSIL and ≥ ASCH) and unstable results with wide CrIs for sensitivity in four combination algorithms (i.e., mRNA AND ≥ ASCUS, HPV16/18 AND ≥ ASCUS, HPV16/18 OR ≥ ASCUS, “ ≥ LSIL OR [PCR AND ASCUS]”, and “≥ HSIL OR [HC2 AND ≥ ASCUS]”) regardless of whether correlation parameters were separately assumed or not; all of these tests, except for ≥ LSIL, depended on only a few primary studies. With lower deviance information criterion estimates, the models with threshold constraints were deemed to be better-fitting than the models without threshold constraints; however, the differences were < 5, suggesting no definitively preferred model.

## Discussion

To the best of our knowledge, this is the first network meta-analysis that has comprehensively compared and ranked the cross-sectional screening accuracy of standalone cytology or hrHPV testing with combination algorithms for detecting CIN2+. Importantly, this analysis is based on published accuracy estimates from fully paired-design comparative accuracy studies that addressed verification bias. First, our network meta-analysis confirmed and quantified the theoretically expected gain in and trade-off of screening performance when combining two tests^24^, that is, the combinations with the OR rule (i.e., either test positive) of hrHPV and cytological testing were most sensitive and least specific, whereas combinations with the AND rule (i.e., both test positive) of hrHPV and cytological testing were most specific and least sensitive. Second, our network meta-analysis confirmed that the guideline-proposed combination algorithms, HC2-based “≥ LSIL OR [hrHPV AND ASCUS]” and PCR-based “HPV16/18 OR [hrHPV AND ≥ ASCUS]” appeared to compensate the shortcomings of the two component tests if used as standalone, which, though expected theoretically, had never been quantitatively synthesized. Specifically, these proposed algorithms were not as sensitive but more specific than the component standalone hrHPV testing. Similarly, these proposed algorithms appeared equally specific but more sensitive than standalone ≥ ASCUS, though definitive conclusions could not be made due to limited comparative data. Third, sparse, insufficient comparative evidence precluded reliable assessment of the comparative accuracy across these alternative guideline-proposed algorithms.

Effectiveness of screening should be assessed as a whole program consisting of a set of activities^71^. Since the ultimate goal is to maximize participant-relevant benefits and simultaneously minimize harms, accuracy of testing is, though an important measure, only an intermediate parameter. As already elucidated in the previous meta-analyses^13,14^, which is congruent with our results, standalone testing for hrHPV using an assay other than HPV 16/18 genotyping, if all screen-positive women underwent colposcopy, would identify more women with CIN2+ than cytological testing alone but at the cost of more healthy women misclassified as CIN2+. The OR rule combinations, the most sensitive group of strategies found in our meta-analysis, if used for primary co-testing (i.e., performing both tests concurrently), would further increase the number of healthy women misclassified as CIN2+ while identifying only a few more women with CIN2+. The consequences of such FP results include unnecessary colposcopy, triage, or repeat testing with cytology, hrHPV, or other tests. Although infections with hrHPV, and HPV16/18 in particular, carry a higher risk of progression than positive cytology^72–75^, immediate incremental costs and psychological burden incurred due to increased false-positive results may not be justified in low risk screening settings as only a fraction of the identified CIN2+ lesions detected through standalone hrHPV testing or its combinations progress to invasive cancer; the others actually carry a moderate chance of regression^76^. The AND rule combinations, the most specific group of strategies identified in our meta-analysis, may substantially minimize FPs and their negative consequences. However, sensitivity is lower than cytology alone (≥ ASCUS), potentially leading to unignorably large numbers of FNs depending on the prevalence of CIN2+ in a screened population.

As interim recommendations, several protocols for triage and/or repeat testing followed by colposcopy for screen-positive women have been proposed by professional societies. “≥ LSIL OR [hrHPV AND ASCUS]” and “HPV16/18 OR [hrHPV AND ≥ ASCUS]” were cross-sectional representations for two such protocols, respectively, proposed for positive primary cytological testing^11^ and primary hrHPV testing^9^. Our meta-analysis found that the accuracy of these combination algorithms were generally ranked in the middle, being more sensitive and less specific than standalone cytology (≥ ASCUS) and the AND rule combinations but more specific and less sensitive than standalone hrHPV testing and the OR rule combination. We also quantified how each combination algorithm increased or decreased the number of FNs and FPs relative to those of another specific standalone test or combination, which is a strength of our study results. However, any benefits and harms associated with specific screening tests or combinations should be formally assessed at the whole program level along with its necessary resources and costs^71^.

We focused on cross-sectional accuracy of initial screening tests or combinations and their immediate consequences. Our accuracy-based arguments necessarily lack long-term outcomes. Given the chance of regression^76^, the results based on our cross-sectional approach may be only relevant in populations with a low participation rate of follow-up testing. Additionally, the positive criteria we adopted for the estimation of accuracy do not necessarily represent the optimal indications of colposcopy in real-life practice; rather the criteria included the joint indications of any additional intervention; i.e., triage and/or repeat testing, colposcopy, and immediate direct treatments jointly. In this regard, a recent expert consensus statement proposed individualized risk-based management decisions based on the combinations of the available screening results^77^.

Colposcopy-directed biopsy is an imperfect test even for routine biopsies on normal-appearing sites^78^ and more so for colposcopy and selective biopsy^79^. Despite the theoretical superiority of verification bias-corrected accuracy estimates over naïvely calculated estimates, these corrections are not error-free. Given the complex mechanisms of missing verification^80^ and limitations in inverse probability weighting^81^, bias may not necessarily have been corrected in the right direction. In addition, the effect of the excluded observations due to unsatisfactory or missing test results, even though the reported proportions were not substantial, could be unpredictably large. Furthermore, our meta-analysis was based on aggregate data and thus only accounted for the dependence of the two tests at the aggregate data level^82^; however, a more sophisticated approach to address these limitations would require individual-level data.

Our GRADE assessment used a typical population-based screening context in high-income countries as adopted in a previous review^13^; however, the large spread of the credible and predictive accuracy values in our study suggests wide-ranging real-life variations, implying that specific scenarios with different risks might yield divergent conclusions. Finally, we did not assess combinations involving newer screening modalities, such as p16/Ki-67 dual-stain-based cytology^83^, as this was beyond the scope of our meta-analysis.

## Conclusions

Limited evidence suggests that specific test combinations might complement the weaknesses of standalone cytological or hrHPV screening and help reduce FN and/or FP results. However, the strategies that provide more benefits than harms at reasonable cost in a population need to be assessed at the program level. As comparative evidence on alternative hrHPV assays is sparse, further research is needed to acquire relevant data. Additionally, future research should elucidate long-term outcomes of specific algorithms and acquire data from HPV-vaccinated populations.

## Supplementary Information

Supplementary Information.

## Data Availability

The data and statistical codes that supports the findings of this study will be shared on reasonable request to the corresponding author.
