# Supplementary material for: Comparative accuracy of cervical cancer screening strategies in healthy asymptomatic women: a systematic review and network meta-analysis
Source: Sci Rep. 2022 Jan 7;12:94. doi: 10.1038/s41598-021-04201-y (PMC8741996; doi:10.1038/s41598-021-04201-y)
Supplement: Supplementary file 1 — Supplementary Information. [file 41598_2021_4201_MOESM1_ESM.pdf]

Online-Only Supplementary Material

Terasawa T, Hosono S, Sasaki S, Hoshi K, Hamashima Y, Katayama T, Hamashima C.
Comparative accuracy of cervical cancer screening strategies in healthy asymptomatic women: a systematic review and network meta-analysis

ONLINE-ONLY SUPPLEMENTARY METHODS 3
Search Strategy 3
Inclusion criteria 7
Data extraction 8
Data synthesis 9
ONLINE-ONLY SUPPLEMENTARY RESULTS 11
Study Characteristics 11
Test Characteristics 11
Reference Standard Characteristics 12
ONLINE-ONLY SUPPLEMENTARY TABLES 13
Table S1. Study Characteristics 13
Table S2. Test Characteristics 18
Table S3. Reference Standard Characteristics 29
Table S4. Comparative Evidence of Cervical Cancer Screening Tests or Combination Algorithms.* 40
Table S5. Comparative Accuracy of Cervical Cancer Screening Tests or Combination Algorithms.* 43
Table S6. Difference in False Negatives or Positives between Alternative Cervical Cancer Screening Tests or Combination Algorithms.* 49
Table S7. Comparative Accuracy of Standalone Tests.* 54
Table S8. Comparative Accuracy of HC2-based Combinations.* 55
Table S9. Comparative Accuracy of PCR-based Combinations.* 57
Table S10. Comparative Accuracy of mRNA-based Combinations.* 59

Table S11. The GRADE Summary of Findings Table for Standalone Tests and Combination Algorithms. 60

Table S12. Sensitivity Analysis based on Alternative Models. 62

ONLINE-ONLY SUPPLEMENTARY FIGURES 65

Figure S1. PRISMA Flow Diagram 65

Figure S2. Risk of Bias and Concerns Regarding the Applicability of the Included Studies 67

Figure S3. Cross-hair Plots of Standalone Tests and Combination Algorithms. 68

Figure S4. Standard Meta-analysis of Screening Accuracy of Standalone Tests and Combination Algorithms. 69

Figure S5. Summary of Standard Meta-analysis of Screening Accuracy. 72

Figure S6. Comparison of Screening Accuracy Among Alternative Tests and/or Combination Algorithms 77

Figure S7. Comparison of Screening Accuracy Among Alternative Preparation Methods for Cytologic Testing 78

Figure S8. Modifiers of Sensitivity and Specificity 79

ONLINE-ONLY REFERENCES 80

LIST OF EXCLUDED PUBLICATIONS (n = 224) 84

ONLINE-ONLY SUPPLEMENTARY METHODS

To update the Japanese Guideline for Cervical Cancer Screening 2010,[1] we conducted a broad systematic evidence review of the effectiveness of currently implemented and/or proposed cervical cancer screening programs.[2] Comparisons of the sensitivity and specificity of alternative testing strategies, standalone cervical cytological testing and testing for high-risk HPV genotypes (hrHPV), and their combination methods (based on the simple, either-positive and both-positive rules) as the primary screening modality in healthy community-dwelling asymptomatic women was one of a series of key clinical questions formulated by the evidence review team.

Search Strategy

Ovid MEDLINE

1 'Uterine Cervical Neoplasms'.mp.
2 'Uterine Cervical Dysplasia'.mp.
3 'Cervical Intraepithelial Neoplasia'.mp.
4 cervix.mp.
5 cervical.mp.
6 cervico*.mp.
7 4 or 5 or 6
8 cancer*.mp.
9 carcinoma.mp.
10 adenocarcinoma.mp.
11 neoplas*.mp.
12 dysplas*.mp.
13 dyskaryos*.mp.
14 squamous.mp.
15 CIN.mp.
16 CINII*.mp.
17 CIN2*.mp.
18 CINIII*.mp.
19 CIN3*.mp.
20 SIL.mp.
21 HSIL.mp.
22 H-SIL.mp.
23 LSIL.mp.
24 L-SIL.mp.
25 ASCUS.mp.

26 AS-CUS.mp.

8 or 9 or 10 or 11 or 12 or 13 or 14 or 15 or 16 or 17 or 18 or 19 or 20 or 21 or 22 or 23 or
27 24 or 25 or 26

28 7 and 27

29 1 or 2 or 3 or 28

30 papillomaviridae.mp.

31 alphapapillomavirus.mp.

32 'DNA, viral'.mp.

33 'Papillomavirus Infections'.mp.

34 'Tumor Virus Infections'.mp.

35 Cervix Uteri/virology.mp.

36 HPV.mp.

37 'human papillomavirus'.mp.

38 papillomaviridae.mp.

39 PCR.mp.

40 'hybrid capture*'.mp.

41 HC2.mp.

42 HCII.mp.

43 'HC 2'.mp.

44 'HC II'.mp.

45 viral.mp.

46 virolog*.mp.

47 45 or 46

48 DNA.mp.

49 47 and 48

50 30 or 31 or 32 or 33 or 34 or 35 or 36 or 37 or 38 or 39 or 40 or 41 or 42 or 43 or 44 or 49

51 29 and 50

52 screen*.mp.

53 test*.mp.

54 population*.mp.

55 surveillance.mp.

56 54 and 55

57 early.mp. or "Early Detection of Cancer"/

58 detect*.mp.

59 57 and 58

60 prevent*.mp.

61 57 and 60

62 triage.mp.

63 management.mp.

64 'follow up'.mp.

65 follow-up.mp.

66 64 or 65

67 52 or 53 or 56 or 59 or 61 or 62 or 63 or 66

68 51 and 67

69 limit 68 to yr="1992 -Current"

70 limit 69 to humans

71 limit 69 to animals

72 71 not 70

73 69 not 72

74 vaccin*.ti.

75 73 not 74

76 limit 75 to comment

77 75 not 76

78 limit 77 to "newborn infant birth to 1 month"

79 77 not 78

80 limit 79 to "infant 1 to 23 months"

81 79 not 80

EMBASE

1 'cervix'/exp OR cervix

2 cervic*

3 #1 OR #2

4 'cancer'/exp OR cancer

5 'carcinoma'/exp OR carcinoma

6 'neoplasia'/exp OR neoplasia

7 'dysplasia'/exp OR dysplasia

8 cin*

9 sil*

10 h sil'

11 hsil

12 'l sil'

13 lsil

14	ascus
15	'asc us'
16	squamous
17	dyskaryos*
18	neoplas*
19	'adenocarcinoma'
20	#4 OR #5 OR #6 OR #7 OR #8 OR #9 OR #10 OR #11 OR #12 OR #13 OR #14 OR #15 OR #16 OR #17 OR #18 OR #19
21	#3 AND #20
22	screen*
23	test*
24	population*
25	'surveillance'/exp OR surveillance
26	#24 AND #25
27	early
28	detect*
29	#27 AND #28
30	prevent*
31	#27 AND #30
32	'triage'/exp OR triage
33	'management'/exp OR management
34	'follow up'/exp OR 'follow up'
35	#22 OR #23 OR #26 OR #29 OR #31 OR #32 OR #33 OR #34
36	#3 AND #20 AND #35
37	'human papillomavirus'/exp OR 'human papillomavirus'
38	'hpv'/exp OR hpv
39	('wart'/exp OR wart) AND ('virus'/exp OR virus)
40	'hybrid'/exp OR hybrid
41	capture
42	#40 AND #41
43	hc2
44	'hc2 assay'
45	'hc 2'
46	hcii
47	'hc ii'
48	#37 OR #38 OR #39 OR #42 OR #43 OR #44 OR #45 OR #46 OR #47

49	#3 AND #20 AND #35 AND #48
50	#49 AND [1992-2018]/py
51	#50 AND [editorial]/lim
52	#50 NOT #51
53	#52 AND ('conference abstract'/it OR 'conference paper'/it OR 'conference review'/it)
54	#52 NOT #53
55	#54 AND [embase]/lim
56	#54 AND [medline]/lim
57	#55 NOT #56

Inclusion Criteria

Studies were deemed eligible if they incorporated a paired design that assessed the screening accuracy of cytological and hrHPV testing for detecting cervical intraepithelial neoplasia (CIN) grade 2 or worse (CIN2+) in healthy community-dwelling asymptomatic women. We only included primary screening studies that used acceptable reference standard tests for all women, that undertook statistical corrections for verification bias, or that provided data amenable to such statistical corrections if not all screened women were examined by an acceptable reference standard. Acceptable study designs for verification bias corrections included a design wherein samples from a portion of women testing negative for both screening tests (typically selected by random sampling) were submitted for verification by a reference standard test before statistical correction or when a study only verified samples from women with ≥1 positive screening result(s).[3] We did not specify a threshold for required levels of verification. Acceptable reference standards were histology (typically through colposcopy-directed biopsy and/or random biopsy), and a combination of colposcopic visual assessment and selective biopsy in women with protocol-specified screening and/or colposcopy results; i.e., typically, women with visible abnormal sites in the cervix on colposcopy). We defined a primary screening study as that which exclusively enrolled healthy asymptomatic women who did not display immunosuppression, including human immunodeficiency virus infection, or had a history of abnormal screening results that required repeat or triage/reflex testing before referral to colposcopy or immediate colposcopy. We accepted studies irrespective of whether they used opportunistic screening or an organized screening program. To counter verification bias, we excluded studies that only reported naïve accuracy estimates without performing statistical corrections for unverified double-screening negative women (typically assumed to be "true negative").[3] When an ineligible population was jointly analyzed with an eligible primary screening population, we only included studies from which relevant data were adequately extractable. For studies that did not permit data extraction or calculation of adjusted sensitivity and specificity estimates, we contacted the authors of the primary studies for clarification or to request unpublished data. This request was considered as

rejected if two emails, sent at least 2 weeks apart, received no response. Any discrepancies in eligible studies were discussed in a face-to-face group meeting and resolved via consensus. We took particular care to identify multiple publications of a study cohort by comparing authors, centers, and recruitment periods. In case of multiple publications, we included the publication with the largest sample size to avoid double counting.

Data Extraction

One reviewer (TT) extracted descriptive data, which were independently confirmed by another reviewer (SH or SS). We extracted the following descriptive information: study characteristics, namely, first author and publication year, study location, enrollment year, study design (prospective or retrospective collection of data), screening setting, sample size, and target and/or average age, and exclusion criteria, along with test characteristics, such as cytological method employed, liquid-based cytology (LBC) platform used, classification system and cutoff criteria for positive results; nature of sample collectors; collection devices; assessors of cytology results; high-risk human papillomavirus (hrHPV) testing platform and HPV genotypes assessed; cutoff values for positive hrHPV testing; data on screening modalities that were simultaneously assessed (if any); and the frequency of unsatisfactory or missing results. With respect to reference standard characteristics, we recorded the methods employed for colposcopy and other invasive procedures used to obtain the cervical specimens, histological evaluation, and blinding of involved participants, colposcopists (if colposcopy was performed), gynecologists (if surgical intervention was required), and pathologists for screening test results; the frequency of unsatisfactory and/or missing biopsy results; and methods used for correcting verification bias when a reference method was not performed for all participants who were screened. We recorded in detail the algorithm(s) used for verifying target lesions and extracted data on how a study was planned if colposcopy and histological verification were performed in subjects with specific screening test results, correction methods were applied for unverified cases, and sensitivity analysis was performed, if any.

Each individual was considered as the unit of analysis, and study-level aggregated numerical data on all women whose screening results had been verified by colposcopy or biopsy were extracted. Numerical data were also extracted if they were statistically corrected for verification bias when not all samples were verified by an acceptable reference standard. Two of three reviewers (TT, KT, YH) independently double-extracted numerical data. Unless unpublished data were available from the authors, any discrepancies in data extraction were resolved by consensus among the three reviewers (TT, KT, YH).

We constructed a 2 × 2-contingency table by using true positive (TP), false positive (FP), false negative (FN), and true negative (TN) classifications whereby all subjects could be appropriately categorized on the basis of whether they tested positive or negative for a single screening test (i.e., cytology alone, or hrHPV testing alone) or a combination of two tests and whether their verified result

was CIN2+ or not. When a study did not perform statistical corrections, we corrected verification bias by using inverse probability weighting[4] to reconstruct a corrected 2 × 2-contingency table if the study had explicitly provided full cross-tabulation data on screening tests results as ≥4 mutually exclusive cohorts defined on the basis of positivity in two screening tests; i.e., both positive, only cytology positive, only hrHPV positive, or both negative (all four strata were deemed essential), had performed colposcopy and biopsy, and had provided data on histological findings. The corrected values were rounded off to the nearest integer so that they were amenable to meta-analysis.

We finally extracted sensitivity and specificity estimates and their 95% confidence intervals (CIs) only if the authors had provided values corrected for verification bias and only when any form of the aforementioned numerical data were unextractable from the publication. Post-hoc corrections were required in a small number of studies that reported a corrected point estimate or the upper confidence limit of sensitivity to be 1 to deal with problems arising upon logit transformation. Specifically, in studies with a reported value of 1 for the upper confidence limit, assuming symmetrical distribution, we estimated the width of the CI based on the difference between the logit-transformed point estimate and the logit-transformed lower confidence limit estimate and multiplied it by 2 to derive the upper confidence limit and the full width of the CI on the logit scale. In case both the point and upper confidence limit estimations were reported as 1 (as seen in only one study), we operationally assumed the point estimate to be 0.9999, and the width of the CI was obtained as above. As stability analysis using 0.99 and 0.999999 did not yield substantial differences, we reported results of the main analysis using only 0.9999.

Data Synthesis

First, we calculated the average estimates of sensitivity and specificity along with their corresponding 95% credible and predictive intervals (95% CrIs and PIs, respectively) of each eligible test separately and their derived average relative risks (RRs, relative) of and absolute differences(Δ) in sensitivity and specificity for all theoretically assessable pairwise comparisons by using an arm-based, two-stage hierarchical, Bayesian bivariate random-effects network meta-analysis model proposed by Owen and colleagues.[5] This hierarchical arm-based model is an extension of the standard bivariate random-effects model for diagnostic accuracy, which additionally models (i) multiple alternative tests (ii) effects of multiple thresholds of a test on sensitivity and specificity by creating threshold constraints, and (iii) within-study correlations between multiple sensitivity and specificity data pairs from the same study. In the main analysis, we modeled a separate, test-specific, between-study, correlation parameter for each test and imposed threshold constraints assuming that higher test thresholds have an increased specificity but decreased sensitivity as originally proposed.[5] In sensitivity analysis, we repeated the same analysis not imposing threshold constraints. We also modeled a common correlation parameter across all tests in place of independent, test-specific correlation parameters as stability analysis. In all analyses, we specified a

common between-study standard deviation across tests as originally proposed.[5]

For within-study variability, we directly modeled data from the contingency table without zero-cell corrections by using standard binomial likelihood with logit link (i.e., exact arm-based likelihoods) for studies that provided count data under the assumption that the sensitivity and specificity of the reference standards were both 100% (i.e., the multinomial-normal gold standard model). For studies that only reported corrected sensitivity and specificity estimates, we plugged the pairs of logit-transformed point estimates and their variances into the bivariate model while assuming that the two estimates were normally distributed (i.e., the normal–normal model), which enabled us to jointly synthesize both forms of data in a single model through "shared parameter models".[6] We used the alternative parametrization method based on the bivariate model to construct the confidence and predictive regions for average sensitivity and specificity[7] and the standard hierarchical summary ROC (HSROC) curves.[8,9]

We performed study-level univariable meta-regression for the following three prespecified predictors when ≥10 studies were available: (i) study location (i.e., high-income countries, defined as geographical regions ranked as "very high human development" by the Human Development Index [HDI] 2017[10] *vs.* not), (ii) study design based on adopted reference standard tests and, if performed in non-verified samples, correction methods used (i.e., histology-based *vs.* colposcopy-based studies), and (iii) sample collectors involved (physicians *vs.* non-physician providers). Although we also selected age as an important predictor before analysis (i.e., studies or subgroups of studies exclusively, including women <30 years old vs. those ≥30 years old), scarce pertinent subgroup data precluded the planned analyses.

For comparison, we used a standard full Bayesian bivariate random-effects model when at least three studies were available to calculate the average estimates of sensitivity and specificity along with their corresponding 95% CrIs and PIs of each eligible test separately.[11] We used an alternative parameterization method proposed by Chu et al, which enabled modeling of the prevalence of the target condition, π, and the derivation of logit-transformed sensitivity and specificity of the index test and their variance-covariance matrix.[11] We used two correlation parameters, one between π and the sensitivity and the other between π and the specificity, assumed both to be zero, and independently fitted a univariable random-effects model for π and a bivariate random-effects model for sensitivity and specificity.[11]

In the network meta-analysis, we used non-informative prior distributions, i.e., *dnorm*(0,0.01) for logit-transformed average sensitivity and specificity and uniform distributions of *unif*(0,2) for between-study standard deviation, also as originally proposed.[5] We used a less informative prior distribution, *dnorm*(0,0.25), for logit-transformed average sensitivity and specificity and a weakly informative gamma prior distribution that covered low-to-moderate values of between-study heterogeneity, *dgamma*(2,0.5) for between-study precision in the standard separate meta-analysis of single tests, as recommended.[12] For the network meta-analysis models, we based results on three different chains and 250,000 iterations, the stored values of which were thinned by 50 after 50,000 burn-in iterations. For the standard bivariate models, at least 30,000 iterations after ≥5,000 burn-in iterations were

performed. We assessed model convergence on the basis of the Brooks–Gelman–Rubin statistic.[13]

We assessed the certainty of evidence by using the Grading of Recommendation Assessment, Development, and Evaluation (GRADE) for diagnostic tests and strategies.[14] We assumed the prevalence of CIN2+ in a typical, population-based screening scenario in high-income countries to be 2% (i.e., 20 out of 1000 healthy women) as employed in a similar previous report.[15]

We used the deviance information criterion (DIC) to compare alternative models and regarded a >5 difference in DIC scores as important.[13] We did not examine funnel plot asymmetry because the required tests did not permit valid assessment of the extent and impact of missing data.[16] All analyses were performed by using WinBUGS 1.4.3 (MRC Biostatistics Unit, University of Cambridge, Cambridge, UK) from within Stata SE 14.1 (Stata Corp, College Station, TX, USA) using the "wbs" commands.[17] P-values for all comparisons were two-tailed, and statistical significance was defined as $P < 0.05$. The extracted datasets and BUGS codes are available from the corresponding author upon request.

ONLINE-ONLY SUPPLEMENTARY RESULTS

Study Characteristics

All included studies had a prospective design, and their sample size ranged from 245 to 41,026 (**Supplementary Table S1**). Fourteen studies (52%) were from high-income countries, four studies with mixed populations provided separate data on eligible healthy women only, and four had exclusively enrolled only never-screened women. Twelve studies had also performed screening colposcopy or visual assessment.

Test Characteristics

Thirteen studies had used only CC, and 12 had adopted only LBC, whereas two other studies had used both CC and LBC (**Supplementary Table S2**). Data on type of sample collectors was available for 20 studies (74%), with physician collectors in 14 studies and nonphysician providers, typically trained nurses or midwives, in six studies.

Of the four available hrHPV testing subgroups, HC2 was the most commonly reported hrHPV assay (assessed in 20 studies; 109,245 women), whereas six studies assessed PCR-based tests (32,720 women), four genotyped for HPV16/18 (62,941 women), and three used mRNA-based tests (18,926 women), of which also genotyped for HPV16/18/45. Only six studies (22%) assessed multiple HPV assays. The studies reported the accuracy of cytological testing at various thresholds. Most commonly used was ≥ASCUS (20 studies; 153,178 women), whereas ≥ASCH was assessed in only three studies (10,491 women).

Data on one or more combination algorithm(s) were available in 19 studies (reported in 20 publications; 70%). Of these, only five HC2-based combinations were based on five or more studies. The

most commonly assessed combinations were HC2 AND ≥ASCUS, which were reported in 10 studies.

The median proportion of unsatisfactory or missing results reported in 20 studies was 1.9% for cytological testing (range, 0.2% to 10.5%; 16 studies) and 0.8% for hrHPV testing (range, 0% to 11.5%; 11 studies). Studies typically (14/20 studies, 70%) excluded unsatisfactory or missing results to perform complete case analysis (**Supplementary Table S2**).

Reference Standard Characteristics

Reference standards were used for all participants with routine colposcopy-directed biopsy in three (11%) studies, and colposcopy and selective biopsy in six (22%) studies (**Supplementary Table S3**). Other studies performed statistical corrections for data from unverified samples that were based on the verified samples with colposcopy-directed biopsy in nine (33%) studies and colposcopy and selective biopsy in nine (33%) studies. Studies most commonly performed inverse probability weighting for correcting verification bias (15/18 studies, 83%).

ONLINE-ONLY SUPPLEMENTARY TABLES

Table S1. Study Characteristics

First author and publication year (Study name)	Study location (country)	Enrollment year	Design	Setting	Sample size, assessed/eligible (potentially eligible non-participants)	Target age (average)	Exclusion criteria
Histology-based studies							
Belinson 2001;[18] Pan 2003;[19] Zhao 2010[20] (SPOCCS I)	Shanxi province (China)	1999	Prospective study	Population-based screening*	1997/2047 (ND)	35-45 (39)	Pregnancy, prior cervical screening, pelvic irradiation, or hysterectomy
Cárdenas-Turanzas 2008[21]	Texas (USA); British Columbia (Canada)	1998-2005	Prospective study	Screening†	957/1000 (ND)	30- (47)	Pregnancy; previous abnormal Papanicolaou smear results[1]
Hovland 2010[22]	South Kivu province (Congo)	2003	Prospective study	Population-based screening	313/343 (ND)	25-60 (37)	Pregnancy; severe gynecological bleeding; hysterectomy
Histology-based correction studies							
Schneider 2000[23]	East Thurringia (Germany)	1996-1998	Prospective study	Screening	4761/5455 (ND)	18-70 (35)	Pregnancy; previous cervical conization, hysterectomy, CIN, or atypical cytology in a cervical smear ≤1 year
Kulasingam 2002;[24] Balasurbramanian 2010[25]	Washington State (USA)	1997-2000	Prospective study	Routine screening	4075/4358 (ND)	18-50 (25)	Hysterectomy; immune suppression; therapies for cervical neoplasia
Bigras 2005[26]	Multi-cantons (Switzerland)	2002-2004	Prospective study	Yearly screening	13842/13865 (ND)	17-93 (44)	STD clinic; “high-risk” populations

First author and publication year (Study name)	Study location (country)	Enrollment year	Design	Setting	Sample size, assessed/eligible (potentially eligible non-participants)	Target age (average)	Exclusion criteria
Mayrand 2007[27] (CCCaST)	Quebec and Newfoundland provinces (Canada)	2002-2004	Prospective study based on an RCT cohort	Routine screening	10154/10154 (1268)	30-69 (45)	Attending colposcopy clinic; current treatment or follow-up of a cervical lesion; no cervix; pregnancy; history of invasive cervical cancer; previous Pap test within a year
Li 2009[28] (IARC 1-3)	Shanxi, Liaoning, and Guangdong provinces (China)	2004-2005	Prospective study	Population-based screening	2562/2600 (ND‡)	15-59 (ND)	ND
Castle 2011[29] (ATHENA study)	Multi-states (USA)	2008-2009	Prospective study	Routine screening	41026/41955 (ND)	25-93 (42)	Hysterectomy; pregnancy; treatment for CIN ≤12 months; present or planned participation in a HPV therapy trial
Mahmud 2012[30]	Kinshasa (Congo)	2003-2004	Prospective study	Population-based screening	1528/1571 (128)	30- (ND)	Pregnancy; hysterectomy
Sangrajrang 2017;[31] Sangrajrang 2019[32]	Ubon Ratchathani (Thailand)	2014-2015	Prospective study	Population-based routine screening	5046/ND (ND)	30-60 (ND)	ND
Kurokawa 2018[33] (FCCS study)	Fukui (Japan)	2015-2016	Prospective study	Population-based screening	7585/7585 (5284)	25-69 (ND)	Pregnant; no intact uterus; refusal of colposcopy and biopsy if required; previous (<12 months) treatment; previous (<12 months) CIN
Colposcopy-based studies							

First author and publication year (Study name)	Study location (country)	Enrollment year	Design	Setting	Sample size, assessed/eligible (potentially eligible non-participants)	Target age (average)	Exclusion criteria
Blumenthal 2001[34]	Harare (Zimbabwe)	1995-1997	Prospective study	Population-based screening	2073§/2199 (ND)	25-55 (ND)	Hysterectomy; previous cervical cancer
Coste 2003;[35] de Cremoux 2003[36]	Paris and adjacent cities (France)	1999-2000	Prospective study	Screening	1324‖/1757 (ND)	18- (35)	Pregnancy; surgery or laser therapy ≤1 year; visible cervix
Sankaranarayanan 2004[37]	Kolkata, Mumbai, and Trivandrum (India)	1999-2003	Prospective study	Population-based screening*	18085/20053 (ND)	25-65 (38)	ND
Qiao 2008[38] (START 2007)	Shanxi province (China)	2007	Prospective study	Population-based screening	2388/2530 (1191)	30-54 (43)	Pregnancy, previous cervical cancer, current menstruation
McAdam 2010[39]	Efate island (Vanuatu)	2006	Prospective study	Population-based screening	494/499¶ (ND)	30-50 (39)	Current evident cancer
Quincy 2012[40]	Leon (Nicaragua)	ND	Prospective study	Population-based screening	245/245 (ND)	25-60 (39)	Pregnancy, hysterectomy
Colposcopy-based correction studies							
Cuzick 2003[41] (HART study)	Birmingham, Edinburgh, London, Manchester, and Mansfield (UK)	1998-2001	Prospective study	Routine screening	10358/10868 (ND)	30-60 (42)	Abnormal smear ≤3 years; previous therapy for CIN
Petry 2003[42]	Hannover, Tubingen, and their adjacent areas (Germany)	1998-2000	Prospective study	Routine screening	7908/8101 (ND)	30- (ND)**	Hysterectomy; previous atypical cytology, CIN, or therapy for cervical disease ≤1 year; pregnant

First author and publication year (Study name)	Study location (country)	Enrollment year	Design	Setting	Sample size, assessed/eligible (potentially eligible non-participants)	Target age (average)	Exclusion criteria
Gravitt 2010[43] (CATCH study)	Medchal Mandal (India)	2005-2007	Prospective population-based study	Population-based screening*	2331/2331 (3272)	25- (37)	No intact uterus; mentally incompetent
Moy 2010[44] (START Project)	Shanxi, Jiangxi, and Gansu provinces (China)	2003-2006	Prospective population-based study	First-time screening	9057/9057 (ND††)	30-54 (39)	Previous cervical cancer screening; Pregnancy; history of CIN or cancer; hysterectomy; “debilitating” disease
Monsonego 2011[45] (FASE study)	Paris (France)	2008-2009	Prospective study	Routine opportunistic screening	4429 /4481 (525)	20-65 (~40)	Total hysterectomy; pregnancy; abnormal cytology ≤6 months
Ferreccio 2013[46]	Santiago (Chile)	2009-2010	Prospective study	Routine screening	8265/8309 (55)	25-64 (42)	Pregnancy; hysterectomy; virginity
Agorastos 2015[47]	Nine cities (Greece)	2011-2013	Prospective	Routine screening	3993/4009 (ND)	25-55 (40)	Pregnancy; previous CIN ≤5 years; follow-up for abnormal cytology; hysterectomy
Iftner 2015[48]	Tübingen, Saarbrücken, and Freiburg (Germany)	ND	Prospective study	Routine screening	9451/9859 (ND)	30-60 (ND)	Hysterectomy; destructive therapy of the cervix; pregnancy; abnormal cytology results ≤6 months; HIV infection; organ transplantation
Wu 2017[49]	Zhejiang (China)	2015	Prospective	Population-based screening	11064/11109 (ND‡‡)	21-65 (ND)	Pregnancy; ≤6 weeks post-partum; hysterectomy; previous CIN, vulvar or vaginal intraepithelial neoplasia+, previous malignancy, cervical cancer screening or

First author and publication year (Study name)	Study location (country)	Enrollment year	Design	Setting	Sample size, assessed/eligible (potentially eligible non-participants)	Target age (average)	Exclusion criteria
							therapy ≤3 years; already or planned vaccination against HPV

*Studies focused on subjects who had never participated in cervical cancer screening.

†Subjects assessed in the "diagnosis setting" group (i.e., those with an abnormal smear result enrolled for the evaluation of CIN) were excluded.

‡Reported participation rates in local areas ranged between 56 and 72%.

§Analysis was restricted to subjects for whom the data on all three screening tests (cytology, VIA, and HPV test) were available.

||Analysis was restricted to subjects who attended routine screening and for whom data on both cytology and HPV tests were available (second-phase of the study).

¶Analysis was restricted to subjects who attended routine screening and in whom four screening tests (cytology, VIA/VILI, colposcopy and HPV test) were performed (study 1). Subjects who participated in HPV screening alone (study 2) were excluded.

**The age ranged between 30 and 60 years in 94% of the subjects.

††Eligibility was assessed in 11424 subjects.

‡‡Approximately 20% of targeted women did not participate in screening.

ATHENA = Addressing THE Need for Advanced HPV diagnosis; CCCaST = Canadian cervical cancer screening trial; CIN = cervical intraepithelial neoplasia; FASE = French APTIMA screening evaluation; FCCS = Fukui Cervical Cancer Screening; GYN = gynecology; HIV = human immunodeficiency virus; RCT = randomized controlled trial; START = Screening Technologies to Advanced Rapid Testing; STD = sexually transmitted disease; VIA = visual inspection with acetic acid; VILI = visual inspection with Lugol's iodine

Table S2. Test Characteristics

First author and publication year (Study name)	Cytology method (LBC platform)	Cutoff for positive results (classification system)	Sample collectors	Collection devices	Assessors (external reviewers [algorithm for external review])	HPV assay (no. of genotypes)	HPV genotypes analyzed	Cutoff for positive results	Unsatisfactory or missing results (Pap / HPV, %)	Other screening modalities
Histology-based studies										
Belinson 2001;[18] Pan 2003;[19] Zhao 2010[20] (SPOCCS I)	LBC (ThinPrep)	ASCUS; LSIL; HSIL (BTS 1991)	Gynecologic oncologists	Spatula and endocervical brush	A local cytopathologist (a cytopathologist [all abnormal results; 5% of all normal results])	HC2 (13 high-risk types)	High-risk: 16, 18, 31, 33, 35, 39, 45, 51, 52, 56, 58, 59, 68	≥1 RLU	Considered as negative (0.2 / 2.9)	Self-collected HPV test; fluorescence spectroscopy; VIA
Cárdenas-Turanzas 2008[21]	CC (NA)	ASCUS (ND)	ND	ND	ND	HC2 (13 high-and 5 low-risk types)	High-risk: 16, 18, 31, 33, 35, 39, 45, 51, 52, 56, 58, 59, 68; low risk: 6, 11, 42, 43, 44	≥1 RLU for high-risk genotypes	Excluded (2.0 / 1.0)	Fluorescence and reflectance spectroscopy
Hovland 2010[22]	CC (NA); LBC (ThinPrep)	ASCUS; LSIL; HSIL (BTS 2001)	Gynecologists or general practitioners who participated in	Cervix brush	An experienced cytologist and 1 cytophathologist for positive results only (ND)	HPV DNA (24 high- and 21 low-risk); NASBRA	High-risk: 16, 18, 26, 30, 31, 33, 34, 35, 39, 45, 51, 52, 53, 56, 58, 59, 64, 66, 67, 68, 69, 73,	DNA assay: positive for 14 specific "high-risk" HPV genotypes (16,	Excluded (2.6 (CC), 3.8 (LBC) / 0)	None

First author and publication year (Study name)	Cytology method (LBC platform)	Cutoff for positive results (classification system)	Sample collectors	Collection devices	Assessors (external reviewers [algorithm for external review])	HPV assay (no. of genotypes)	HPV genotypes analyzed	Cutoff for positive results	Unsatisfactory or missing results (Pap / HPV, %)	Other screening modalities
			a two-week training			mRNA (5 for Pretect HPV-Proofer; 4 for a set of specific primers); RLB-based genotyping	82 (2 variants); low-risk: 40, 42, 43, 44, 54, 55, 57, 61, 70, 71, 72, 81, 83, 84, 85, 86, CP6108, JC9710; Pretect HPV-Proofer: 16, 18, 31, 33, 45; a set of primers: 35, 51, 52, 58	18, 31, 33, 35, 39, 45, 51, 52, 56, 58, 59, 68); NASBRA: positive for 9 HPV genotypes or Pretect HPV-Proofer only		
Histology-based correction studies										
Schneider 2000[23]	CC (NA)	III (SMCC) *	Gynecologists	Cytobrush	ND	A customized PCR with a cocktail probe and genotyping	High-risk: 16, 18, 31, 33, 35, 39, 45, 51, 52, 56, 58, 59, 66,68; low risk: 6, 11, 40, 42, 43, 44	0.5 to 10 fg/DNA for high-risk group	Excluded (0.6 / 0.9)	"Screening" colposcopy (without biopsy)

First author and publication year (Study name)	Cytology method (LBC platform)	Cutoff for positive results (classification system)	Sample collectors	Collection devices	Assessors (external reviewers [algorithm for external review])	HPV assay (no. of genotypes)	HPV genotypes analyzed	Cutoff for positive results	Unsatisfactory or missing results (Pap / HPV, %)	Other screening modalities
						analyzed for 14 high- and 6 low-risk types				
Kulasingam 2002;[24] Balasurbramanian 2010[25]	LBC (ThinPrep)	ASCUS (BTS 1991)	Gynecologists	Cytobrush and spatula	A cytotechnologist and review by a pathologist; 10% of negative results reviewed (ND)	A customized PCR with genotyping (13 high-risk and 5 additional types) or single amplification (13)	16, 18, (26,) 31, 33, 35, 39, 45, 51, 52, (55,) 56, 58, 59, 68, (73, 82, 84)	≥1 RLU	Considered as positive (1.9 / 3.9)	None
Bigras 2005[26]	LBC (SurePath)	ASCUS (BTS 2001)	Gynecologists	Cervex brush	One of 13 cytotechnicians; all samples associated with	HC2 (13 high-risk types)	16, 18, 31, 33, 35, 39, 45, 51, 52, 56, 58, 59, 68	≥1 RLU	ND	None

First author and publication year (Study name)	Cytology method (LBC platform)	Cutoff for positive results (classification system)	Sample collectors	Collection devices	Assessors (external reviewers [algorithm for external review])	HPV assay (no. of genotypes)	HPV genotypes analyzed	Cutoff for positive results	Unsatisfactory or missing results (Pap / HPV, %)	Other screening modalities
					subsequent CIN2+ were reviewed (ND)					
Mayrand 2007[27] (CCCaST)	CC (NA)	ASCUS (BTS 2001)	ND	ND	ND	HC2 (13 high-risk types)	16, 18, 31, 33, 35, 39, 45, 51, 52, 56, 58, 59, 68	≥1 RLU	Excluded (1.5 / 0.4)	None
Li 2009[28] (IARC 1-3)	LBC (ThinPrep)	ASCUS (BTS 2001)	Gynecologists	Cytobrush	All abnormal and 10% of normal results were verified by a second experienced cytologist (ND)	HC2 (13 high-risk types)	16, 18, 31, 33, 35, 39, 45, 51, 52, 56, 58, 59, 68	≥1 RLU	ND	Colposcopy, VIA, VILI, and fluorescence spectoroscopy
Castle 2011[29] (ATHENA study)	LBC (ThinPrep)	ASCUS (BTS 2001)	ND	Spatula and endocervix brush	ND	Cobas HPV (2 [out of 14] high-risk types) (Amplicor HPV; Linear	16, 18 (31, 33, 35, 39, 45, 51, 52, 56, 58, 59, 66, 68)	Only 16 and/or 18 positive	Excluded (2.2 / 0.3)	None

First author and publication year (Study name)	Cytology method (LBC platform)	Cutoff for positive results (classification system)	Sample collectors	Collection devices	Assessors (external reviewers [algorithm for external review])	HPV assay (no. of genotypes)	HPV genotypes analyzed	Cutoff for positive results	Unsatisfactory or missing results (Pap / HPV, %)	Other screening modalities
						Array HPV genotyping)				
Mahmud 2012[30]	CC (NA)	ASCUS; LSIL; HSIL (BTS 2001)	Trained research nurses	Cytobrush	A blinded cytology technician and a blinded cytopathologist (ND)	HC2 (13 high-risk types); an expanded probe set (4 types)	HC2: 16, 18, 31, 33, 35, 39, 45, 51, 52, 56, 58, 59, 68; an expanded set: 26, 66, 73, 82	≥1 RLU for HC2 only and HC2 plus an expanded set	Excluded (9.3 / 11.5)	Colposcopy, VIA, and VILI
Sangrajrang 2017;[31] Sangrajrang 2019[32]	CC (NA)	ASCUS (BTS)	Trained nurses	Spatula and endocervical brush	A cytotechnician screener and a cytopathologist (ND)	Cobas HPV (14 high-risk types); Aptima HPV (14 high-risk types)	16, 18, 31, 33, 35, 39, 45, 51, 52, 56, 58, 59, 66, 68	Any high-risk HPV positive; only 16/18 positive (Cobas); or 16/18/45 (Aptima)	Unclear (5.1)	None
Kurokawa 2018[33] (FCCS study)	LBC (ThinPrep)	ASCUS (BTS2001)	Gynecologists	Endocervical brush with bloom-like device	Blinded cytologists at one accredited center (ND)	Cobas HPV (14 high-risk types)	16, 18, 31, 33, 35, 39, 45, 51, 52, 56, 58, 59, 66, 68	Any high-risk HPV positive; or only 16 and/or 18	ND	None

First author and publication year (Study name)	Cytology method (LBC platform)	Cutoff for positive results (classification system)	Sample collectors	Collection devices	Assessors (external reviewers [algorithm for external review])	HPV assay (no. of genotypes)	HPV genotypes analyzed	Cutoff for positive results	Unsatisfactory or missing results (Pap / HPV, %)	Other screening modalities
								positive		
Colposcopy-based studies										
Blumenthal 2001[34]	CC (NA)	LSIL (ND)	Trained nurse midwives	Spatula	ND	HC2 (13 high-risk types)	16, 18, 31, 33, 35, 39, 45, 51, 52, 56, 58, 59, 68	≥1 RLU	Excluded (ND)	VIA
Coste 2003;[35] de Cremoux 2003[36]	CC (NA); LBC (ThinPrep)	ASCUS; LSIL; HSIL (BTS 1991)	Skilled gynecologists	Cervex brush or "appropriate" brush and spatula	ND	HC2 (13 high and 5 low-risk types)	High-risk: 16, 18, 31, 33, 35, 39, 45, 51, 52, 56, 58, 59, 68; low risk: 6, 11, 42, 43, 44	≥1 RLU for both low- and high-risk; and high-risk genotypes only	Excluded (ND)	None
Sankaranarayanan 2004[37]	CC (NA)	LSIL (ND)	Five high school graduates; a registered nurse; 3 cytotechnicians; 6 university graduates who	Cytobrush alone or spatula and cotton swab	ND	HC2 (13 high-risk types)	16, 18, 31, 33, 35, 39, 45, 51, 52, 56, 58, 59, 68	≥1 RLU	ND	VIA and VILI

First author and publication year (Study name)	Cytology method (LBC platform)	Cutoff for positive results (classification system)	Sample collectors	Collection devices	Assessors (external reviewers [algorithm for external review])	HPV assay (no. of genotypes)	HPV genotypes analyzed	Cutoff for positive results	Unsatisfactory or missing results (Pap / HPV, %)	Other screening modalities
			were trained for collecting samples							
Qiao 2008[38] (START 2007)	LBC (SurePath)	ASC-H (BTS 2001)	Nurse midwives	Cervical brush	ND	HC2 (13 high-risk types); CareHPV (14 high-risk types)	HC2:16, 18, 31, 33, 35, 39, 45, 51, 52, 56, 58, 59, 68; CareHPV: 16, 18, 31, 33, 35, 39, 45, 51, 52, 56, 58, 59, 66, 68	≥1 RLU for HC2; ≥0.5 RLU for CareHPV	Excluded (5.6; all screening tests jointly analyzed)	VIA
McAdam 2010[39]	CC (NA)	LSIL; HSIL (ND)	Nurses	Cytobrush and Cervex sampler	Accredited pathology providers (ND)	HC2 (13 high-risk types)	16, 18, 31, 33, 35, 39, 45, 51, 52, 56, 58, 59, 68	ND	Excluded (3.0 / 0.6)	VIA and VILI
Quincy 2012[40]	LBC (ND)	ASC-H (ND)	Four clinicians who participated in a refresher training	Cytobrush and spatula	Two experienced pathologists (ND)	HC2 (13 high-risk types)	16, 18, 31, 33, 35, 39, 45, 51, 52, 56, 58, 59, 68	ND	ND	None
Colposcopy-based										

First author and publication year (Study name)	Cytology method (LBC platform)	Cutoff for positive results (classification system)	Sample collectors	Collection devices	Assessors (external reviewers [algorithm for external review])	HPV assay (no. of genotypes)	HPV genotypes analyzed	Cutoff for positive results	Unsatisfactory or missing results (Pap / HPV, %)	Other screening modalities
correction studies										
Cuzick 2003[41] (HART study)	CC (NA)	Mild dyskaryosis; borderline change (BSCC 1986)†	ND	Spatula and cervix brush	ND (no external review [NA])	HC2 (13 high-risk types)	16, 18, 31, 33, 35, 39, 45, 51, 52, 56, 58, 59, 68	≥1 RLU	Excluded (1.4 / ND)	None
Petry 2003[42]	CC (NA)	IIw (SMCC) ‡	ND	Cotton swab	Practice-based laboratories (ND [all abnormal cytological results, HPV+ samples, and randomly selected 5% of all negative samples])	HC2 (13 high-risk types)	16, 18, 31, 33, 35, 39, 45, 51, 52, 56, 58, 59, 68	≥1 RLU	Considered as positive in cytology (ND / ND)	None
Gravitt 2010[43] (CATCH study)	CC (NA)	ASCUS (BTS 2001)	Gynecologists	Spatula	A trained local cytopathologist only (ND)	HC2 (13 high-risk types)	16, 18, 31, 33, 35, 39, 45, 51, 52, 56, 58, 59, 68	≥1 RLU	Considered as negative (3.8 / ND)	VIA

First author and publication year (Study name)	Cytology method (LBC platform)	Cutoff for positive results (classification system)	Sample collectors	Collection devices	Assessors (external reviewers [algorithm for external review])	HPV assay (no. of genotypes)	HPV genotypes analyzed	Cutoff for positive results	Unsatisfactory or missing results (Pap / HPV, %)	Other screening modalities
Moy 2010[44] (START Project)	LBC (ThinPrep or AutoCyte)	ASCUS (BTS 1991)	Gynecologists	Cytobrush	Multiple general or gynecology cytopathologists; confirmation by a senior cytopathologist in case of discrepant results (an international expert [ND])	HC2 (13 high-risk types)	16, 18, 31, 33, 35, 39, 45, 51, 52, 56, 58, 59, 68	≥1 RLU	Considered as positive (1.9 / 6.0)	VIA, VILI, coloposcopy (years 2004 and 2006 only)
Monsonego 2011[45] (FASE study)	LBC (ThinPrep)	ASCUS (BTS 2001)	Gynecologists	Cervex brush	Multiple cytopathologists(a reviewer [all abnormal and 10% of normal results])	HC2 (13 high-risk types); Aptima HPV (14 high-risk types)	16, 18, 31, 33, 35, 39, 45, 51, 52, 56, 58, 59, (66,) 68	≥1 RLU ; ≥1 SCO	Excluded (10.5 / ND)	None
Ferreccio 2013[46]	CC (NA)	ASCUS (BTS 2001)	Midwives	Spatula and cotton swab	Multiple cytotechnicians; quality controlled	HC2 (13 high-risk types)	16, 18, 31, 33, 35, 39, 45, 51, 52, 56, 58, 59, 68	≥1 RLU	Considered as negative if not repeated (0.4 /	VIA

First author and publication year (Study name)	Cytology method (LBC platform)	Cutoff for positive results (classification system)	Sample collectors	Collection devices	Assessors (external reviewers [algorithm for external review])	HPV assay (no. of genotypes)	HPV genotypes analyzed	Cutoff for positive results	Unsatisfactory or missing results (Pap / HPV, %)	Other screening modalities
					by multiple pathologists (ND)				ND)	
Agorastos 2015[47]	LBC (ThinPrep)	ASCUS (BTS 2001)	ND	Cervex brush	Local cytologists per usual practice	Cobas HPV (14 high-risk types)	16, 18, 31, 33, 35, 39, 45, 51, 52, 56, 58, 59, 66, 68	Any high-risk HPV positive	Excluded (0.4; both methods jointly assessed)	None
Iftner 2015[48]	LBC (ThinPrep)	III (SMCC) *	ND	Cervex brush	ND	HC2 (13 high-risk types); Aptima HPV (14 high-risk types); INNO-LiPA HPV Genotyping Extra	16, 18, 31, 33, 35, 39, 45, 51, 52, 56, 58, 59, (66,) 68	≥1 RLU ; ≥1 SCO; 16 only or 16 and/or 18 positive	ND	None
Wu 2017[49]	LBC (ThinPrep)	ASCUS (BTS 2014)	Gynecologists	Cytobrush	Cytologists screened with/without	Cobas HPV (14 high-risk types)	16, 18, 31, 33, 35, 39, 45, 51, 52, 56, 58, 59, 66, 68	Any high-risk HPV positive; or only 16	Excluded (0.4; both methods jointly	None

First author and publication year (Study name)	Cytology method (LBC platform)	Cutoff for positive results (classification system)	Sample collectors	Collection devices	Assessors (external reviewers [algorithm for external review])	HPV assay (no. of genotypes)	HPV genotypes analyzed	Cutoff for positive results	Unsatisfactory or missing results (Pap / HPV, %)	Other screening modalities
					computer-aided reading, confirmed by two pathologists and a third pathologist in case of discordant results (ND)			and/or 18 positive	assessed)	

*Category III by the SMCC classification system was considered as LSIL in BTS 2001.

†Categories of mild dyskaryosis and borderline change by the BSCC 1986 classification system were respectively considered as LSIL and ASCUS in BTS 2001.

‡Category IIw by the SMCC classification system was considered as ASCUS in BTS 2001.

ASCUS = atypical squamous cells of undetermined significance; BSCC = British Society for Clinical Cytology; CC = conventional cytology; BTS = Bethesda system; HC2 = Hybrid Capture 2; HPV = human papilloma virus; HR = high-risk; LBC = liquid-based cytology; NA = not applicable; ND = no data; RLU = relative light unit; SCO = signal cutoff ratio; SMCC = Second Munich cytological classification; VIA; VILI

Table S3. Reference Standard Characteristics

First author and publication year (Study name)	Primary (secondary) outcome of interest	Reference standards	Blinding of screening results to participants / colposcopists / pathologists	Algorithm for submission to reference standards	Percentage of planned/completed histological verification [colposcopic verification]				Total proportion of histological verification, %	Analytical operationalizations
					Cytology+ and HPV+	Cytology+ and HPV-	Cytology- and HPV+	Cytology- and HPV-		
Histology-based studies										
Belinson 2001;[18] Pan 2003;[19] Zhao 2010[20] (SPOCCS I)	CIN2+ (CIN3+; cancer)	Biopsy for visually abnormal lesions or standardized biopsy for visually normal sites; and routine ECC for all subjects	Blinded / blinded / blinded	All received reference standards	NA/100	NA/100	NA/100	NA/100	100	Missing results (0.3%) considered as negative; sensitivity analysis performed but specific methods not reported
Cárdenas-Turanzas 2008[21]	CIN2+	Biopsy for visually abnormal lesions and standardized biopsy for visually normal sites for all subjects	Blinded / blinded / blinded	All received reference standards	NA/100	NA/100	NA/100	NA/100	100	Missing results (1.3%) excluded
Hovland 2010[22]	CIN2+	Biopsy for visually abnormal lesions or standardized biopsy for visually normal sites	Blinded / blinded / blinded	All received reference standards	NA/100	NA/100	NA/100	NA/100	100	Missing results (8.7%) excluded
Histology-based correction studies										
Schneider 2000[23]	CIN2+	- Biopsy for visually abnormal lesions or standardized biopsy for	Not blinded / not blinded / blinded	All with ≥1 positive screening test	100 / 74 (any positive	100 / 74 (any positive	100 / 74 (any positive	100 / 74 (any positive jointly	8	IPW correction by Begg et al. 1983;[4] deterministic

First author and publication year (Study name)	Primary (secondary) outcome of interest	Reference standards	Blinding of screening results to participants / colposcopists / pathologists	Algorithm for submission to reference standards	Percentage of planned/completed histological verification [colposcopic verification]				Total proportion of histological verification, %	Analytical operationalizations
					Cytology+ and HPV+	Cytology+ and HPV-	Cytology- and HPV+	Cytology- and HPV-		
		visually normal sites plus ECC or ECC only for algorithmically selected subjects; histology from conization also included<br>- Biopsy performed for an unreported portion of subjects based on follow-up tests		(cytology; HPV; or “screening colposcopy”)	jointly estimated)	jointly estimated)	jointly estimated)	estimated; for “screening colposcopy” positive); 0 / 1 (only a portion)		sensitivity analyses performed for missing results
Kulasingam 2002;[24] Balasurbramanian 2010[25]	CIN3+ (CIN2+)	Biopsy for visually abnormal lesions or standardized biopsy for visually normal sites; ECC for selected cases for algorithmically selected subjects	ND / ND / blinded	All with ≥1 positive screening test	100 / 78	100 / 58	100 / 44	Randomly selected 45 (of the first 1000 subjects) / 8	25	IPW correction;[24] sensitivity analyses performed for missing and inadequate results
Bigras 2005[26]	CIN2+	Biopsy for visually abnormal lesions or standardized biopsy for visually normal sites and additional histological evaluation (paraffin block	ND / ND / ND	All with ≥1 positive screening test	100 / 79	100 / 65	100 / 79	Randomly selected 6 (700 subjects) / 4	7	IPW correction by Ratnam et al. 2000[50]

First author and publication year (Study name)	Primary (secondary) outcome of interest	Reference standards	Blinding of screening results to participants / colposcopists / pathologists	Algorithm for submission to reference standards	Percentage of planned/completed histological verification [colposcopic verification]				Total proportion of histological verification, %	Analytical operationalizations
					Cytology+ and HPV+	Cytology+ and HPV-	Cytology- and HPV+	Cytology- and HPV-		
		preparation from sample from the collecting device) for algorithmically selected subjects								
Mayrand 2007[27] (CCCaST)	CIN2+ (CIN3+)	- Biopsy for visually abnormal lesions and standardized biopsy for visually normal sites plus ECC for algorithmically selected subjects<br>- LEEP or conization in selected subjects for treatment	ND / blinded / blinded	All with ≥1 positive screening test	100 / 99	100 / 88	100 / 91	ND / randomly selected 7	13	IPW correction by Kulasingam et al. 2002[24] and Ratnam et al. 2000[50]
Li 2009[28] (IARC 1-3)	CIN2+	- Biopsy for visually abnormal lesions for subjects with positive "screening" colposcopy, VIA, or fluorescence spectroscopy<br>- Biopsy for visually abnormal lesions and	Partly blinded / partly blinded / ND	"Screening" colposcopy and fluorescence spectroscopy positive; all with LSIL+ or	100 /ND	100 /ND	0 / ND	100 / ND ("screening" fluorescence spectroscopy positive); 100 / ND ("screening"	25	IPW correction by Almonte et al. 2007[51]

First author and publication year (Study name)	Primary (secondary) outcome of interest	Reference standards	Blinding of screening results to participants / colposcopists / pathologists	Algorithm for submission to reference standards	Percentage of planned/completed histological verification [colposcopic verification]				Total proportion of histological verification, %	Analytical operationalizations
					Cytology+ and HPV+	Cytology+ and HPV-	Cytology- and HPV+	Cytology- and HPV-		
		standardized biopsy for visually normal sites or ECC (in the case SCJ not visible) for algorithmically selected subjects		HPV+/ASCUS				colposcopy or VIA); 0 / ND		
Castle 2011[29] (ATHENA study)	CIN2+ planned but changed to CIN3+	Biopsy for visually abnormal lesions and standardized biopsy for visually normal sites or ECC (in the case of unsatisfactory colposcopy) for all algorithmically selected subjects	Blinded / blinded / blinded	All with ≥1 positive for cytology or first-generation HPV test (not on Cobas HPV)	ND / 86	ND / 84	ND / 83	ND / 9	19	Correction method by Zhou et al. 2002[52]
Mahmud 2012[30]	CIN2+; CIN3+	Biopsy for visually abnormal lesions; and standardized biopsy for visually normal sites in randomly selected 20% of normal colposcopy results in "screening" colposcopy for all subjects	Blinded / blinded (but not to VIA/VILI)/ blinded	All received colposcopy as a screening test	NA / ND (63 for cyto-; 47 for cyto+; 65 for HPV-; 51 for HPV+)	NA /ND (63 for cyto-; 47 for cyto+; 65 for HPV-; 51 for HPV+)	NA / ND (63 for cyto-; 47 for cyto+; 65 for HPV-; 51 for HPV+)	NA /ND (63 for cyto-; 47 for cyto+; 65 for HPV-; 51 for HPV+)	40	IPW correction by Choi 1992[53] and Ratnam et al. 2000[50]

First author and publication year (Study name)	Primary (secondary) outcome of interest	Reference standards	Blinding of screening results to participants / colposcopists / pathologists	Algorithm for submission to reference standards	Percentage of planned/completed histological verification [colposcopic verification]				Total proportion of histological verification, %	Analytical operationalizations
					Cytology+ and HPV+	Cytology+ and HPV-	Cytology- and HPV+	Cytology- and HPV-		
Sangrajrang 2017;[31] Sangrajrang 2019[32]	CIN2+	Biopsy for visually abnormal lesions or random biopsy for women with normal colposcopy (colposcopy and histological results obtained in 94-95% of all women with)	Not blinded / ND / ND	All with ≥1 positive screening test	100 / 94-95 (any positive jointly estimated)	100 / 94-95 (any positive jointly estimated)	100 / 94-95 (any positive jointly estimated)	0 / 0	6.7	Bayesian LCM (conditional independence model) by Menten et al. 2008[54]
Kurokawa 2018[33] (FCCS study)	CIN2+	Biopsy for visually abnormal lesions or random biopsy at the JCJ for women with normal colposcopy	Not blinded / ND / blinded	All with ≥1 positive screening test	100 / 64	100 / 35	100 / 60	ND / 0.2	5	IPW according to the ATHENA study[29]
Colposcopy-based studies										
Blumenthal 2001[34]	CIN2+	Screening colposcopy for all; colposcopy-directed biopsy for visually abnormal lesions only;	Blinded / blinded / blinded	All received colposcopy as a screening test	[100/100]	[100/100]	[100/100]	[100/100]	ND	Missing or inadequate results excluded
Coste 2003;[35] de	CIN2+	Screening colposcopy for all;	Blinded /	All received	[100/100]	[100/100]	[100/100]	[100/100]	ND	ND

First author and publication year (Study name)	Primary (secondary) outcome of interest	Reference standards	Blinding of screening results to participants / colposcopists / pathologists	Algorithm for submission to reference standards	Percentage of planned/completed histological verification [colposcopic verification]				Total proportion of histological verification, %	Analytical operationalizations
					Cytology+ and HPV+	Cytology+ and HPV-	Cytology- and HPV+	Cytology- and HPV-		
Cremoux 2003[36]	(CIN1+)	colposcopy-directed biopsy for visually abnormal lesions only	blinded / blinded	colposcopy as a screening test						
Sankaranarayanan 2004[37]	CIN2+	Colposcopy for all; colposcopy-directed biopsy for visually abnormal lesions only	Blinded / blinded / blinded	All received colposcopy as a screening test	[100/100]	[100/100]	[100/100]	[100/100]	17	Inconclusive (1466 cases (7.3%)) and missing (due to refusal of biopsy, 502 cases (2.5%)) results were excluded
Qiao 2008[38] (START 2007)	CIN2+	- Screening colposcopy for all; colposcopy-directed biopsy for visually abnormal lesions only in screening colposcopy<br>- Standardized biopsy for visually normal sites and ECC for subjects with an abnormal/unsatisfactory cytology or positive HPV	Partly blinded / partly blinded / blinded	All received colposcopy as a screening test; those with ≥1 positive or unsatisfactory cytology received routine biopsy	[100/100]	[100/100]	[100/100]	[100/100]	ND	ND

First author and publication year (Study name)	Primary (secondary) outcome of interest	Reference standards	Blinding of screening results to participants / colposcopists / pathologists	Algorithm for submission to reference standards	Percentage of planned/completed histological verification [colposcopic verification]				Total proportion of histological verification, %	Analytical operationalizations
					Cytology+ and HPV+	Cytology+ and HPV-	Cytology- and HPV+	Cytology- and HPV-		
McAdam 2010[39]	CIN2+	Screening colposcopy for all; biopsy for visually abnormal lesions or standardized biopsy for subjects with positive cytology; histology from LEEP also included	Partly blinded / partly blinded / ND	All received colposcopy as a screening test; those with positive cytology received biopsy	100 / 90 [100 / 100]	100 / 90 [100 / 100]	NA / ND [100 / 100]	NA / ND [100 / 100]	42	ND
Quincy 2012[40]	CIN2+	Screening colposcopy for all; colposcopy-directed biopsy for visually abnormal lesions or ECC for subjects with invisible transformation zone	Blinded / blinded / ND	All received colposcopy as a screening test	100 / ND [100 / 100]	100 / ND [100 / 100]	100 / ND [100 / 100]	100 / ND [100 / 100]	ND	ND
Colposcopy-based correction studies										
Cuzick 2003[41] (HART study)	CIN2+	Colposcopy for algorithmically selected subjects; biopsy for visually abnormal lesions only	ND / not blinded / blinded	Immediate colposcopy if ≥LSIL; otherwise	[100 / 70 (jointly estimated)]	[100 / 70 (jointly estimated)]	[100 / 100 (jointly estimated)]	[Randomly selected 5 / 5]	11	IPW correction[41]

First author and publication year (Study name)	Primary (secondary) outcome of interest	Reference standards	Blinding of screening results to participants / colposcopists / pathologists	Algorithm for submission to reference standards	Percentage of planned/completed histological verification [colposcopic verification]				Total proportion of histological verification, %	Analytical operationalizations
					Cytology+ and HPV+	Cytology+ and HPV-	Cytology- and HPV+	Cytology- and HPV-		
				randomly assigned to either immediate or late colposcopy						
Petry 2003[42]	CIN2+ (CIN3+)	Colposcopy for algorithmically selected subjects; biopsy for visually abnormal lesions only	ND / ND / blinded for external reviewers	All with ≥1 positive screening test	[100 / 75 (jointly estimated)]	[100 / 75 (jointly estimated)]	[100 / 75 (jointly estimated)]	[Randomly selected 5 / 3]	5	IPW correction for subjects with a double-negative; exclusion of subjects with ≥1 positive who refused colposcopy[42]
Gravitt 2010[43] (CATCH study)	ND (CIN2+ or CIN3+)	Colposcopy for algorithmically selected subjects; biopsy for visually abnormal lesions only	ND / aware of ≥1 positive / ND	All with ≥1 positive result for cytology, HPV test, or VIA	[100 / 65 (jointly estimated; 38 if limited to successfully evaluated cases)]	[100 / 65 (jointly estimated; 38 if limited to successfully evaluated cases)]	[100 / 65 (jointly estimated; 38 if limited to successfully evaluated cases)]	[Randomly selected ND / 19]	7	IPW correction allowing for 3 tests[43]

First author and publication year (Study name)	Primary (secondary) outcome of interest	Reference standards	Blinding of screening results to participants / colposcopists / pathologists	Algorithm for submission to reference standards	Percentage of planned/completed histological verification [colposcopic verification]				Total proportion of histological verification, %	Analytical operationalizations
					Cytology+ and HPV+	Cytology+ and HPV-	Cytology- and HPV+	Cytology- and HPV-		
Moy 2010[44] (START Project)	CIN3+ (CIN2+)	Colposcopy for subjects with ≥1 screening abnormality or all (2 separate whole years only); biopsy for visually abnormal sites or standardized sites for ≥1 positive cytology or HPV test only; histology from LEEP or conization for cases with CIN2+ also included	ND / ND / ND	All with VIA/VILI+ regardless of other results or ≥1 positive for LSIL+ or HPV+ in 2003 and 2005; all with ≥1 positive in 2004 and 2006	[ND / 99 (95 for ASC)]	[ND / 81 (61 for ASC)]	[ND/ 89 (88 for ASC)]	[ND / 59 (59 for ASC)]	22	IPW correction by Kulasingam et al. 2002;[24] sensitivity analyses performed for inadequate, insufficient, and missing outcomes
Monsonego 2011[45] (FASE study)	ND (CIN2+ or CIN3+)	- Colposcopy for algorithmically selected subjects; biopsy for visually abnormal sites or standardize sites any 1 screening test positive<br>- LEEP or conization for selected cases	ND / ND / blinded only to HPV	All with ≥1 positive screening test	[100 / 92 (jointly estimated)]	[100 / 92 (jointly estimated)]	[100 / 92 (jointly estimated)]	[Randomly selected 14 of double-negatives / 14]	25	Correction by Zhou et al. 1998[55] and Roldan-Nofuentes et al. 2008[56]
Ferreccio 2013[46]	ND	Colposcopy for	Partly not	All with ≥1	[100 / 94	[100 / 94	[100 / 94	[Selected	ND	IPW correction by

First author and publication year (Study name)	Primary (secondary) outcome of interest	Reference standards	Blinding of screening results to participants / colposcopists / pathologists	Algorithm for submission to reference standards	Percentage of planned/completed histological verification [colposcopic verification]				Total proportion of histological verification, %	Analytical operationalizations
					Cytology+ and HPV+	Cytology+ and HPV-	Cytology- and HPV+	Cytology- and HPV-		
	(CIN2+ or CIN3+)	algorithmically selected subjects; biopsy for visually abnormal lesions only; histological results at treatment also included	blinded / blinded / blinded	positive screening test	(jointly estimated)]	(jointly estimated)]	(jointly estimated)]	based on cytology or high-risk profile / 4]		Katki et al. 2012[57]
Agorastos 2015[47]	CIN2+ (CIN3+)	- Colposcopy for algorithmically selected subjects; biopsy for visually abnormal lesions only; women with visually normal cervix were rescreened in 1 year<br>- LEEP or conization in selected subjects for treatment	ND / ND / blinded only to HPV	All with ≥1 positive screening test	[100 / 85]	[100 / 46]	[100 / 79]	[ND / Randomly selected 3 (106 subjects)]	5	IPW correction[†]
Iftner 2015[48]	ND (CIN2+ or CIN3+ (sensitivity only))	Colposcopy for algorithmically selected subjects; biopsy for visually abnormal lesions only	ND / not blinded / blinded (external histological	≥1 positive result for LBC; or any of HPV tests	[100 / 92]	[100 / 92]	[100 / 85]	[Randomly selected 5 (438 subjects) / 4]	6	IPW correction for ≥1 positive strata and an ad-hoc method for all negatives[58]

First author and publication year (Study name)	Primary (secondary) outcome of interest	Reference standards	Blinding of screening results to participants / colposcopists / pathologists review)	Algorithm for submission to reference standards	Percentage of planned/completed histological verification [colposcopic verification]				Total proportion of histological verification, %	Analytical operationalizations
					Cytology+ and HPV+	Cytology+ and HPV-	Cytology- and HPV+	Cytology- and HPV-		
Wu 2017[49]	CIN2+ (CIN3+)	Colposcopy for algorithmically selected subjects; biopsy for "high-grade lesion" by colposcopy; cytology LSIL+; cytology ASCUS and HPV+; HPV 16/18+; AGC; unsatisfactory colposcopy	ND / not blinded / blinded	All with ≥1 positive screening test	[100 / 93]	[100 / 93]	[100 / 95]	[Randomly selected 5 / 4]	ND	IPW correction[49]

*Studies that performed biopsy (and ECC for selected cases) simultaneously with colposcopy for all algorithmically selected subjects regardless of screening test results.

†Although this study randomly selected 3% of women who had tested negative to both screening tests for colposcopy and biopsy, no statistical corrections was performed. Data extracted from the full cross tabulation (Table 2 in the original article) were statistically corrected using inverse probability weighting by the review authors for this meta-analysis.

AGC; ASC; ASCUS; ECC = endocervical curettage; HPV; IPW = inverse probability weighting; LCM = latent-class model; LEEP; NS; NP = not performed; RS = reference standard; VIA = visual inspection with acetic acid; VILI = visual inspection with Lugol iodine

Table S4. Comparative Evidence of Cervical Cancer Screening Tests or Combination Algorithms.*

Index and comparator tests or combination algorithms		Comparator tests or combination algorithms																								
		PCR OR ≥ASCUS	HC2 OR ≥ASCUS	mRNA OR ≥ASCUS	PCR	HC2 OR ≥LSIL	HC2 OR ≥HSIL	HC2	mRNA	HPV16/18/45 OR [mRNA AND ≥ASCUS]	≥LSIL OR [PCR AND ASCUS]	HPV16/18 OR ≥ASCUS	≥LSIL OR [HC2 AND ASCUS]	HPV16/18 OR [PCR AND ≥ASCUS]	mRNA AND ≥ASCUS	≥HSIL OR [HC2 AND ≥ASCUS]	≥ASCUS	PCR AND ≥ASCUS	≥ASCH	HC2 AND ≥ASCUS	HPV16/18	≥LSIL	HC2 AND ≥LSIL	HPV16/18 AND ≥ASCUS	≥HSIL	HC2 AND ≥HSIL
Index tete too combination algorithms	PCR OR ≥ASCUS		0 (0)	0 (0)	3 (22642)	0 (0)	0 (0)	0 (0)	0 (0)	0 (0)	1 (11064)	0 (0)	0 (0)	2 (18649)	0 (0)	0 (0)	3 (22642)	3 (22642)	0 (0)	0 (0)	1 (7585)	1 (11064)	0 (0)	0 (0)	0 (0)	0 (0)
	HC2 OR ≥ASCUS			1 (4429)	0 (0)	3 (14272)	4 (16027)	10 (53303)	1 (4429)	0 (0)	0 (0)	0 (0)	3 (14917)	0 (0)	0 (0)	1 (1755)	10 (53303)	0 (0)	1 (7908)	7 (46687)	0 (0)	5 (18024)	2 (12920)	0 (0)	5 (18024)	3 (14675)
	mRNA OR ≥ASCUS				0 (0)	0 (0)	0 (0)	1 (4429)	1 (4429)	0 (0)	0 (0)	0 (0)	0 (0)	0 (0)	0 (0)	0 (0)	1 (4429)	0 (0)	0 (0)	0 (0)	0 (0)	0 (0)	0 (0)	0 (0)	0 (0)	0 (0)
	PCR					0 (0)	0 (0)	0 (0)	1 (5046)	1 (5046)	1 (11064)	0 (0)	0 (0)	3 (23695)	1 (5046)	0 (0)	5 (28001)	4 (27688)	0 (0)	0 (0)	2 (12631)	3 (16096)	0 (0)	0 (0)	1 (313)	0 (0)
	HC2 OR ≥LSIL						4 (23329)	5 (25402)	0 (0)	0 (0)	0 (0)	0 (0)	3 (21977)	0 (0)	0 (0)	0 (0)	4 (23329)	0 (0)	0 (0)	2 (12920)	0 (0)	4 (16345)	3 (14993)	0 (0)	3 (14272)	2 (12920)
	HC2 OR ≥HSIL							5 (25084)	0 (0)	0 (0)	0 (0)	0 (0)	3 (21977)	0 (0)	0 (0)	1 (1755)	5 (25084)	0 (0)	0 (0)	3 (14675)	0 (0)	4 (16027)	2 (12920)	0 (0)	4 (16027)	3 (14675)
	HC2								2 (13880)	0 (0)	0 (0)	0 (0)	5 (25639)	0 (0)	0 (0)	1 (1757)	14 (84298)	0 (0)	3 (10541)	7 (46689)	1 (9451)	8 (38675)	3 (14993)	0 (0)	6 (18517)	3 (14677)
	mRNA									1 (5046)	0 (0)	0 (0)	0 (0)	1 (5046)	1 (5046)	0 (0)	3 (18926)	1 (5046)	0 (0)	0 (0)	2 (14497)	0 (0)	0 (0)	0 (0)	0 (0)	0 (0)
	HPV16/18/45 OR [mRNA AND										0 (0)	0 (0)	0 (0)	1 (5046)	1 (5046)	0 (0)	1 (5046)	1 (5046)	0 (0)	0 (0)	1 (5046)	0 (0)	0 (0)	0 (0)	0 (0)	0 (0)

	≥ASCUS]																									
	≥LSIL OR [PCR AND ASCUS]											0 (0)	0 (0)	1 (11064)	0 (0)	0 (0)	1 (11064)	1 (11064)	0 (0)	0 (0)	0 (0)	1 (11064)	0 (0)	0 (0)	0 (0)	0 (0)
	HPV16/18 OR ≥ASCUS												0 (0)	0 (0)	0 (0)	0 (0)	1 (40901)	0 (0)	0 (0)	0 (0)	1 (40901)	0 (0)	0 (0)	1 (40901)	0 (0)	0 (0)
	≥LSIL OR [HC2 AND ASCUS]													0 (0)	0 (0)	0 (0)	4 (23974)	0 (0)	0 (0)	3 (14917)	0 (0)	3 (14917)	2 (12920)	0 (0)	3 (14917)	2 (12920)
	HPV16/18 OR [PCR AND ≥ASCUS]														1 (5046)	0 (0)	3 (23695)	3 (23695)	0 (0)	0 (0)	2 (12631)	1 (11064)	0 (0)	0 (0)	0 (0)	0 (0)
	mRNA AND ≥ASCUS															0 (0)	1 (5046)	1 (5046)	0 (0)	0 (0)	1 (5046)	0 (0)	0 (0)	0 (0)	0 (0)	0 (0)
	≥HSIL OR [HC2 AND ≥ASCUS]																1 (1755)	0 (0)	0 (0)	1 (1755)	0 (0)	1 (1755)	0 (0)	0 (0)	1 (1755)	1 (1755)
	≥ASCUS																	4 (27646)	1 (7908)	7 (46687)	4 (62941)	7 (29423)	2 (12920)	1 (40901)	6 (18359)	3 (14675)
	PCR AND ≥ASCUS																		0 (0)	0 (0)	2 (12631)	1 (11064)	0 (0)	0 (0)	0 (0)	0 (0)
	≥ASCH																			1 (7908)	0 (0)	0 (0)	0 (0)	0 (0)	0 (0)	0 (0)
	HC2 AND ≥ASCUS																				0 (0)	4 (16672)	2 (12920)	0 (0)	4 (16672)	3 (14675)
	HPV16/18																					0 (0)	0 (0)	1 (40901)	0 (0)	0 (0)

	≥LSIL																						3 (14993)	0 (0)	7 (18840)	3 (14675)
	HC2 AND ≥LSIL																							0 (0)	2 (12920)	2 (12920)
	HPV16/18 AND ≥ASCUS																								0 (0)	0 (0)
	≥HSIL																									3 (14675)
	HC2 AND ≥HSIL																									

*The rows and columns respectively represent the index (the test of interest) and comparator (the test in comparison) tests or combination algorithms. The number of studies followed by the number of participating women in the parentheses who contributed to each comparison are described.

ASCH, atypical squamous cells cannot exclude high-grade lesions; ASCUS, atypical squamous cells of undetermined significance; FN, false negative; FP, false positive; HC2, Hybrid Capture 2; HPV16/18(/45), genotyping for HPV types 16 or 18 (or 45); HSIL, high-grade squamous intraepithelial lesion; LBC, liquid-based cytology; LSIL, low-grade squamous intraepithelial lesion; mRNA, messenger ribonucleic acid; PCR, polymerase chain reaction.

Table S5. Comparative Accuracy of Cervical Cancer Screening Tests or Combination Algorithms.*

Index and comparator tests or combination algorithms		Index (for specificity) and comparator (for sensitivity) tests or combination algorithms																								
		PCR OR ≥ASCUS	HC2 OR ≥ASCUS	mRNA OR ≥ASCUS	PCR	HC2 OR ≥LSIL	HC2 OR ≥HSIL	HC2	mRNA	HPV16/18/45 OR [mRNA AND ≥ASCUS]	≥LSIL OR [PCR AND ASCUS]	HPV16/18 OR ≥ASCUS	≥LSIL OR [HC2 AND ASCUS]	HPV16/18 OR [PCR AND ≥ASCUS]	mRNA AND ≥ASCUS	≥HSIL OR [HC2 AND ≥ASCUS]	≥ASCUS	PCR AND ≥ASCUS	≥ASCH	HC2 AND ≥ASCUS	HPV16/18	≥LSIL	HC2 AND ≥LSIL	HPV16/18 AND ≥ASCUS	≥HSIL	HC2 AND ≥HSIL
Index (for sensitivity) and comparator (for specificity) tests or combination algorithms	PCR OR ≥ASCUS		0.97 (0.94–0.99) [<0.001]	0.96 (0.83–0.99) [0.001]	0.94 (0.87–0.98) [<0.001]	0.93 (0.87–0.96) [<0.001]	0.90 (0.82–0.95) [<0.001]	0.88 (0.82–0.93) [<0.001]	0.87 (0.69–0.96) [<0.001]	0.85 (0.46–0.97) [<0.001]	0.84 (0.67–0.94) [<0.001]	0.75 (0.57–0.88) [<0.001]	0.70 (0.54–0.83) [<0.001]	0.70 (0.52–0.83) [<0.001]	0.66 (0.19–0.92) [<0.001]	0.61 (0.45–0.77) [<0.001]	0.61 (0.50–0.71) [<0.001]	0.58 (0.38–0.75) [<0.001]	0.57 (0.45–0.68) [<0.001]	0.53 (0.38–0.67) [<0.001]	0.52 (0.32–0.72) [<0.001]	0.52 (0.39–0.63) [<0.001]	0.46 (0.30–0.61) [<0.001]	0.37 (0.13–0.63) [<0.001]	0.35 (0.22–0.50) [<0.001]	0.34 (0.18–0.52) [<0.001]
	HC2 OR ≥ASCUS	1.01 (0.93–1.12) [0.453]		0.99 (0.86–1.03) [0.345]	0.97 (0.90–1.02) [0.119]	0.96 (0.91–0.99) [<0.001]	0.94 (0.86–0.98) [<0.001]	0.91 (0.85–0.96) [<0.001]	0.90 (0.72–0.99) [0.010]	0.87 (0.47–1.00) [0.026]	0.87 (0.69–0.98) [0.003]	0.77 (0.59–0.91) [<0.001]	0.72 (0.57–0.85) [<0.001]	0.72 (0.53–0.86) [<0.001]	0.68 (0.20–0.95) [0.002]	0.63 (0.46–0.79) [<0.001]	0.63 (0.52–0.73) [<0.001]	0.60 (0.39–0.77) [<0.001]	0.59 (0.46–0.69) [<0.001]	0.55 (0.40–0.69) [<0.001]	0.54 (0.33–0.74) [<0.001]	0.54 (0.41–0.65) [<0.001]	0.48 (0.32–0.63) [<0.001]	0.38 (0.14–0.65) [<0.001]	0.36 (0.22–0.51) [<0.001]	0.36 (0.19–0.53) [<0.001]
	mRNA OR ≥ASCUS	1.04 (0.89–1.17) [0.286]	1.03 (0.89–1.12) [0.290]		0.99 (0.91–1.13) [0.353]	0.97 (0.91–1.11) [0.257]	0.95 (0.86–1.09) [0.148]	0.93 (0.86–1.06) [0.085]	0.92 (0.73–1.05) [0.090]	0.90 (0.49–1.00) [<0.001]	0.88 (0.70–1.04) [0.061]	0.79 (0.60–0.95) [0.008]	0.74 (0.57–0.89) [0.003]	0.74 (0.55–0.90) [0.003]	0.70 (0.20–0.96) [<0.001]	0.65 (0.47–0.82) [<0.001]	0.64 (0.53–0.77) [<0.001]	0.61 (0.40–0.80) [<0.001]	0.60 (0.47–0.73) [<0.001]	0.56 (0.41–0.72) [<0.001]	0.55 (0.34–0.77) [<0.001]	0.55 (0.42–0.68) [<0.001]	0.49 (0.32–0.65) [<0.001]	0.39 (0.14–0.66) [<0.001]	0.37 (0.23–0.53) [<0.001]	0.36 (0.20–0.55) [<0.001]
	PCR	1.03 (0.96–1.14)	1.03 (0.96–1.10)	1.00 (0.91–1.16)		0.99 (0.92–1.07)	0.96 (0.88–1.05)	0.94 (0.87–1.02)	0.93 (0.73–1.04)	0.90 (0.49–1.05) [0.122]	0.89 (0.72–1.01)	0.80 (0.62–0.93)	0.75 (0.58–0.89)	0.74 (0.56–0.89)	0.70 (0.20–0.99)	0.65 (0.48–0.82)	0.65 (0.54–0.75)	0.62 (0.41–0.79)	0.61 (0.48–0.72)	0.57 (0.41–0.72)	0.56 (0.34–0.76)	0.56 (0.42–0.67)	0.49 (0.33–0.65)	0.40 (0.14–0.66)	0.37 (0.23–0.53)	0.37 (0.20–0.55)

		[0.211]	[0.214]	[0.470]		[0.340]	[0.156]	[0.055]	[0.104]		[0.043]	[0.001]	[<0.001]	[<0.001]	[0.015]	[<0.001]	[<0.001]	[<0.001]	[<0.001]	[<0.001]	[<0.001]	[<0.001]	[<0.001]	[<0.001]	[<0.001]	[<0.001]
	HC2 OR ≥LSIL	1.04 (0.96–1.16) [0.176]	1.03 (1.00–1.09) [<0.001]	1.00 (0.92–1.16) [0.478]	1.01 (0.95–1.08) [0.420]		0.98 (0.91–1.00) [<0.001]	0.95 (0.89–1.01) [0.056]	0.94 (0.75–1.04) [0.152]	0.91 (0.49–1.06) [0.170]	0.91 (0.72–1.03) [0.071]	0.81 (0.62–0.95) [0.002]	0.76 (0.60–0.88) [<0.001]	0.75 (0.56–0.90) [<0.001]	0.71 (0.20–1.00) [0.023]	0.66 (0.49–0.82) [<0.001]	0.66 (0.55–0.76) [<0.001]	0.62 (0.41–0.81) [<0.001]	0.61 (0.49–0.72) [<0.001]	0.58 (0.43–0.71) [<0.001]	0.56 (0.34–0.78) [<0.001]	0.56 (0.43–0.67) [<0.001]	0.50 (0.33–0.65) [<0.001]	0.40 (0.14–0.68) [<0.001]	0.37 (0.24–0.53) [<0.001]	0.37 (0.20–0.55) [<0.001]
	HC2 OR ≥HSIL	1.07 (0.99–1.19) [0.048]	1.06 (1.02–1.12) [<0.001]	1.03 (0.95–1.20) [0.267]	1.03 (0.97–1.11) [0.142]	1.02 (1.00–1.07) [<0.001]		0.98 (0.91–1.06) [0.266]	0.97 (0.76–1.09) [0.303]	0.94 (0.50–1.10) [0.275]	0.93 (0.74–1.07) [0.170]	0.83 (0.64–0.98) [0.013]	0.78 (0.62–0.91) [<0.001]	0.77 (0.57–0.93) [0.002]	0.73 (0.21–1.03) [0.051]	0.68 (0.51–0.84) [<0.001]	0.68 (0.57–0.78) [<0.001]	0.64 (0.42–0.83) [<0.001]	0.63 (0.50–0.74) [<0.001]	0.59 (0.44–0.73) [<0.001]	0.58 (0.35–0.80) [<0.001]	0.58 (0.45–0.69) [<0.001]	0.51 (0.35–0.66) [<0.001]	0.41 (0.15–0.69) [<0.001]	0.38 (0.24–0.55) [<0.001]	0.38 (0.21–0.57) [<0.001]
	HC2	1.07 (1.00–1.19) [0.023]	1.07 (1.03–1.12) [<0.001]	1.03 (0.96–1.20) [0.217]	1.04 (0.99–1.11) [0.076]	1.03 (0.99–1.08) [0.046]	1.00 (0.97–1.04) [0.396]		0.99 (0.79–1.10) [0.423]	0.96 (0.52–1.11) [0.351]	0.95 (0.76–1.08) [0.262]	0.85 (0.65–1.00) [0.021]	0.79 (0.63–0.93) [0.001]	0.79 (0.59–0.95) [0.003]	0.75 (0.22–1.05) [0.076]	0.69 (0.51–0.86) [<0.001]	0.69 (0.58–0.79) [<0.001]	0.66 (0.43–0.84) [<0.001]	0.64 (0.52–0.75) [<0.001]	0.61 (0.45–0.75) [<0.001]	0.59 (0.36–0.81) [<0.001]	0.59 (0.46–0.70) [<0.001]	0.52 (0.35–0.68) [<0.001]	0.42 (0.15–0.71) [<0.001]	0.39 (0.25–0.56) [<0.001]	0.39 (0.21–0.58) [<0.001]
	mRNA	1.09 (1.00–1.21) [0.018]	1.08 (1.02–1.16) [0.008]	1.05 (0.97–1.22) [0.132]	1.05 (0.99–1.13) [0.052]	1.05 (0.98–1.11) [0.065]	1.02 (0.96–1.07) [0.236]	1.02 (0.96–1.06) [0.260]		0.97 (0.53–1.24) [0.405]	0.97 (0.77–1.24) [0.369]	0.86 (0.66–1.11) [0.109]	0.81 (0.62–1.05) [0.049]	0.81 (0.59–1.05) [0.049]	0.76 (0.23–1.12) [0.112]	0.71 (0.52–0.95) [0.012]	0.71 (0.58–0.89) [0.005]	0.67 (0.44–0.91) [0.007]	0.66 (0.51–0.84) [0.002]	0.62 (0.45–0.83) [0.002]	0.60 (0.37–0.86) [0.002]	0.60 (0.45–0.78) [<0.001]	0.54 (0.36–0.74) [<0.001]	0.43 (0.16–0.74) [<0.001]	0.40 (0.25–0.60) [<0.001]	0.40 (0.21–0.62) [<0.001]
	HPV16/18/45 OR [mRNA AND ≥ASCUS]	1.11 (1.02–1.24) [0.008]	1.10 (1.03–1.18) [0.005]	1.07 (1.00–1.24) [<0.001]	1.07 (1.00–1.16) [0.020]	1.07 (1.00–1.13) [0.027]	1.04 (0.97–1.10) [0.099]	1.04 (0.97–1.08) [0.099]	1.02 (0.96–1.08) [0.231]		1.00 (0.77–1.82) [0.493]	0.89 (0.67–1.61) [0.269]	0.84 (0.62–1.54) [0.190]	0.84 (0.60–1.50) [0.189]	0.83 (0.28–0.99) [<0.001]	0.74 (0.52–1.36) [0.103]	0.73 (0.58–1.33) [0.089]	0.70 (0.45–1.25) [0.077]	0.68 (0.52–1.24) [0.065]	0.65 (0.45–1.18) [0.055]	0.64 (0.38–1.15) [0.051]	0.63 (0.46–1.14) [0.044]	0.56 (0.36–1.04) [0.029]	0.46 (0.16–0.94) [0.018]	0.42 (0.26–0.80) [0.008]	0.42 (0.22–0.80) [0.008]

	≥LSIL OR [PCR AND ASCUS]	1.08 (1.01–1.19) [<0.001]	1.07 (1.00–1.15) [0.026]	1.04 (0.95–1.21) [0.206]	1.04 (0.97–1.12) [0.098]	1.04 (0.97–1.10) [0.132]	1.01 (0.94–1.07) [0.347]	1.01 (0.94–1.05) [0.390]	0.99 (0.92–1.06) [0.401]	0.97 (0.91–1.04) [0.177]		0.90 (0.73–0.99) [<0.001]	0.84 (0.64–1.08) [0.077]	0.84 (0.65–0.97) [<0.001]	0.79 (0.23–1.17) [0.164]	0.74 (0.53–0.98) [0.018]	0.73 (0.59–0.92) [0.004]	0.70 (0.47–0.88) [<0.001]	0.68 (0.53–0.86) [0.002]	0.64 (0.46–0.85) [0.002]	0.63 (0.38–0.88) [0.002]	0.62 (0.47–0.80) [<0.001]	0.56 (0.36–0.77) [<0.001]	0.45 (0.16–0.74) [<0.001]	0.42 (0.26–0.61) [<0.001]	0.42 (0.22–0.64) [<0.001]
	HPV16/18 OR ≥ASCUS	1.10 (1.03–1.22) [<0.001]	1.09 (1.02–1.16) [0.004]	1.06 (0.97–1.23) [0.102]	1.06 (1.00–1.14) [0.027]	1.06 (0.99–1.12) [0.039]	1.03 (0.97–1.08) [0.156]	1.02 (0.97–1.07) [0.171]	1.01 (0.95–1.07) [0.382]	0.99 (0.93–1.05) [0.315]	1.01 (1.00–1.06) [<0.001]		0.94 (0.71–1.26) [0.311]	0.95 (0.78–1.00) [<0.001]	0.88 (0.26–1.34) [0.331]	0.82 (0.59–1.13) [0.106]	0.82 (0.67–1.06) [0.054]	0.78 (0.55–0.95) [<0.001]	0.76 (0.59–1.00) [0.024]	0.72 (0.51–0.98) [0.018]	0.70 (0.44–0.99) [0.022]	0.70 (0.53–0.92) [0.007]	0.62 (0.41–0.88) [0.003]	0.50 (0.19–0.81) [<0.001]	0.47 (0.29–0.70) [<0.001]	0.47 (0.25–0.73) [<0.001]
	≥LSIL OR [HC2 AND ASCUS]	1.13 (1.06–1.27) [<0.001]	1.13 (1.08–1.20) [<0.001]	1.09 (1.02–1.28) [0.002]	1.10 (1.05–1.18) [<0.001]	1.09 (1.05–1.15) [<0.001]	1.06 (1.03–1.11) [<0.001]	1.06 (1.03–1.09) [<0.001]	1.04 (1.00–1.11) [0.015]	1.02 (0.98–1.09) [0.159]	1.05 (1.01–1.13) [0.007]	1.03 (1.00–1.09) [0.031]		1.00 (0.72–1.33) [0.490]	0.94 (0.27–1.44) [0.423]	0.89 (0.68–0.99) [<0.001]	0.88 (0.71–1.10) [0.113]	0.83 (0.54–1.16) [0.133]	0.81 (0.64–1.03) [0.044]	0.77 (0.59–0.93) [<0.001]	0.75 (0.45–1.09) [0.071]	0.75 (0.57–0.95) [0.009]	0.67 (0.46–0.85) [<0.001]	0.53 (0.19–0.93) [0.011]	0.50 (0.32–0.72) [<0.001]	0.50 (0.27–0.72) [<0.001]
	HPV16/18 OR [PCR AND ≥ASCUS]	1.12 (1.06–1.25) [<0.001]	1.12 (1.07–1.19) [<0.001]	1.08 (1.01–1.27) [0.009]	1.09 (1.04–1.16) [<0.001]	1.08 (1.04–1.14) [<0.001]	1.05 (1.01–1.11) [0.005]	1.05 (1.02–1.09) [0.004]	1.03 (0.99–1.09) [0.055]	1.01 (0.97–1.08) [0.290]	1.04 (1.01–1.11) [<0.001]	1.02 (1.00–1.08) [<0.001]	0.99 (0.96–1.02) [0.252]		0.94 (0.28–1.46) [0.431]	0.88 (0.63–1.24) [0.224]	0.88 (0.71–1.16) [0.157]	0.84 (0.60–0.99) [<0.001]	0.82 (0.63–1.09) [0.078]	0.77 (0.55–1.07) [0.060]	0.75 (0.47–1.07) [0.063]	0.75 (0.56–1.01) [0.028]	0.67 (0.43–0.96) [0.015]	0.54 (0.21–0.86) [<0.001]	0.50 (0.31–0.76) [0.001]	0.50 (0.27–0.80) [0.001]
	mRNA AND ≥ASCUS	1.14 (1.06–1.27) [<0.001]	1.13 (1.07–1.21) [<0.001]	1.10 (1.02–1.28) [<0.001]	1.10 (1.04–1.18) [<0.001]	1.09 (1.04–1.16) [0.001]	1.07 (1.02–1.12) [0.005]	1.06 (1.02–1.11) [0.005]	1.05 (1.00–1.11) [0.029]	1.02 (1.00–1.08) [<0.001]	1.05 (1.00–1.13) [0.020]	1.04 (0.99–1.10) [0.054]	1.00 (0.96–1.03) [0.425]	1.01 (0.97–1.05) [0.243]		0.94 (0.58–3.21) [0.431]	0.93 (0.63–3.18) [0.419]	0.88 (0.52–3.00) [0.376]	0.86 (0.57–2.96) [0.356]	0.82 (0.51–2.77) [0.309]	0.80 (0.45–2.68) [0.297]	0.79 (0.51–2.69) [0.290]	0.71 (0.41–2.42) [0.225]	0.59 (0.20–2.02) [0.152]	0.54 (0.30–1.83) [0.115]	0.54 (0.26–1.83) [0.118]
	≥HSIL OR	1.15	1.15	1.11	1.11	1.11	1.08	1.07	1.06	1.04 (1.00–	1.07	1.05	1.01	1.02	1.01		1.00	0.94	0.93	0.89	0.85	0.85	0.76	0.61	0.57	0.57

	[HC2 AND ≥ASCUS]	(1.07–1.29) [<0.001]	(1.10–1.22) [<0.001]	(1.04–1.30) [<0.001]	(1.06–1.20) [<0.001]	(1.07–1.17) [<0.001]	(1.04–1.13) [<0.001]	(1.05–1.11) [<0.001]	(1.02–1.12) [<0.001]	1.11) [0.025]	(1.02–1.14) [<0.001]	(1.02–1.11) [0.001]	(1.00–1.03) [<0.001]	(1.00–1.06) [0.023]	(0.99–1.05) [0.224]		(0.79–1.32) [0.491]	(0.60–1.38) [0.376]	(0.71–1.24) [0.297]	(0.68–0.99) [<0.001]	(0.50–1.29) [0.237]	(0.64–1.13) [0.129]	(0.53–0.95) [<0.001]	(0.22–1.09) [0.052]	(0.36–0.84) [0.001]	(0.32–0.83) [<0.001]
	≥ASCUS	1.11 (1.04–1.24) [<0.001]	1.11 (1.07–1.17) [<0.001]	1.07 (1.01–1.25) [0.014]	1.08 (1.03–1.15) [<0.001]	1.07 (1.04–1.12) [<0.001]	1.04 (1.01–1.09) [0.001]	1.04 (1.02–1.07) [<0.001]	1.02 (0.99–1.08) [0.096]	1.00 (0.97–1.07) [0.446]	1.03 (0.99–1.10) [0.056]	1.02 (0.99–1.07) [0.185]	0.98 (0.96–1.00) [0.036]	0.99 (0.97–1.02) [0.231]	0.98 (0.95–1.02) [0.123]	0.97 (0.95–0.98) [<0.001]		0.95 (0.64–1.24) [0.361]	0.94 (0.79–1.00) [<0.001]	0.88 (0.66–1.09) [0.126]	0.85 (0.53–1.18) [0.192]	0.86 (0.69–0.97) [<0.001]	0.76 (0.52–0.99) [0.021]	0.61 (0.22–1.02) [0.032]	0.57 (0.37–0.79) [<0.001]	0.57 (0.32–0.83) [0.001]
	PCR AND ≥ASCUS	1.14 (1.07–1.27) [<0.001]	1.14 (1.08–1.21) [<0.001]	1.10 (1.03–1.29) [<0.001]	1.11 (1.06–1.18) [<0.001]	1.10 (1.06–1.16) [<0.001]	1.07 (1.03–1.12) [<0.001]	1.07 (1.04–1.10) [<0.001]	1.05 (1.01–1.11) [0.003]	1.03 (0.99–1.09) [0.066]	1.06 (1.02–1.13) [<0.001]	1.04 (1.01–1.10) [<0.001]	1.01 (0.98–1.03) [0.285]	1.01 (1.00–1.04) [<0.001]	1.00 (0.98–1.04) [0.424]	0.99 (0.97–1.01) [0.193]	1.02 (1.01–1.04) [0.008]		0.98 (0.73–1.47) [0.459]	0.93 (0.63–1.43) [0.347]	0.90 (0.56–1.40) [0.317]	0.90 (0.65–1.35) [0.278]	0.80 (0.51–1.27) [0.160]	0.66 (0.25–0.98) [<0.001]	0.60 (0.36–1.00) [0.024]	0.60 (0.32–1.03) [0.032]
	≥ASCH	1.15 (1.07–1.28) [<0.001]	1.14 (1.09–1.22) [<0.001]	1.11 (1.03–1.29) [<0.001]	1.11 (1.06–1.19) [<0.001]	1.10 (1.06–1.16) [<0.001]	1.08 (1.04–1.13) [<0.001]	1.07 (1.05–1.11) [<0.001]	1.05 (1.02–1.12) [0.001]	1.03 (1.00–1.10) [0.040]	1.06 (1.02–1.14) [<0.001]	1.05 (1.01–1.11) [0.002]	1.01 (0.99–1.03) [0.103]	1.02 (1.00–1.05) [0.042]	1.01 (0.98–1.05) [0.300]	1.00 (0.98–1.01) [0.335]	1.03 (1.01–1.05) [<0.001]	1.00 (0.99–1.03) [0.293]		0.94 (0.70–1.21) [0.315]	0.92 (0.57–1.31) [0.329]	0.93 (0.75–1.00) [<0.001]	0.82 (0.56–1.08) [0.085]	0.66 (0.24–1.12) [0.073]	0.61 (0.40–0.86) [<0.001]	0.61 (0.34–0.91) [0.006]
	HC2 AND ≥ASCUS	1.16 (1.08–1.30) [<0.001]	1.15 (1.10–1.23) [<0.001]	1.12 (1.04–1.30) [<0.001]	1.12 (1.07–1.20) [<0.001]	1.11 (1.07–1.17) [<0.001]	1.08 (1.05–1.13) [<0.001]	1.08 (1.05–1.12) [<0.001]	1.06 (1.03–1.13) [<0.001]	1.04 (1.01–1.11) [0.006]	1.07 (1.03–1.15) [<0.001]	1.05 (1.02–1.12) [<0.001]	1.02 (1.01–1.04) [<0.001]	1.03 (1.01–1.06) [0.002]	1.02 (0.99–1.06) [0.111]	1.00 (1.00–1.02) [<0.001]	1.04 (1.02–1.06) [<0.001]	1.01 (1.00–1.03) [0.048]	1.01 (1.00–1.02) [0.081]		0.97 (0.58–1.48) [0.458]	0.97 (0.74–1.30) [0.418]	0.89 (0.64–0.99) [<0.001]	0.70 (0.25–1.25) [0.132]	0.65 (0.42–0.96) [0.015]	0.65 (0.37–0.91) [<0.001]
	HPV16/18	1.14 (1.07–	1.14 (1.08–	1.10 (1.03–	1.11 (1.06–	1.10 (1.05–	1.07 (1.03–	1.06 (1.04–	1.05 (1.01–	1.03 (0.99–1.09) [0.077]	1.06 (1.02–	1.04 (1.01–	1.01 (0.98–	1.01 (0.99–	1.00 (0.98–	0.99 (0.97–	1.02 (1.00–	1.00 (0.98–	1.00 (0.97–	0.99 (0.97–		1.00 (0.68–	0.89 (0.54–	0.72 (0.27–	0.67 (0.39–	0.66 (0.34–

		1.27) [<0.001]	1.21) [<0.001]	1.29) [<0.001]	1.18) [<0.001]	1.16) [<0.001]	1.12) [<0.001]	1.10) [<0.001]	1.11) [0.004]		1.13) [0.001]	1.10) [0.005]	1.03) [0.299]	1.04) [0.101]	1.04) [0.431]	1.01) [0.194]	1.04) [0.015]	1.02) [0.489]	1.01) [0.297]	1.00) [0.054]		1.62) [0.495]	1.53) [0.317]	1.34) [0.153]	1.18) [0.076]	1.23) [0.093]
	≥LSIL	1.15 (1.08–1.29) [<0.001]	1.15 (1.10–1.22) [<0.001]	1.11 (1.04–1.30) [<0.001]	1.12 (1.07–1.20) [<0.001]	1.11 (1.07–1.17) [<0.001]	1.08 (1.05–1.13) [<0.001]	1.08 (1.05–1.11) [<0.001]	1.06 (1.02–1.13) [<0.001]	1.04 (1.00–1.11) [0.010]	1.07 (1.03–1.15) [<0.001]	1.05 (1.02–1.11) [<0.001]	1.02 (1.00–1.04) [0.006]	1.03 (1.01–1.06) [0.003]	1.01 (0.99–1.06) [0.157]	1.00 (0.99–1.02) [0.361]	1.03 (1.02–1.05) [<0.001]	1.01 (1.00–1.03) [0.081]	1.00 (1.00–1.02) [<0.001]	1.00 (0.99–1.01) [0.276]	1.01 (1.00–1.03) [0.095]		0.89 (0.61–1.21) [0.234]	0.72 (0.26–1.24) [0.144]	0.67 (0.45–0.94) [<0.001]	0.67 (0.37–1.01) [0.026]
	HC2 AND ≥LSIL	1.16 (1.09–1.30) [<0.001]	1.16 (1.11–1.24) [<0.001]	1.12 (1.05–1.31) [<0.001]	1.13 (1.07–1.21) [<0.001]	1.12 (1.08–1.18) [<0.001]	1.09 (1.06–1.14) [<0.001]	1.09 (1.06–1.12) [<0.001]	1.07 (1.03–1.14) [<0.001]	1.05 (1.01–1.12) [<0.001]	1.08 (1.04–1.16) [<0.001]	1.06 (1.03–1.12) [<0.001]	1.03 (1.01–1.05) [<0.001]	1.03 (1.01–1.07) [<0.001]	1.02 (1.00–1.07) [0.029]	1.01 (1.00–1.03) [<0.001]	1.04 (1.03–1.07) [<0.001]	1.02 (1.00–1.04) [0.004]	1.01 (1.00–1.03) [0.003]	1.01 (1.00–1.01) [<0.001]	1.02 (1.00–1.04) [0.005]	1.01 (1.00–1.02) [0.014]		0.81 (0.29–1.51) [0.276]	0.75 (0.48–1.18) [0.106]	0.77 (0.44–0.98) [<0.001]
	HPV16/18 AND ≥ASCUS	1.17 (1.09–1.31) [<0.001]	1.16 (1.11–1.24) [<0.001]	1.13 (1.05–1.32) [<0.001]	1.13 (1.08–1.22) [<0.001]	1.12 (1.08–1.19) [<0.001]	1.09 (1.06–1.15) [<0.001]	1.09 (1.06–1.13) [<0.001]	1.07 (1.03–1.14) [<0.001]	1.05 (1.02–1.12) [0.001]	1.08 (1.04–1.16) [<0.001]	1.06 (1.03–1.13) [<0.001]	1.03 (1.01–1.05) [0.001]	1.04 (1.02–1.07) [<0.001]	1.03 (1.00–1.07) [0.018]	1.01 (1.00–1.03) [0.027]	1.05 (1.03–1.07) [<0.001]	1.02 (1.01–1.04) [<0.001]	1.02 (1.00–1.04) [0.013]	1.01 (1.00–1.02) [0.062]	1.02 (1.01–1.05) [0.004]	1.01 (1.00–1.02) [0.033]	1.00 (0.99–1.01) [0.243]		0.94 (0.48–2.70) [0.441]	0.93 (0.42–2.69) [0.437]
	≥HSIL	1.17 (1.10–1.32) [<0.001]	1.17 (1.11–1.25) [<0.001]	1.13 (1.06–1.32) [<0.001]	1.14 (1.08–1.23) [<0.001]	1.13 (1.08–1.20) [<0.001]	1.10 (1.06–1.16) [<0.001]	1.10 (1.07–1.14) [<0.001]	1.08 (1.04–1.15) [<0.001]	1.06 (1.02–1.13) [<0.001]	1.09 (1.05–1.17) [<0.001]	1.07 (1.04–1.13) [<0.001]	1.03 (1.02–1.06) [<0.001]	1.04 (1.02–1.08) [<0.001]	1.03 (1.01–1.08) [<0.001]	1.02 (1.01–1.04) [<0.001]	1.05 (1.04–1.08) [<0.001]	1.03 (1.01–1.05) [<0.001]	1.02 (1.01–1.04) [<0.001]	1.02 (1.01–1.02) [<0.001]	1.03 (1.01–1.05) [<0.001]	1.02 (1.01–1.03) [<0.001]	1.01 (1.00–1.02) [0.002]	1.00 (1.00–1.02) [0.119]		0.99 (0.54–1.68) [0.487]
	HC2 AND ≥HSIL	1.17 (1.10–1.32)	1.17 (1.11–1.25)	1.13 (1.06–1.32)	1.14 (1.08–1.23)	1.13 (1.08–1.20)	1.10 (1.06–1.16)	1.10 (1.07–1.14)	1.08 (1.04–1.15)	1.06 (1.02–1.13) [<0.001]	1.09 (1.05–1.17)	1.07 (1.04–1.13)	1.03 (1.02–1.06)	1.04 (1.02–1.08)	1.03 (1.01–1.08)	1.02 (1.01–1.04)	1.05 (1.04–1.08)	1.03 (1.01–1.05)	1.02 (1.01–1.04)	1.02 (1.01–1.03)	1.03 (1.01–1.05)	1.02 (1.01–1.03)	1.01 (1.00–1.02)	1.00 (1.00–1.02)	1.00 (1.00–1.00)	

		[<0.001]	[<0.001]	[<0.001]	[<0.001]	[<0.001]	[<0.001]	[<0.001]	[<0.001]		[<0.001]	[<0.001]	[<0.001]	[<0.001]	[<0.001]	[<0.001]	[<0.001]	[<0.001]	[<0.001]	[<0.001]	[<0.001]	[<0.001]	[<0.001]	[0.121]	[0.457]	

*Above the diagonal line (formed by gray cells) represents relative sensitivity (95% CrI) [probability that relative sensitivity is ≥1] and below the diagonal line represents relative specificity (95% CrI) [probability that relative specificity is ≤1]. For relative sensitivity, the rows and columns, respectively, represent the index (the test of interest) and comparator (the test in comparison) tests or combination algorithms. For relative sensitivity, the columns and rows, respectively, represent the index and comparator tests or combination algorithms.

ASCH, atypical squamous cells cannot exclude high-grade lesion; ASCUS, atypical squamous cells of undetermined significance; CrI, credible interval; HC2, Hybrid Capture 2; HPV16/18(/45), genotyping for HPV types 16 or 18 (or 45); HSIL, high-grade squamous intraepithelial lesion; LBC, liquid-based cytology; LSIL, low-grade squamous intraepithelial lesion; mRNA, messenger ribonucleic acid; PCR, polymerase chain reaction.

Table S6. Difference in False Negatives or Positives between Alternative Cervical Cancer Screening Tests or Combination Algorithms.*

Index and comparator tests or combination algorithms		Index (for FN) and comparator (for FP) tests or combination algorithms																								
		PCR OR ≥ASCUS	HC2 OR ≥ASCUS	mRNA OR ≥ASCUS	PCR	HC2 OR ≥LSIL	HC2 OR ≥HSIL	HC2	mRNA	HPV16/18/45 OR [mRNA AND ≥ASCUS]	≥LSIL OR [PCR AND ASCUS]	HPV16/18 OR ≥ASCUS	≥LSIL OR [HC2 AND ASCUS]	HPV16/18 OR [PCR AND ≥ASCUS]	mRNA AND ≥ASCUS	≥HSIL OR [HC2 AND ≥ASCUS]	≥ASCUS	PCR AND ≥ASCUS	≥ASCH	HC2 AND ≥ASCUS	HPV16/18	≥LSIL	HC2 AND ≥LSIL	HPV16/18 AND ≥ASCUS	≥HSIL	HC2 AND ≥HSIL
Index (for FP) and comparator (for FN) tests or combination algorithms	PCR OR ≥ASCUS		+1 (+0 to +1)	+1 (+0 to +3)	+1 (+0 to +3)	+1 (+1 to +3)	+2 (+1 to +4)	+2 (+1 to +4)	+3 (+1 to +6)	+3 (+1 to +11)	+3 (+1 to +7)	+5 (+2 to +9)	+6 (+3 to +9)	+6 (+3 to +10)	+7 (+2 to +16)	+8 (+5 to +11)	+8 (+6 to +10)	+8 (+5 to +12)	+9 (+6 to +11)	+9 (+7 to +12)	+10 (+6 to +14)	+10 (+7 to +12)	+11 (+8 to +14)	+13 (+7 to +17)	+13 (+10 to +16)	+13 (+10 to +16)
	HC2 OR ≥ASCUS	-4 (-88 to +64)		+0 (-1 to +3)	+1 (-0 to +2)	+1 (+0 to +2)	+1 (+0 to +3)	+2 (+1 to +3)	+2 (+0 to +6)	+2 (-0 to +10)	+3 (+0 to +6)	+4 (+2 to +8)	+5 (+3 to +8)	+5 (+3 to +9)	+6 (+1 to +16)	+7 (+4 to +10)	+7 (+5 to +9)	+8 (+4 to +12)	+8 (+6 to +10)	+9 (+6 to +11)	+9 (+5 to +13)	+9 (+7 to +11)	+10 (+7 to +13)	+12 (+7 to +17)	+12 (+9 to +15)	+12 (+9 to +16)
	mRNA OR ≥ASCUS	-29 (-130 to +96)	-26 (-98 to +92)		+0 (-2 to +2)	+1 (-2 to +2)	+1 (-1 to +3)	+1 (-1 to +3)	+2 (-1 to +5)	+2 (+0 to +10)	+2 (-1 to +6)	+4 (+1 to +8)	+5 (+2 to +8)	+5 (+2 to +9)	+6 (+1 to +15)	+7 (+3 to +10)	+7 (+4 to +9)	+7 (+4 to +11)	+8 (+5 to +10)	+8 (+5 to +11)	+9 (+4 to +13)	+9 (+6 to +11)	+10 (+6 to +13)	+11 (+6 to +16)	+12 (+9 to +15)	+12 (+8 to +15)
	PCR	-27 (-108 to +37)	-23 (-80 to +37)	+3 (-119 to +81)		+0 (-1 to +2)	+1 (-1 to +2)	+1 (-0 to +2)	+1 (-1 to +5)	+2 (-1 to +10)	+2 (-0 to +5)	+4 (+1 to +7)	+5 (+2 to +8)	+5 (+2 to +8)	+6 (+0 to +15)	+6 (+3 to +10)	+7 (+5 to +9)	+7 (+4 to +11)	+7 (+5 to +10)	+8 (+5 to +11)	+8 (+4 to +12)	+8 (+6 to +11)	+10 (+6 to +13)	+11 (+6 to +16)	+12 (+9 to +14)	+12 (+8 to +15)
	HC2 OR ≥LSIL	-32 (-119 to +33)	-26 (-70 to -2)	-2 (-121 to +68)	-5 (-65 to +48)		+0 (+0 to +2)	+1 (-0 to +2)	+1 (-1 to +5)	+2 (-1 to +9)	+2 (-0 to +5)	+4 (+1 to +7)	+5 (+2 to +7)	+5 (+2 to +8)	+5 (+0 to +15)	+6 (+3 to +9)	+6 (+4 to +8)	+7 (+4 to +11)	+7 (+5 to +9)	+8 (+5 to +10)	+8 (+4 to +12)	+8 (+6 to +10)	+9 (+7 to +12)	+11 (+6 to +16)	+12 (+9 to +14)	+12 (+8 to +15)
	HC2 OR ≥HSIL	-55 (-140 to +9)	-50 (-96 to -16)	-25 (-145 to +43)	-28 (-88 to	-21 (-58 to -		+0 (-1 to +2)	+1 (-2 to +4)	+1 (-2 to +9)	+1 (-1 to +5)	+3 (+0 to +7)	+4 (+2 to +7)	+4 (+1 to +8)	+5 (-1 to +14)	+6 (+3 to +9)	+6 (+4 to +8)	+6 (+3 to +10)	+7 (+5 to +9)	+7 (+5 to +10)	+8 (+4 to +12)	+8 (+5 to	+9 (+6 to	+11 (+5 to +15)	+11 (+8 to	+11 (+8 to

					+22)	1)																+10)	+12)		+14)	+14)
	HC2	-59 (-144 to -1)	-55 (-96 to -23)	-28 (-148 to +34)	-32 (-88 to +11)	-26 (-64 to +5)	-4 (-37 to +26)		+0 (-2 to +4)	+1 (-2 to +8)	+1 (-1 to +4)	+3 (+0 to +6)	+4 (+1 to +7)	+4 (+1 to +7)	+4 (-1 to +14)	+5 (+2 to +8)	+5 (+4 to +7)	+6 (+3 to +10)	+6 (+4 to +8)	+7 (+4 to +10)	+7 (+3 to +11)	+7 (+5 to +9)	+8 (+6 to +11)	+10 (+5 to +15)	+11 (+8 to +13)	+11 (+7 to +14)
	mRNA	-72 (-158 to -4)	-68 (-122 to -15)	-42 (-161 to +28)	-45 (-105 to +11)	-40 (-90 to +14)	-18 (-64 to +36)	-14 (-51 to +35)		+1 (-4 to +8)	+1 (-3 to +4)	+2 (-2 to +6)	+3 (-1 to +7)	+3 (-1 to +7)	+4 (-2 to +13)	+5 (+1 to +9)	+5 (+2 to +8)	+6 (+1 to +10)	+6 (+2 to +9)	+7 (+3 to +10)	+7 (+2 to +11)	+7 (+3 to +10)	+8 (+4 to +12)	+10 (+4 to +15)	+10 (+6 to +14)	+10 (+6 to +14)
	HPV16/18/45 OR [mRNA AND ≥ASCUS]	-91 (-178 to -20)	-87 (-145 to -27)	-58 (-182 to -4)	-64 (-126 to -3)	-59 (-111 to +1)	-37 (-85 to +23)	-33 (-73 to +23)	-19 (-73 to +39)		+0 (-8 to +4)	+2 (-6 to +6)	+3 (-5 to +7)	+3 (-5 to +7)	+3 (+0 to +12)	+4 (-3 to +9)	+5 (-3 to +8)	+5 (-2 to +10)	+5 (-2 to +9)	+6 (-2 to +10)	+6 (-1 to +11)	+6 (-1 to +10)	+7 (-0 to +12)	+9 (+1 to +15)	+10 (+2 to +14)	+10 (+2 to +14)
	≥LSIL OR [PCR AND ASCUS]	-64 (-145 to -8)	-61 (-118 to +1)	-35 (-158 to +45)	-38 (-98 to +22)	-33 (-85 to +29)	-11 (-59 to +52)	-7 (-48 to +51)	+7 (-49 to +69)	+26 (-35 to +88)		+2 (+0 to +5)	+3 (-1 to +6)	+3 (+1 to +6)	+3 (-2 to +13)	+4 (+0 to +8)	+4 (+1 to +7)	+5 (+2 to +9)	+5 (+2 to +8)	+6 (+2 to +10)	+6 (+2 to +11)	+6 (+3 to +9)	+7 (+3 to +11)	+9 (+4 to +14)	+10 (+6 to +13)	+10 (+5 to +14)
	HPV16/18 OR ≥ASCUS	-79 (-160 to -22)	-75 (-129 to -20)	-49 (-172 to +24)	-52 (-110 to +1)	-48 (-96 to +6)	-25 (-70 to +28)	-21 (-57 to +28)	-7 (-61 to +47)	+12 (-47 to +65)	-11 (-52 to -0)		+1 (-3 to +5)	+1 (+0 to +3)	+2 (-4 to +11)	+3 (-2 to +7)	+3 (-1 to +6)	+3 (+1 to +7)	+4 (+0 to +7)	+4 (+0 to +8)	+4 (+0 to +9)	+5 (+1 to +8)	+6 (+2 to +10)	+7 (+3 to +12)	+8 (+4 to +11)	+8 (+3 to +12)
	≥LSIL OR [HC2 AND ASCUS]	-112 (-199 to -52)	-107 (-155 to -71)	-81 (-203 to -19)	-84 (-144 to -41)	-79 (-120 to -47)	-56 (-93 to -28)	-52 (-80 to -30)	-38 (-91 to -4)	-19 (-78 to +16)	-46 (-105 to -7)	-31 (-81 to +1)		+0 (-4 to +4)	+1 (-5 to +10)	+1 (+0 to +5)	+2 (-1 to +5)	+2 (-2 to +7)	+3 (-0 to +6)	+3 (+1 to +6)	+4 (-1 to +8)	+4 (+1 to +7)	+5 (+2 to +8)	+6 (+1 to +12)	+7 (+4 to +10)	+7 (+4 to +11)
	HPV16/18 OR [PCR AND ≥ASCUS]	-103 (-184 to -49)	-99 (-150 to -57)	-72 (-195 to -10)	-76 (-131 to -35)	-71 (-116 to -32)	-48 (-89 to -12)	-44 (-75 to -14)	-30 (-81 to +6)	-11 (-67 to +25)	-37 (-92 to -6)	-22 (-66 to -1)	+8 (-16 to +38)		+1 (-5 to +10)	+2 (-3 to +6)	+2 (-2 to +5)	+2 (+0 to +6)	+3 (-1 to +6)	+3 (-1 to +7)	+3 (-1 to +8)	+4 (-0 to +7)	+5 (+0 to +9)	+6 (+2 to +11)	+7 (+3 to +11)	+7 (+2 to +11)

	mRNA AND ≥ASCUS	-113 (-202 to -51)	-110 (-166 to -60)	-83 (-207 to -22)	-86 (-147 to -39)	-82 (-131 to -35)	-59 (-104 to -14)	-55 (-90 to -16)	-41 (-93 to +2)	-19 (-72 to -1)	-48 (-109 to -2)	-34 (-85 to +9)	-3 (-32 to +36)	-11 (-43 to +26)		+1 (-9 to +7)	+1 (-8 to +7)	+2 (-8 to +8)	+2 (-8 to +8)	+2 (-7 to +9)	+3 (-7 to +9)	+3 (-7 to +9)	+4 (-6 to +10)	+5 (-5 to +13)	+6 (-3 to +12)	+6 (-4 to +13)
	≥HSIL OR [HC2 AND ≥ASCUS]	-125 (-214 to -66)	-121 (-171 to -84)	-95 (-217 to -33)	-98 (-158 to -55)	-93 (-136 to -59)	-70 (-108 to -41)	-66 (-94 to -44)	-52 (-104 to -18)	-33 (-91 to +0)	-59 (-119 to -22)	-44 (-95 to -14)	-13 (-31 to -1)	-21 (-50 to -0)	-10 (-49 to +14)		+0 (-3 to +3)	+1 (-4 to +5)	+1 (-2 to +4)	+1 (+0 to +4)	+2 (-3 to +7)	+2 (-1 to +5)	+3 (+1 to +6)	+5 (-1 to +10)	+5 (+2 to +9)	+5 (+2 to +9)
	≥ASCUS	-95 (-179 to -39)	-91 (-136 to -57)	-64 (-184 to -5)	-68 (-123 to -30)	-63 (-102 to -32)	-40 (-75 to -13)	-36 (-59 to -18)	-22 (-71 to +9)	-3 (-59 to +29)	-29 (-86 to +6)	-14 (-62 to +14)	+17 (-2 to +34)	+8 (-17 to +28)	+20 (-17 to +45)	+30 (+15 to +47)		+1 (-3 to +4)	+1 (+0 to +3)	+2 (-1 to +4)	+2 (-2 to +6)	+2 (+0 to +4)	+3 (+0 to +6)	+5 (-0 to +10)	+5 (+2 to +8)	+5 (+2 to +9)
	PCR AND ≥ASCUS	-117 (-201 to -63)	-113 (-164 to -74)	-87 (-211 to -26)	-90 (-146 to -52)	-85 (-131 to -49)	-62 (-103 to -30)	-58 (-89 to -34)	-44 (-95 to -11)	-25 (-81 to +7)	-52 (-108 to -18)	-37 (-83 to -10)	-6 (-28 to +15)	-13 (-37 to -1)	-3 (-39 to +22)	+7 (-10 to +27)	-22 (-41 to -5)		+0 (-4 to +4)	+1 (-4 to +5)	+1 (-4 to +6)	+1 (-3 to +5)	+2 (-2 to +7)	+4 (+0 to +9)	+5 (+0 to +9)	+5 (-0 to +9)
	≥ASCH	-122 (-210 to -64)	-118 (-168 to -80)	-92 (-214 to -31)	-95 (-154 to -53)	-90 (-134 to -56)	-67 (-106 to -37)	-63 (-90 to -41)	-49 (-101 to -16)	-29 (-89 to +3)	-56 (-116 to -20)	-42 (-92 to -11)	-10 (-30 to +7)	-18 (-47 to +3)	-7 (-46 to +17)	+3 (-12 to +19)	-27 (-44 to -11)	-5 (-24 to +14)		+1 (-2 to +4)	+1 (-3 to +5)	+1 (+0 to +3)	+2 (-1 to +5)	+4 (-1 to +9)	+4 (+2 to +7)	+4 (+1 to +8)
	HC2 AND ≥ASCUS	-130 (-219 to -71)	-126 (-176 to -88)	-99 (-222 to -39)	-103 (-163 to -61)	-98 (-141 to -64)	-74 (-113 to -46)	-70 (-99 to -50)	-56 (-109 to -24)	-37 (-97 to -6)	-64 (-125 to -28)	-49 (-100 to -20)	-18 (-36 to -6)	-26 (-55 to -8)	-15 (-54 to +7)	-4 (-16 to -0)	-34 (-51 to -22)	-12 (-31 to +2)	-7 (-23 to +3)		+0 (-4 to +5)	+0 (-3 to +3)	+1 (+0 to +4)	+3 (-2 to +9)	+4 (+0 to +7)	+4 (+1 to +7)
	HPV16/18	-117 (-203 to -	-113 (-164 to -	-86 (-210 to -25)	-90 (-147	-84 (-130 to	-62 (-102 to	-58 (-89 to	-44 (-94 to -	-24 (-81 to +8)	-51 (-109 to -	-36 (-84 to -7)	-5 (-28 to +17)	-14 (-40 to +8)	-2 (-39 to +24)	+8 (-10 to +30)	-22 (-41 to -2)	+0 (-19 to +20)	+5 (-14 to +26)	+12 (-2 to +33)		+0 (-4 to +4)	+1 (-4 to +6)	+3 (-3 to +9)	+3 (-1 to +8)	+3 (-2 to +8)

		60)	73)		to -50)	-48)	-29)	-32)	11)		15)															
	≥LSIL	-127 (-216 to -70)	-123 (-174 to -85)	-97 (-220 to -37)	-100 (-160 to -59)	-95 (-139 to -62)	-72 (-110 to -44)	-68 (-96 to -47)	-54 (-107 to -22)	-35 (-94 to -4)	-61 (-122 to -26)	-47 (-97 to -18)	-15 (-34 to -3)	-24 (-52 to -6)	-12 (-51 to +9)	-2 (-16 to +8)	-32 (-48 to -21)	-9 (-28 to +4)	-4 (-19 to -0)	+2 (-6 to +11)	-10 (-30 to +4)		+1 (-2 to +4)	+3 (-2 to +8)	+3 (+1 to +6)	+3 (-0 to +7)
	HC2 AND ≥LSIL	-136 (-225 to -77)	-132 (-184 to -93)	-105 (-229 to -44)	-109 (-170 to -67)	-104 (-149 to -69)	-81 (-120 to -51)	-77 (-107 to -55)	-63 (-116 to -30)	-43 (-103 to -12)	-70 (-131 to -34)	-55 (-106 to -26)	-24 (-43 to -11)	-32 (-61 to -14)	-21 (-60 to +0)	-10 (-24 to -2)	-40 (-59 to -28)	-18 (-38 to -4)	-13 (-29 to -4)	-6 (-14 to -0)	-18 (-40 to -4)	-8 (-17 to -1)		+2 (-4 to +7)	+2 (-1 to +6)	+2 (+0 to +6)
	HPV16/18 AND ≥ASCUS	-139 (-229 to -81)	-135 (-189 to -94)	-109 (-233 to -48)	-112 (-173 to -70)	-107 (-155 to -70)	-84 (-126 to -52)	-80 (-113 to -55)	-66 (-119 to -32)	-47 (-107 to -14)	-74 (-134 to -38)	-59 (-108 to -30)	-27 (-49 to -10)	-36 (-64 to -17)	-24 (-64 to -1)	-14 (-30 to +0)	-44 (-64 to -28)	-21 (-41 to -7)	-17 (-34 to -2)	-10 (-21 to +4)	-22 (-43 to -6)	-12 (-23 to +1)	-4 (-14 to +10)		+0 (-5 to +6)	+0 (-5 to +6)
	≥HSIL	-145 (-235 to -86)	-141 (-195 to -100)	-114 (-238 to -53)	-117 (-179 to -75)	-112 (-160 to -76)	-89 (-131 to -59)	-85 (-117 to -62)	-71 (-125 to -39)	-52 (-112 to -21)	-79 (-141 to -42)	-64 (-115 to -34)	-32 (-53 to -19)	-41 (-70 to -23)	-29 (-69 to -9)	-19 (-34 to -10)	-49 (-69 to -36)	-26 (-46 to -14)	-22 (-38 to -13)	-15 (-24 to -8)	-27 (-49 to -13)	-17 (-26 to -11)	-8 (-16 to -3)	-4 (-18 to +2)		+0 (-4 to +4)
	HC2 AND ≥HSIL	-145 (-235 to -85)	-140 (-194 to -100)	-114 (-239 to -53)	-117 (-180 to -75)	-112 (-160 to -76)	-89 (-131 to -59)	-85 (-117 to -62)	-71 (-125 to -39)	-52 (-113 to -21)	-79 (-141 to -42)	-64 (-115 to -35)	-32 (-53 to -20)	-41 (-70 to -23)	-29 (-69 to -9)	-19 (-34 to -10)	-49 (-69 to -35)	-26 (-47 to -14)	-22 (-38 to -13)	-15 (-24 to -8)	-27 (-49 to -13)	-17 (-26 to -10)	-9 (-16 to -3)	-5 (-18 to +3)	-0 (-4 to +4)	

*Above the diagonal line (formed by gray cells) represents the number of the difference in (Δ) FNs (95% CrI) and below the diagonal line represents Δ FPs (95% CrI). For Δ FPs, the rows and columns respectively represent the index (the test of interest) and comparator (the test in comparison) tests or combination algorithms. For Δ FNs, the columns and rows respectively represent the index and comparator tests or combination algorithms. Results are based on a healthy screening population of 1,000 women in which 20 are CIN2+ (2%).

ASCH, atypical squamous cells cannot exclude high-grade lesion; ASCUS, atypical squamous cells of undetermined significance; CrI, credible interval; FN, false negative; FP, false positive; HC2, Hybrid Capture 2; HPV16/18(/45), genotyping for HPV types 16 or 18 (or 45); HSIL, high-grade squamous intraepithelial lesion; LBC, liquid-based cytology; LSIL, low-grade squamous intraepithelial lesion; mRNA, messenger ribonucleic acid; PCR, polymerase chain reaction.

Table S7. Comparative Accuracy of Standalone Tests.*

Index and comparator tests or combination algorithms		Index (for specificity) and comparator (for sensitivity) tests or combination algorithms							
		PCR	HC2	mRNA	≥ASCUS	≥ASCH	HPV16/18	≥LSIL	≥HSIL
Index (for sensitivity) and comparator (for specificity) tests or combination algorithms	PCR		0.94 (0.87–1.02) [0.055]	0.93 (0.73–1.04) [0.104]	0.65 (0.54–0.75) [<0.001]	0.61 (0.48–0.72) [<0.001]	0.56 (0.34–0.76) [<0.001]	0.56 (0.42–0.67) [<0.001]	0.37 (0.23–0.53) [<0.001]
	HC2	1.04 (0.99–1.11) [0.076]		0.99 (0.79–1.10) [0.423]	0.69 (0.58–0.79) [<0.001]	0.64 (0.52–0.75) [<0.001]	0.59 (0.36–0.81) [<0.001]	0.59 (0.46–0.70) [<0.001]	0.39 (0.25–0.56) [<0.001]
	mRNA	1.05 (0.99–1.13) [0.052]	1.02 (0.96–1.06) [0.260]		0.71 (0.58–0.89) [0.005]	0.66 (0.51–0.84) [0.002]	0.60 (0.37–0.86) [0.002]	0.60 (0.45–0.78) [<0.001]	0.40 (0.25–0.60) [<0.001]
	≥ASCUS	1.08 (1.03–1.15) [<0.001]	1.04 (1.02–1.07) [<0.001]	1.02 (0.99–1.08) [0.096]		0.94 (0.79–1.00) [<0.001]	0.85 (0.53–1.18) [0.192]	0.86 (0.69–0.97) [<0.001]	0.57 (0.37–0.79) [<0.001]
	≥ASCH	1.11 (1.06–1.19) [<0.001]	1.07 (1.05–1.11) [<0.001]	1.05 (1.02–1.12) [0.001]	1.03 (1.01–1.05) [<0.001]		0.92 (0.57–1.31) [0.329]	0.93 (0.75–1.00) [<0.001]	0.61 (0.40–0.86) [<0.001]
	HPV16/18	1.11 (1.06–1.18) [<0.001]	1.06 (1.04–1.10) [<0.001]	1.05 (1.01–1.11) [0.004]	1.02 (1.00–1.04) [0.015]	1.00 (0.97–1.01) [0.297]		1.00 (0.68–1.62) [0.495]	0.67 (0.39–1.18) [0.076]
	≥LSIL	1.12 (1.07–1.20) [<0.001]	1.08 (1.05–1.11) [<0.001]	1.06 (1.02–1.13) [<0.001]	1.03 (1.02–1.05) [<0.001]	1.00 (1.00–1.02) [<0.001]	1.01 (1.00–1.03) [0.095]		0.67 (0.45–0.94) [<0.001]
	≥HSIL	1.14 (1.08–1.23) [<0.001]	1.10 (1.07–1.14) [<0.001]	1.08 (1.04–1.15) [<0.001]	1.05 (1.04–1.08) [<0.001]	1.02 (1.01–1.04) [<0.001]	1.03 (1.01–1.05) [<0.001]	1.02 (1.01–1.03) [<0.001]	

*Above the diagonal line (formed by gray cells) represents relative sensitivity (95% CrI) [probability that relative sensitivity is ≥1] and below the diagonal line represents relative specificity (95% CrI) [probability that relative specificity is ≤1]. For relative sensitivity, the rows and columns, respectively, represent the index (the test of interest) and comparator (the test in comparison) tests or combination algorithms. For relative sensitivity, the columns and rows, respectively, represent the index and comparator tests or combination algorithms.

ASCH, atypical squamous cells cannot exclude high-grade lesion; ASCUS, atypical squamous cells of undetermined significance; CrI, credible interval; HC2, Hybrid Capture 2; HPV16/18(/45), genotyping for HPV types 16 or 18 (or 45); HSIL, high-grade squamous intraepithelial lesion; LBC, liquid-based cytology; LSIL, low-grade squamous intraepithelial lesion; mRNA, messenger ribonucleic acid; PCR, polymerase chain reaction.

Table S8. Comparative Accuracy of HC2-based Combinations.*

Index and comparator tests or combination algorithms		Index (for specificity) and comparator (for sensitivity) tests or combination algorithms									
		HC2 OR ≥ASCUS	HC2 OR ≥LSIL	HC2 OR ≥HSIL	HC2	≥LSIL OR [HC2 AND ASCUS]	≥HSIL OR [HC2 AND ≥ASCUS]	≥ASCUS	HC2 AND ≥ASCUS	HC2 AND ≥LSIL	HC2 AND ≥HSIL
Index (for sensitivity) and comparator (for specificity) tests or combination algorithms	HC2 OR ≥ASCUS		0.96 (0.91–0.99) [<0.001]	0.94 (0.86–0.98) [<0.001]	0.91 (0.85–0.96) [<0.001]	0.72 (0.57–0.85) [<0.001]	0.63 (0.46–0.79) [<0.001]	0.63 (0.52–0.73) [<0.001]	0.55 (0.40–0.69) [<0.001]	0.48 (0.32–0.63) [<0.001]	0.36 (0.19–0.53) [<0.001]
	HC2 OR ≥LSIL	1.03 (1.00–1.09) [<0.001]		0.98 (0.91–1.00) [<0.001]	0.95 (0.89–1.01) [0.056]	0.76 (0.60–0.88) [<0.001]	0.66 (0.49–0.82) [<0.001]	0.66 (0.55–0.76) [<0.001]	0.58 (0.43–0.71) [<0.001]	0.50 (0.33–0.65) [<0.001]	0.37 (0.20–0.55) [<0.001]
	HC2 OR ≥HSIL	1.06 (1.02–1.12) [<0.001]	1.02 (1.00–1.07) [<0.001]		0.98 (0.91–1.06) [0.266]	0.78 (0.62–0.91) [<0.001]	0.68 (0.51–0.84) [<0.001]	0.68 (0.57–0.78) [<0.001]	0.59 (0.44–0.73) [<0.001]	0.51 (0.35–0.66) [<0.001]	0.38 (0.21–0.57) [<0.001]
	HC2	1.07 (1.03–1.12) [<0.001]	1.03 (0.99–1.08) [0.046]	1.00 (0.97–1.04) [0.396]		0.79 (0.63–0.93) [0.001]	0.69 (0.51–0.86) [<0.001]	0.69 (0.58–0.79) [<0.001]	0.61 (0.45–0.75) [<0.001]	0.52 (0.35–0.68) [<0.001]	0.39 (0.21–0.58) [<0.001]
	≥LSIL OR [HC2 AND ASCUS]	1.13 (1.08–1.20) [<0.001]	1.09 (1.05–1.15) [<0.001]	1.06 (1.03–1.11) [<0.001]	1.06 (1.03–1.09) [<0.001]		0.89 (0.68–0.99) [<0.001]	0.88 (0.71–1.10) [0.113]	0.77 (0.59–0.93) [<0.001]	0.67 (0.46–0.85) [<0.001]	0.50 (0.27–0.72) [<0.001]
	≥HSIL OR [HC2 AND ≥ASCUS]	1.15 (1.10–1.22) [<0.001]	1.11 (1.07–1.17) [<0.001]	1.08 (1.04–1.13) [<0.001]	1.07 (1.05–1.11) [<0.001]	1.01 (1.00–1.03) [<0.001]		1.00 (0.79–1.32) [0.491]	0.89 (0.68–0.99) [<0.001]	0.76 (0.53–0.95) [<0.001]	0.57 (0.32–0.83) [<0.001]
	≥ASCUS	1.11 (1.07–1.17) [<0.001]	1.07 (1.04–1.12) [<0.001]	1.04 (1.01–1.09) [0.001]	1.04 (1.02–1.07) [<0.001]	0.98 (0.96–1.00) [0.036]	0.97 (0.95–0.98) [<0.001]		0.88 (0.66–1.09) [0.126]	0.76 (0.52–0.99) [0.021]	0.57 (0.32–0.83) [0.001]
	HC2 AND ≥ASCUS	1.15 (1.10–1.23) [<0.001]	1.11 (1.07–1.17) [<0.001]	1.08 (1.05–1.13) [<0.001]	1.08 (1.05–1.12) [<0.001]	1.02 (1.01–1.04) [<0.001]	1.00 (1.00–1.02) [<0.001]	1.04 (1.02–1.06) [<0.001]		0.89 (0.64–0.99) [<0.001]	0.65 (0.37–0.91) [<0.001]
	HC2 AND ≥LSIL	1.16 (1.11–1.24) [<0.001]	1.12 (1.08–1.18) [<0.001]	1.09 (1.06–1.14) [<0.001]	1.09 (1.06–1.12) [<0.001]	1.03 (1.01–1.05) [<0.001]	1.01 (1.00–1.03) [<0.001]	1.04 (1.03–1.07) [<0.001]	1.01 (1.00–1.01) [<0.001]		0.77 (0.44–0.98) [<0.001]

	HC2 AND ≥HSIL	1.17 (1.11–1.25) [<0.001]	1.13 (1.08–1.20) [<0.001]	1.10 (1.06–1.16) [<0.001]	1.10 (1.07–1.14) [<0.001]	1.03 (1.02–1.06) [<0.001]	1.02 (1.01–1.04) [<0.001]	1.05 (1.04–1.08) [<0.001]	1.02 (1.01–1.03) [<0.001]	1.01 (1.00–1.02) [<0.001]	

*Above the diagonal line (formed by gray cells) represents relative sensitivity (95% CrI) [probability that relative sensitivity is ≥1] and below the diagonal line represents relative specificity (95% CrI) [probability that relative specificity is ≤1]. For relative sensitivity, the rows and columns, respectively, represent the index (the test of interest) and comparator (the test in comparison) tests or combination algorithms. For relative sensitivity, the columns and rows, respectively, represent the index and comparator tests or combination algorithms.

ASCUS, atypical squamous cells of undetermined significance; CrI, credible interval; HC2, Hybrid Capture 2; HSIL, high-grade squamous intraepithelial lesion; LSIL, low-grade squamous intraepithelial lesion.

Table S9. Comparative Accuracy of PCR-based Combinations.*

Index and comparator tests or combination algorithms		Index (for specificity) and comparator (for sensitivity) tests or combination algorithms								
		PCR OR ≥ASCUS	PCR	≥LSIL OR [PCR AND ASCUS]	HPV16/18 OR ≥ASCUS	HPV16/18 OR [PCR AND ≥ASCUS]	≥ASCUS	PCR AND ≥ASCUS	HPV16/18	HPV16/18 AND ≥ASCUS
Index (for sensitivity) and comparator (for specificity) tests or combination algorithms	PCR OR ≥ASCUS		0.94 (0.87–0.98) [<0.001]	0.84 (0.67–0.94) [<0.001]	0.75 (0.57–0.88) [<0.001]	0.70 (0.52–0.83) [<0.001]	0.61 (0.50–0.71) [<0.001]	0.58 (0.38–0.75) [<0.001]	0.52 (0.32–0.72) [<0.001]	0.37 (0.13–0.63) [<0.001]
	PCR	1.03 (0.96–1.14) [0.211]		0.89 (0.72–1.01) [0.043]	0.80 (0.62–0.93) [0.001]	0.74 (0.56–0.89) [<0.001]	0.65 (0.54–0.75) [<0.001]	0.62 (0.41–0.79) [<0.001]	0.56 (0.34–0.76) [<0.001]	0.40 (0.14–0.66) [<0.001]
	≥LSIL OR [PCR AND ASCUS]	1.08 (1.01–1.19) [<0.001]	1.04 (0.97–1.12) [0.098]		0.90 (0.73–0.99) [<0.001]	0.84 (0.65–0.97) [<0.001]	0.73 (0.59–0.92) [0.004]	0.70 (0.47–0.88) [<0.001]	0.63 (0.38–0.88) [0.002]	0.45 (0.16–0.74) [<0.001]
	HPV16/18 OR ≥ASCUS	1.10 (1.03–1.22) [<0.001]	1.06 (1.00–1.14) [0.027]	1.01 (1.00–1.06) [<0.001]		0.95 (0.78–1.00) [<0.001]	0.82 (0.67–1.06) [0.054]	0.78 (0.55–0.95) [<0.001]	0.70 (0.44–0.99) [0.022]	0.50 (0.19–0.81) [<0.001]
	HPV16/18 OR [PCR AND ≥ASCUS]	1.12 (1.06–1.25) [<0.001]	1.09 (1.04–1.16) [<0.001]	1.04 (1.01–1.11) [<0.001]	1.02 (1.00–1.08) [<0.001]		0.88 (0.71–1.16) [0.157]	0.84 (0.60–0.99) [<0.001]	0.75 (0.47–1.07) [0.063]	0.54 (0.21–0.86) [<0.001]
	≥ASCUS	1.11 (1.04–1.24) [<0.001]	1.08 (1.03–1.15) [<0.001]	1.03 (0.99–1.10) [0.056]	1.02 (0.99–1.07) [0.185]	0.99 (0.97–1.02) [0.231]		0.95 (0.64–1.24) [0.361]	0.85 (0.53–1.18) [0.192]	0.61 (0.22–1.02) [0.032]
	PCR AND ≥ASCUS	1.14 (1.07–1.27) [<0.001]	1.11 (1.06–1.18) [<0.001]	1.06 (1.02–1.13) [<0.001]	1.04 (1.01–1.10) [<0.001]	1.01 (1.00–1.04) [<0.001]	1.02 (1.01–1.04) [0.008]		0.90 (0.56–1.40) [0.317]	0.66 (0.25–0.98) [<0.001]
	HPV16/18	1.14 (1.07–1.27) [<0.001]	1.11 (1.06–1.18) [<0.001]	1.06 (1.02–1.13) [0.001]	1.04 (1.01–1.10) [0.005]	1.01 (0.99–1.04) [0.101]	1.02 (1.00–1.04) [0.015]	1.00 (0.98–1.02) [0.489]		0.72 (0.27–1.34) [0.153]
	HPV16/18 AND ≥ASCUS	1.17 (1.09–1.31) [<0.001]	1.13 (1.08–1.22) [<0.001]	1.08 (1.04–1.16) [<0.001]	1.06 (1.03–1.13) [<0.001]	1.04 (1.02–1.07) [<0.001]	1.05 (1.03–1.07) [<0.001]	1.02 (1.01–1.04) [<0.001]	1.02 (1.01–1.05) [0.004]	

*Above the diagonal line (formed by gray cells) represents relative sensitivity (95% CrI) [probability that relative sensitivity is ≥1] and below the diagonal line represents relative specificity (95% CrI) [probability that relative specificity is ≤1]. For relative sensitivity, the rows and columns, respectively, represent the index (the test of interest) and comparator (the test in comparison) tests or combination algorithms. For relative sensitivity, the columns and rows, respectively, represent the index and comparator tests or combination algorithms.

ASCUS, atypical squamous cells of undetermined significance; CrI, credible interval; HPV16/18, genotyping for HPV types 16 or 18; LSIL, low-grade squamous intraepithelial lesion; PCR, polymerase chain reaction.

Table S10. Comparative Accuracy of mRNA-based Combinations.*

Index and comparator tests or combination algorithms		Index (for specificity) and comparator (for sensitivity) tests or combination algorithms				
		mRNA OR ≥ASCUS	mRNA	HPV16/18/45 OR [mRNA AND ≥ASCUS]	mRNA AND ≥ASCUS	≥ASCUS
Index (for sensitivity) and comparator (for specificity) tests or combination algorithms	mRNA OR ≥ASCUS		0.92 (0.73–1.05) [0.090]	0.90 (0.49–1.00) [<0.001]	0.70 (0.20–0.96) [<0.001]	0.64 (0.53–0.77) [<0.001]
	mRNA	1.05 (0.97–1.22) [0.132]		0.97 (0.53–1.24) [0.405]	0.76 (0.23–1.12) [0.112]	0.71 (0.58–0.89) [0.005]
	HPV16/18/45 OR [mRNA AND ≥ASCUS]	1.07 (1.00–1.24) [<0.001]	1.02 (0.96–1.08) [0.231]		0.83 (0.28–0.99) [<0.001]	0.73 (0.58–1.33) [0.089]
	mRNA AND ≥ASCUS	1.10 (1.02–1.28) [<0.001]	1.05 (1.00–1.11) [0.029]	1.02 (1.00–1.08) [<0.001]		0.93 (0.63–3.18) [0.419]
	≥ASCUS	1.07 (1.01–1.25) [0.014]	1.02 (0.99–1.08) [0.096]	1.00 (0.97–1.07) [0.446]	0.98 (0.95–1.02) [0.123]	

*Above the diagonal line (formed by gray cells) represents relative sensitivity (95% CrI) [probability that relative sensitivity is ≥1] and below the diagonal line represents relative specificity (95% CrI) [probability that relative specificity is ≤1]. For relative sensitivity, the rows and columns, respectively, represent the index (the test of interest) and comparator (the test in comparison) tests or combination algorithms. For relative sensitivity, the columns and rows, respectively, represent the index and comparator tests or combination algorithms.

ASCUS, atypical squamous cells of undetermined significance; CrI, credible interval; HPV16/18/45, genotyping for HPV types 16, 18, or 45; mRNA, messenger ribonucleic acid.

Table S11. The GRADE Summary of Findings Table for Standalone Tests and Combination Algorithms.

Screening strategies	Study (women), *n*	TP (CrI)	FN (CrI)	FP (CrI)	TN (CrI)	Limitations	Indirectness	Inconsistency	Imprecision	Reporting bias	Certainty
**PCR**	6 (32,720)	19 (17–20)	1 (0–3)	124 (80–187)	856 (793–900)	Not serious	Serious[a]	Not serious	Not serious	Not assessable	Low
**HC2**	20 (109,245)	18 (16–19)	2 (1–4)	92 (67–125)	888 (855–913)	Not serious	Serious[a]	Serious[b]	Not serious	Not serious	Low
**mRNA**	3 (18,926)	17 (14–19)	3 (1–6)	78 (44–133)	902 (847–936)	Not serious	Serious[a]	Not serious	Serious[c]	Not assessable	Very low
**HPV16/18/45 OR [mRNA AND ≥ASCUS]**	1 (5,046)	17 (9–19)	3 (1–11)	58 (27–119)	922 (861–953)	Not serious	Serious[a]	Serious[b]	Serious[c]	Serious[d]	Very low
**≥LSIL OR [PCR AND ≥ASCUS]**	1 (11,064)	17 (13–19)	3 (1–7)	85 (48–148)	895 (832–932)	Not serious	Serious[a]	Serious[b]	Serious[c]	Serious[d]	Very low
**≥LSIL OR [HC2 AND ASCUS]**	5 (25,639)	14 (11–17)	6 (3–9)	39 (25–61)	941 (919–955)	Not serious	Serious[a]	Not serious	Serious[c]	Serious[d]	Very low
**HPV16/18 OR [PCR AND ≥ASCUS]**	3 (23,695)	14 (10–17)	6 (3–10)	47 (29–78)	933 (902–951)	Not serious	Serious[a]	Not serious	Serious[c]	Serious[d]	Very low
**≥ASCUS**	20 (153,178)	12 (10–14)	8 (6–10)	56 (41–77)	924 (903–939)	Not serious	Serious[a]	Serious[b]	Serious[c]	Not serious	Very low

[a]Detection of CIN2+ is an intermediate outcome.

[b]By visual assessment of the ROC plots. Also considered as serious in case there is only a single data point.

[c]Based on wide CrIs.

[d]Based on the greater number of studies that assessed its component hrHPV testing.

ASCUS, atypical squamous cells of undetermined significance; CIN2+, CrI, cervical intraepithelial neoplasia grade 2 or higher grades; 95% credible interval; FN, false negative; FP, false positive; HC2, Hybrid Capture 2; HPV16/18(/45), genotyping for HPV types 16 or 18 (or 45); HSIL, high-grade squamous intraepithelial lesion; LBC, liquid-based cytology; LSIL, low-grade

squamous intraepithelial lesion; mRNA, messenger ribonucleic acid; PCR, polymerase chain reaction; ROC, receiver operating characteristic; TN, true negative; TP, true positive.

Table S12. Sensitivity Analysis based on Alternative Models.

Model (DIC)	Test-specific correlation parameters with threshold constraints (1290.9)			A common between-observation correlation parameter with threshold constraints (1291.5)			Test-specific correlation parameters without threshold constraints (1294.2)			A common between-observation correlation parameter without threshold constraints (1295.2)		
Screening strategies	Estimates (95% CrI)	Ranking (95% CrI)	Best P	Estimates (95% CrI)	Ranking (95% CrI)	Best P	Estimates (95% CrI)	Ranking (95% CrI)	Best P	Estimates (95% CrI)	Ranking (95% CrI)	Best P
Sensitivity												
HC2	0.884 (0.821–0.926)	8 (5–10)	0	0.883 (0.816–0.926)	8 (5–10)	0	0.882 (0.818–0.925)	8 (5–10)	0	0.881 (0.815–0.924)	8 (5–10)	0
mRNA	0.872 (0.690–0.955)	8 (4–13)	0	0.872 (0.691–0.954)	8 (4–13)	0	0.866 (0.673–0.954)	8 (4–13)	0	0.869 (0.680–0.952)	8 (4–13)	0
HPV16/18	0.520 (0.317–0.719)	20 (13–24)	0	0.519 (0.312–0.719)	19 (12–24)	0	0.507 (0.295–0.711)	20 (12–24)	0	0.513 (0.306–0.714)	19 (13–24)	0
PCR	0.941 (0.872–0.976)	4 (2–8)	0	0.938 (0.868–0.974)	4 (2–8)	0	0.942 (0.872–0.976)	4 (2–8)	0	0.941 (0.868–0.974)	4 (2–8)	0
mRNA AND ≥ASCUS	0.657 (0.190–0.921)	14 (7–25)	0	0.644 (0.177–0.919)	14 (7–25)	0	0.830 (0.281–0.983)	9 (2–24)	0	0.833 (0.298–0.985)	9 (2–24)	0
HPV16/18/45 OR [mRNA AND ≥ASCUS]	0.844 (0.456–0.966)	9 (4–20)	0	0.848 (0.476–0.967)	9 (3–20)	0	0.804 (0.258–0.980)	10 (2–25)	0	0.809 (0.290–0.979)	10 (2–24)	0
mRNA OR ≥ASCUS	0.957 (0.832–0.991)	3 (2–8)	0	0.957 (0.836–0.991)	3 (2–8)	0	0.944 (0.760–0.989)	4 (2–11)	0	0.940 (0.742–0.988)	4 (2–11)	0
≥HSIL	0.346 (0.216–0.497)	24 (21–25)	0	0.346 (0.210–0.499)	24 (21–25)	0	0.356 (0.218–0.521)	23 (19–25)	0	0.357 (0.219–0.522)	23 (19–25)	0
≥LSIL	0.520 (0.392–0.631)	20 (16–23)	0	0.516 (0.388–0.628)	20 (16–23)	0	0.579 (0.435–0.710)	17 (12–21)	0	0.580 (0.433–0.709)	17 (12–21)	0
≥ASCH	0.568 (0.445–0.675)	17 (14–21)	0	0.565 (0.439–0.670)	18 (14–21)	0	0.536 (0.272–0.770)	18 (11–25)	0	0.525 (0.269–0.767)	19 (11–25)	0
≥ASCUS	0.611 (0.499–0.710)	15 (12–18)	0	0.607 (0.494–0.702)	15 (12–18)	0	0.579 (0.457–0.688)	17 (13–21)	0	0.577 (0.456–0.685)	17 (13–21)	0
HPV16/18 AND ≥ASCUS	0.370 (0.133–0.626)	23 (16–25)	0	0.375 (0.136–0.628)	23 (16–25)	0	0.336 (0.098–0.709)	24 (13–25)	0	0.354 (0.106–0.718)	24 (12–25)	0
PCR AND ≥ASCUS	0.578 (0.380–0.746)	17 (13–23)	0	0.570 (0.378–0.749)	17 (13–23)	0	0.599 (0.373–0.791)	16 (11–23)	0	0.611 (0.391–0.790)	15 (11–23)	0
HPV16/18 OR [PCR AND ≥ASCUS]	0.696 (0.515–0.834)	13 (11–18)	0	0.694 (0.507–0.835)	13 (11–18)	0	0.773 (0.554–0.903)	11 (7–17)	0	0.783 (0.578–0.906)	11 (7–16)	0
HPV16/18 OR ≥ASCUS	0.748 (0.570–0.876)	11 (9–15)	0	0.745 (0.562–0.874)	11 (9–15)	0	0.644 (0.273–0.895)	14 (7–24)	0	0.659 (0.301–0.900)	14 (7–24)	0
≥LSIL OR [PCR AND ≥ASCUS]	0.839 (0.670–0.943)	9 (4–12)	0	0.848 (0.667–0.946)	9 (4–12)	0	0.699 (0.345–0.905)	12 (7–23)	0	0.677 (0.334–0.898)	13 (7–24)	0
PCR OR ≥ASCUS	1.000 (0.994–1.000)	1 (1–1)	1	1.000 (0.993–1.000)	1 (1–1)	1	1.000 (0.995–1.000)	1 (1–1)	1	1.000 (0.994–1.000)	1 (1–1)	1

HC2 AND ≥HSIL	0.345 (0.183–0.519)	24 (21–25)	0	0.356 (0.193–0.528)	24 (21–25)	0	0.380 (0.191–0.617)	23 (15–25)	0	0.407 (0.214–0.640)	22 (15–25)	0
HC2 AND ≥LSIL	0.461 (0.305–0.611)	22 (17–24)	0	0.464 (0.304–0.606)	22 (17–24)	0	0.521 (0.309–0.723)	19 (12–24)	0	0.526 (0.319–0.726)	19 (12–24)	0
HC2 AND ≥ASCUS	0.534 (0.384–0.668)	19 (14–22)	0	0.529 (0.380–0.665)	19 (14–22)	0	0.545 (0.378–0.699)	18 (12–23)	0	0.538 (0.371–0.693)	18 (13–23)	0
≥HSIL OR [HC2 AND ≥ASCUS]	0.612 (0.445–0.765)	15 (11–20)	0	0.613 (0.446–0.767)	15 (11–20)	0	0.410 (0.105–0.810)	22 (10–25)	0	0.425 (0.111–0.816)	22 (10–25)	0
≥LSIL OR [HC2 AND ASCUS]	0.699 (0.542–0.826)	13 (9–17)	0	0.702 (0.542–0.828)	13 (9–17)	0	0.671 (0.486–0.814)	13 (9–20)	0	0.674 (0.495–0.814)	13 (9–19)	0
HC2 OR ≥HSIL	0.904 (0.824–0.950)	7 (4–10)	0	0.899 (0.814–0.945)	7 (4–10)	0	0.929 (0.846–0.969)	5 (3–9)	0	0.925 (0.844–0.966)	5 (3–9)	0
HC2 OR ≥LSIL	0.929 (0.870–0.963)	5 (3–8)	0	0.924 (0.861–0.959)	5 (3–8)	0	0.918 (0.836–0.962)	6 (3–9)	0	0.913 (0.831–0.959)	6 (3–10)	0
HC2 OR ≥ASCUS	0.968 (0.937–0.984)	2 (2–4)	0	0.966 (0.934–0.983)	2 (2–4)	0	0.966 (0.932–0.984)	2 (2–5)	0	0.964 (0.929–0.982)	2 (2–5)	0
Specificity												
HC2	0.906 (0.872–0.932)	19 (16–22)	0	0.907 (0.874–0.932)	19 (16–22)	0	0.907 (0.874–0.933)	19 (16–21)	0	0.907 (0.873–0.932)	19 (16–21)	0
mRNA	0.921 (0.864–0.955)	17 (13–22)	0	0.922 (0.867–0.956)	17 (13–22)	0	0.921 (0.865–0.954)	17 (14–22)	0	0.920 (0.864–0.954)	17 (14–22)	0
HPV16/18	0.966 (0.942–0.980)	10 (5–13)	0	0.966 (0.943–0.980)	10 (5–14)	0	0.965 (0.941–0.979)	10 (6–14)	0	0.965 (0.939–0.979)	11 (6–15)	0
PCR	0.874 (0.810–0.918)	22 (18–25)	0	0.876 (0.811–0.919)	22 (18–25)	0	0.873 (0.808–0.917)	22 (18–25)	0	0.872 (0.807–0.917)	22 (18–25)	0
mRNA AND ≥ASCUS	0.963 (0.922–0.985)	11 (4–16)	0	0.967 (0.928–0.986)	10 (4–15)	0	0.956 (0.893–0.983)	13 (5–20)	0	0.956 (0.892–0.983)	13 (5–20)	0
HPV16/18/45 OR [mRNA AND ≥ASCUS]	0.941 (0.878–0.972)	15 (10–21)	0	0.942 (0.879–0.973)	15 (9–21)	0	0.944 (0.869–0.978)	15 (7–22)	0	0.944 (0.867–0.978)	15 (7–22)	0
mRNA OR ≥ASCUS	0.877 (0.750–0.940)	22 (16–25)	0	0.874 (0.741–0.938)	22 (16–25)	0	0.887 (0.766–0.951)	21 (14–25)	0	0.888 (0.763–0.951)	21 (14–25)	0
≥HSIL	0.994 (0.990–0.996)	2 (1–3)	0.43	0.994 (0.990–0.996)	2 (1–3)	0.39	0.993 (0.989–0.996)	2 (1–3)	0.47	0.993 (0.989–0.996)	2 (1–3)	0.47
≥LSIL	0.976 (0.966–0.983)	6 (5–9)	0	0.976 (0.966–0.984)	6 (5–9)	0	0.973 (0.960–0.981)	8 (6–11)	0	0.972 (0.960–0.981)	8 (6–12)	0
≥ASCH	0.971 (0.953–0.981)	8 (6–12)	0	0.971 (0.954–0.981)	8 (6–13)	0	0.980 (0.960–0.990)	6 (3–12)	0	0.980 (0.959–0.990)	6 (3–12)	0
≥ASCUS	0.943 (0.922–0.959)	14 (12–17)	0	0.944 (0.923–0.959)	14 (12–17)	0	0.946 (0.924–0.960)	15 (12–17)	0	0.945 (0.923–0.960)	15 (12–17)	0
HPV16/18 AND ≥ASCUS	0.989 (0.975–0.995)	3 (1–6)	0.07	0.989 (0.976–0.996)	3 (1–6)	0.07	0.987 (0.967–0.995)	4 (1–9)	0.05	0.986 (0.964–0.995)	4 (1–10)	0.05
PCR AND ≥ASCUS	0.966 (0.945–0.980)	10 (6–13)	0	0.967 (0.947–0.980)	10 (5–13)	0	0.966 (0.941–0.980)	10 (6–14)	0	0.966 (0.941–0.980)	10 (6–14)	0
HPV16/18 OR [PCR AND ≥ASCUS]	0.952 (0.920–0.971)	13 (10–16)	0	0.954 (0.924–0.972)	13 (10–15)	0	0.945 (0.905–0.969)	15 (10–18)	0	0.945 (0.904–0.969)	15 (10–18)	0
HPV16/18 OR ≥ASCUS	0.928 (0.875–0.959)	16 (13–20)	0	0.926 (0.875–0.957)	16 (13–20)	0	0.918 (0.812–0.967)	18 (10–25)	0	0.916 (0.803–0.966)	18 (11–25)	0

≥LSIL OR [PCR AND ≥ASCUS]	0.913 (0.849–0.951)	18 (15–23)	0	0.910 (0.850–0.948)	18 (15–23)	0	0.956 (0.900–0.981)	13 (6–19)	0	0.957 (0.900–0.982)	12 (5–19)	0
PCR OR ≥ASCUS	0.846 (0.753–0.907)	24 (20–25)	0	0.847 (0.756–0.908)	24 (20–25)	0	0.850 (0.757–0.911)	24 (19–25)	0	0.851 (0.758–0.913)	24 (19–25)	0
HC2 AND ≥HSIL	0.994 (0.989–0.997)	2 (1–3)	0.5	0.994 (0.989–0.997)	1 (1–3)	0.54	0.993 (0.988–0.996)	2 (1–3)	0.46	0.993 (0.988–0.996)	2 (1–3)	0.46
HC2 AND ≥LSIL	0.985 (0.976–0.991)	4 (3–5)	0	0.985 (0.976–0.991)	4 (3–5)	0	0.983 (0.969–0.990)	5 (3–8)	0	0.983 (0.969–0.990)	5 (3–8)	0
HC2 AND ≥ASCUS	0.979 (0.968–0.986)	5 (4–8)	0	0.979 (0.968–0.985)	5 (4–8)	0	0.977 (0.966–0.985)	7 (4–10)	0	0.977 (0.965–0.985)	7 (4–10)	0
≥HSIL OR [HC2 AND ≥ASCUS]	0.974 (0.958–0.984)	7 (6–11)	0	0.974 (0.958–0.983)	8 (6–11)	0	0.985 (0.965–0.994)	4 (2–10)	0.02	0.985 (0.964–0.994)	4 (2–10)	0.02
≥LSIL OR [HC2 AND ASCUS]	0.961 (0.938–0.975)	11 (8–15)	0	0.960 (0.938–0.974)	12 (9–15)	0	0.962 (0.940–0.976)	11 (8–15)	0	0.962 (0.939–0.976)	11 (8–15)	0
HC2 OR ≥HSIL	0.902 (0.858–0.935)	19 (16–22)	0	0.904 (0.860–0.936)	19 (16–22)	0	0.891 (0.835–0.929)	21 (17–24)	0	0.890 (0.834–0.928)	21 (17–24)	0
HC2 OR ≥LSIL	0.879 (0.828–0.917)	22 (19–24)	0	0.880 (0.829–0.917)	22 (19–24)	0	0.883 (0.825–0.925)	21 (17–25)	0	0.883 (0.824–0.924)	21 (17–25)	0
HC2 OR ≥ASCUS	0.850 (0.794–0.893)	24 (22–25)	0	0.851 (0.795–0.893)	24 (22–25)	0	0.854 (0.796–0.897)	24 (21–25)	0	0.853 (0.795–0.896)	24 (21–25)	0

ASCH, atypical squamous cells cannot exclude high-grade lesion; ASCUS, atypical squamous cells of undetermined significance; CrI, credible interval; DIC, deviance information criterion; FN, false negative; FP, false positive; HC2, Hybrid Capture 2; HPV16/18(/45), genotyping for HPV types 16 or 18 (or 45); HSIL, high-grade squamous intraepithelial lesion; LBC, liquid-based cytology; LSIL, low-grade squamous intraepithelial lesion; mRNA, messenger ribonucleic acid; PCR, polymerase chain reaction.

ONLINE-ONLY SUPPLEMENTARY FIGURES

Figure S1

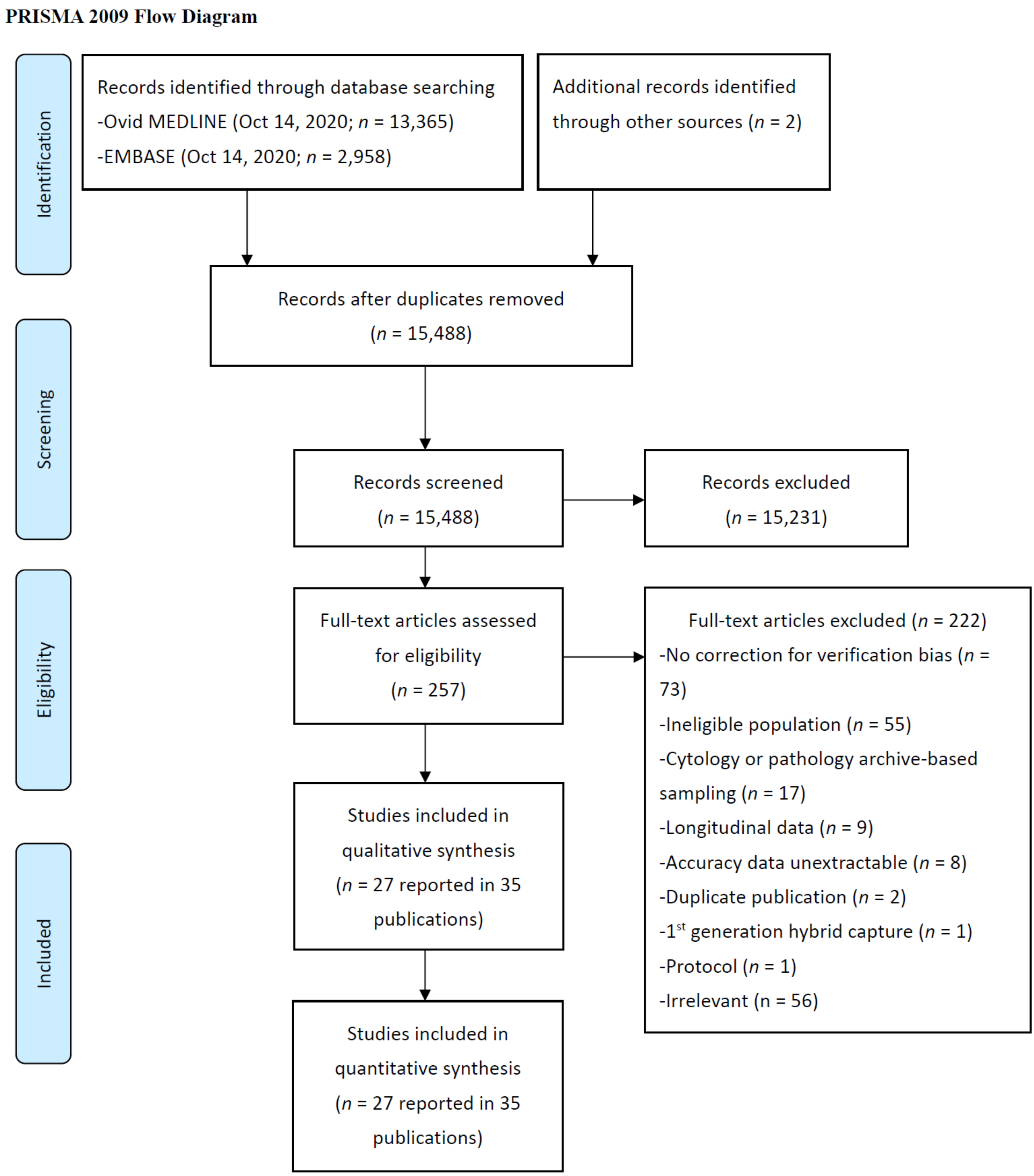

Figure S1. PRISMA Flow Diagram

Figure S2

	Risk of Bias				Concerns Regarding Applicability		
Study, characterized by design	DOMAIN 1 Patient Selection	DOMAIN 2 Index Test	DOMAIN 3 Reference Standard	DOMAIN 4 Flow and Timing	DOMAIN 1 Patient Selection	DOMAIN 2 Index Test	DOMAIN 3 Reference Standard
Histology-based Studies							
Belinson 2001[18]	Low	Low	Low	Low	High[a,b]	Low	Low
Cárdenas–Turanzas 2008[21]	Low	Low	Low	Low	Low	Low	Low
Hovland 2010[22]	Low	Low	Low	High[c]	Low	Low	Low
Histology-based Correction Studies							
Schneider 2000[23]	Low	Low	High[d]	High[e]	Low	Low	Low
Kulasingam 2002[24]	Low	Low	Unclear[f]	High[e]	Low	Low	Low
Bigras 2005[26]	Low	Low	Unclear[f,g]	High[e,h]	Low	Low	Low
Mayrand 2007[59]	Low	Low	Low	High[e]	Low	Low	Low
Li 2009[28]	Unclear[i,j]	Low	High[d,f]	High[e,h]	Unclear[j]	Low	low
Castle 2011[29]	Low	Low	Low	High[e]	Low	Low	Low
Mahmud 2012[30]	Low	Low	High[d]	High[e]	Low	Low	Low
Sangrajrang 2017[31]	Unclear[i,j]	Low	Unclear[f,g]	High[e,h]	Unclear[j]	Low	Low
Kurokawa 2018[33]	High[k]	Low	Unclear[f]	High[e,h]	Low	Low	Low
Colposcopy-based Studies							
Blumenthal 2001[34,60]	Unclear[i]	Low	Low	High[h,l]	Unclear[j]	Low	Low
Coste 2003[35]	High[j,m]	Low	Low	High[h,l]	Unclear[j]	Low	Low
Sankaranarayanan 2004[37]	Unclear[j]	Low	Low	High[h,l,n]	High[a]	Low	Low
Qiao 2008[38]	High[k]	Low	High[d]	High[l,o]	Low	Low	Low
McAdam 2010[39]	Low	Low	High[d,g]	High[l]	Low	Low	Low
Quincy 2012[40]	Low	Low	Unclear[g]	High[h,l]	Low	Low	Low
Colposcopy-based Correction Studies							
Cuzick 2003[41]	Low	Low	High[d]	High[p]	Low	Low	Low
Petry 2003[42]	Low	Low	Unclear[f]	High[p]	Low	Low	Low
Gravitt 2010[43]	High[k]	Low	High[d]	High[p]	High[a]	Low	Low
Moy 2010[44]	Low	Low	Unclear[f,g]	High[p]	High[a]	Low	Low
Monsonego 2011[45]	Low	Low	High[f,q]	High[p]	Low	Low	Low
Ferreccio 2013[46]	Low	Low	Low	High[p]	Low	Low	Low
Agorastos 2015[47]	Low	Low	High[d]	High[p]	Low	Low	Low
Iftner 2015[48]	Low	Low	High[d]	High[p]	Low	Low	Low

Wu 2017[49]	High[k]	Low	High[d]	High[p]	Low	Low	Low

Color Key

Low risk of bias or concerns regarding applicability	Unclear risk of bias or concerns regarding applicability	High risk of bias or concerns regarding applicability

Figure S2. Risk of Bias and Concerns Regarding the Applicability of the Included Studies

a. This study enrolled only women who had never participated in cervical cancer screening.

b. The age range for the enrolled women was narrow; between 35 and 45 years of age only.

c. 8.7% of the women with missing or unsatisfactory results were excluded.

d. The colposcopists were aware of the participants' screening test results.

e. Although statistically corrected, differential verification was performed on the basis of screening tests and/or colposcopy results.

f. No description on whether the colposcopists were aware of screening test results.

g. No description on whether the pathologists were aware of screening test results.

h. No explicit description about data exclusion due to missing or unsatisfactory results.

i. The proportion of women who were potentially eligible but not included in the study was not described.

j. No description of exclusion criteria.

k. A high proportion of eligible women were not included in the study.

l. No corrections for verification bias in women without biopsy (all received colposcopy but pathological verification was performed in only women with abnormal colposcopy).

m. 25% of eligible subjects were excluded.

n. 10.9% of women with missing or unsatisfactory results were excluded.

o. 5.6% of women were excluded.

p. Verification bias was corrected but based on the colposcopy-directed results only (i.e., pathological confirmation was performed in only selected women).

q. The pathologists were aware of screening test results.

Figure S3

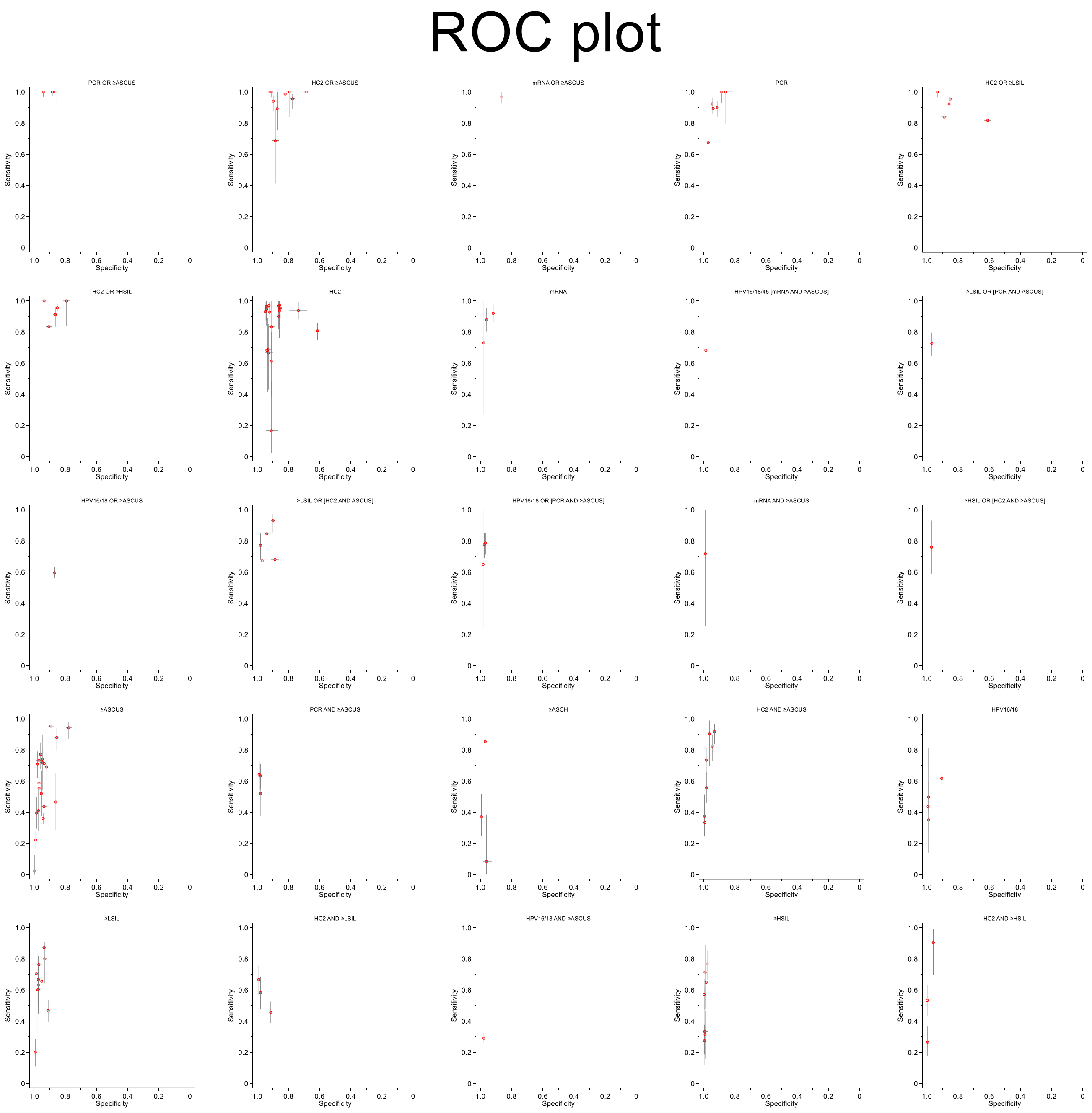

r.

Figure S3. Cross-hair Plots of Standalone Tests and Combination Algorithms.

Cross-hair ROC plots (left panel) show reported point estimates (shown as red circles) and confidence intervals (shown as black extended lines).

ASCH, atypical squamous cells cannot exclude high-grade lesion; ASCUS, atypical squamous cells of undetermined significance; HC2, Hybrid Capture 2; HPV16/18(/45), genotyping for HPV types 16 or 18 (or 45); hrHPV, high-risk genotypes of human papillomavirus; HSIL, high-grade squamous intraepithelial lesion; LBC, liquid-based cytology; LSIL, low-grade squamous intraepithelial lesion; mRNA, messenger ribonucleic acid; PCR, polymerase chain reaction; ROC, receiver operating characteristic

s. Figure S4

Meta-analysis of screening accuracy

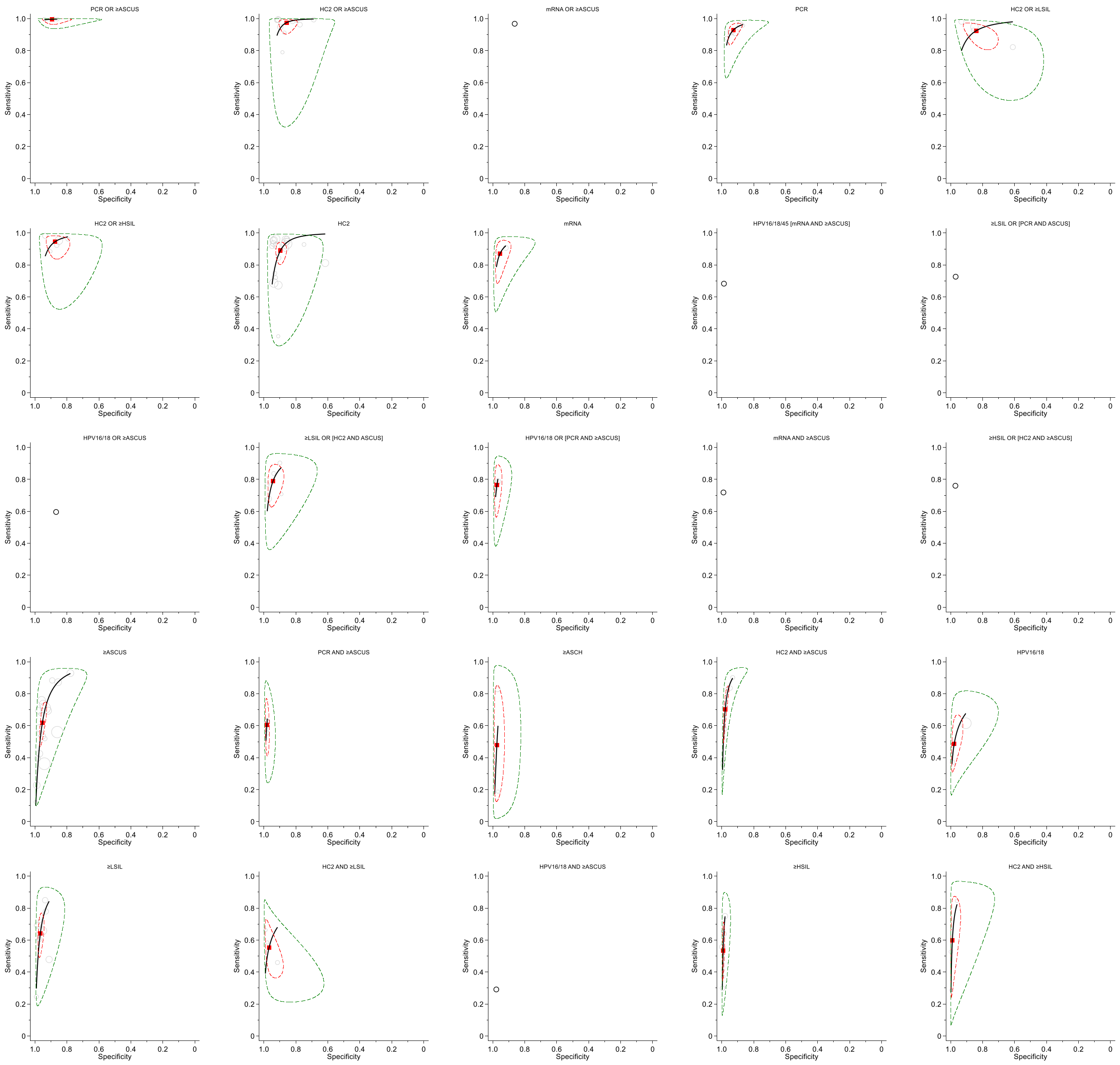

t.

Figure S4. Standard Meta-analysis of Screening Accuracy of Standalone Tests and Combination Algorithms.

The ROC plots of individual study point estimates (the size of each circle is proportional to the sample size for each study) and hierarchical summary ROC curves are shown. The dashed red and green elliptical boundaries represent, respectively, the 95% credible and predicted regions for the summary estimates (closed red square). A meta-analysis was performed when data were available in three or more studies. In this case, the point estimates represent the posterior, “adjusted” values.

ASCH, atypical squamous cells cannot exclude high-grade lesion; ASCUS, atypical squamous cells of undetermined significance; HC2, Hybrid Capture 2; HPV16/18(/45), genotyping for HPV types 16 or 18 (or 45); HSIL, high-grade

squamous intraepithelial lesion; LBC, liquid-based cytology; LSIL, low-grade squamous intraepithelial lesion; mRNA, messenger ribonucleic acid; PCR, polymerase chain reaction; ROC, receiver operating characteristic.

Figure S5

Screening test	Study, n	Patients, n	Average sensitivity (95%CrI)	Average specificity (95%CrI)
PCR OR ≥ASCUS	3	22,642	0.996 (0.980 to 0.999)	0.894 (0.794 to 0.944)
HC2 OR ≥ASCUS	10	53,303	0.975 (0.931 to 0.993)	0.858 (0.804 to 0.898)
mRNA OR ≥ASCUS	1	4,429	0.968 (0.928 to 1.000)	0.863 (0.853 to 0.874)
HC2 OR ≥HSIL	5	25,084	0.946 (0.871 to 0.980)	0.873 (0.799 to 0.920)
PCR	6	32,720	0.929 (0.861 to 0.966)	0.927 (0.883 to 0.954)
HC2 OR ≥LSIL	5	25,402	0.924 (0.836 to 0.967)	0.840 (0.729 to 0.907)
HC2	20	109,245	0.891 (0.824 to 0.935)	0.896 (0.866 to 0.920)
mRNA	3	18,926	0.871 (0.721 to 0.941)	0.955 (0.897 to 0.977)
≥LSIL OR [HC2 AND ASCUS]	5	25,639	0.790 (0.665 to 0.879)	0.942 (0.888 to 0.969)
HPV16/18 OR [PCR AND ≥ASCUS]	3	23,695	0.766 (0.598 to 0.869)	0.974 (0.948 to 0.985)
≥HSIL OR [HC2 AND ≥ASCUS]	1	1,755	0.760 (0.590 to 0.930)	0.970 (0.970 to 0.980)
≥LSIL OR [PCR AND ASCUS]	1	11,064	0.727 (0.648 to 0.796)	0.969 (0.965 to 0.972)
mRNA AND ≥ASCUS	1	5,046	0.718 (0.256 to 1.000)	0.989 (0.985 to 0.992)
HC2 AND ≥ASCUS	7	46,687	0.701 (0.544 to 0.829)	0.978 (0.960 to 0.988)
HPV16/18/45 OR [mRNA AND ≥ASCUS]	1	5,046	0.683 (0.245 to 1.000)	0.986 (0.982 to 0.990)
≥LSIL	11	47,302	0.641 (0.523 to 0.747)	0.966 (0.948 to 0.978)
≥ASCUS	20	153,178	0.619 (0.499 to 0.727)	0.953 (0.932 to 0.968)
PCR AND ≥ASCUS	4	27,688	0.605 (0.445 to 0.740)	0.981 (0.968 to 0.988)
HC2 AND ≥HSIL	3	14,675	0.597 (0.326 to 0.850)	0.989 (0.939 to 0.997)
HPV16/18 OR ≥ASCUS	1	40,901	0.596 (0.561 to 0.630)	0.868 (0.864 to 0.871)
HC2 AND ≥LSIL	3	14,993	0.554 (0.396 to 0.696)	0.967 (0.889 to 0.987)
≥HSIL	7	18,838	0.534 (0.382 to 0.682)	0.991 (0.984 to 0.995)
HPV16/18	4	62,941	0.487 (0.342 to 0.634)	0.977 (0.931 to 0.991)
≥ASCH	3	10,491	0.478 (0.162 to 0.790)	0.975 (0.937 to 0.989)
HPV16/18 AND ≥ASCUS	1	40,901	0.291 (0.260 to 0.324)	0.978 (0.977 to 0.980)

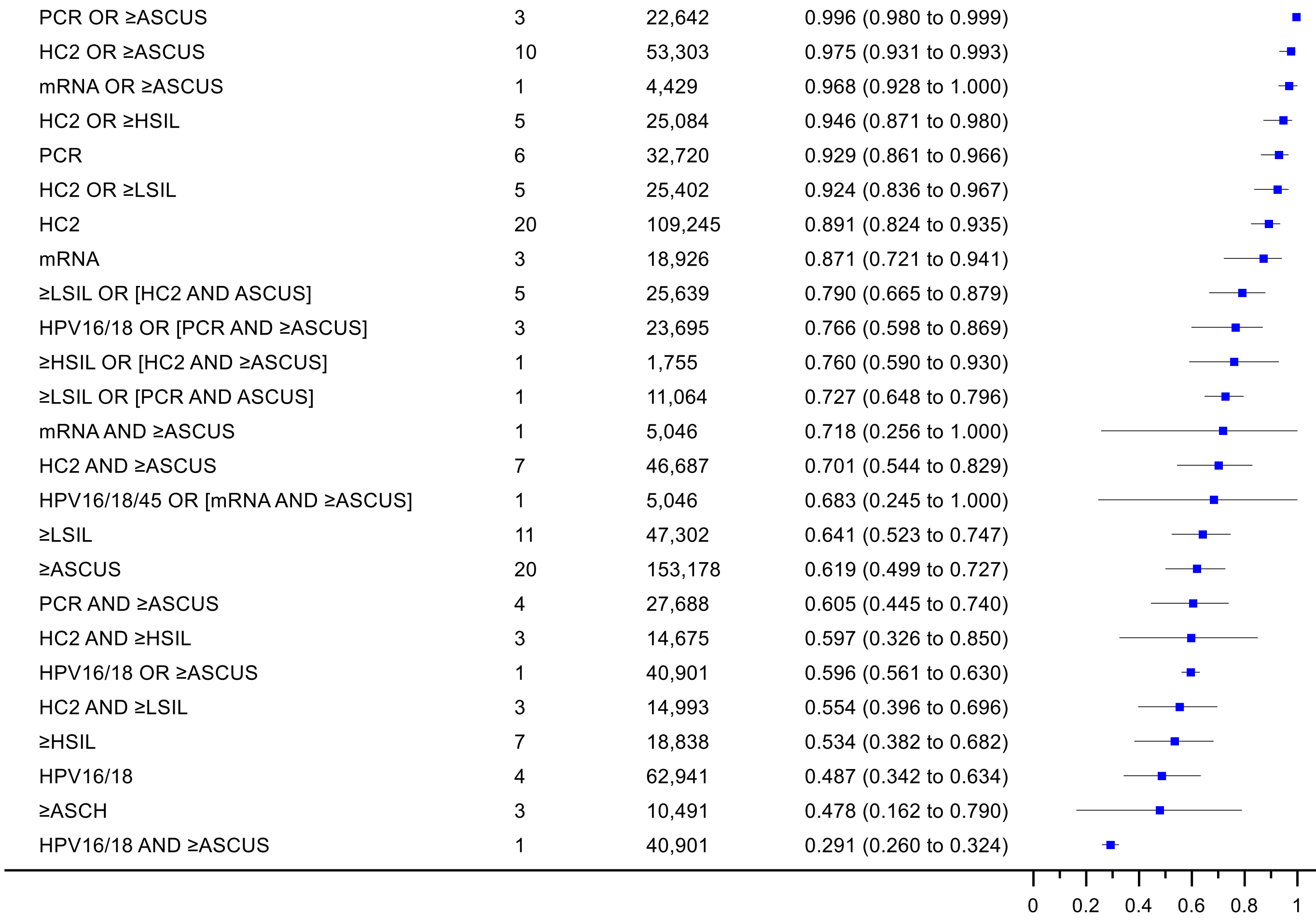

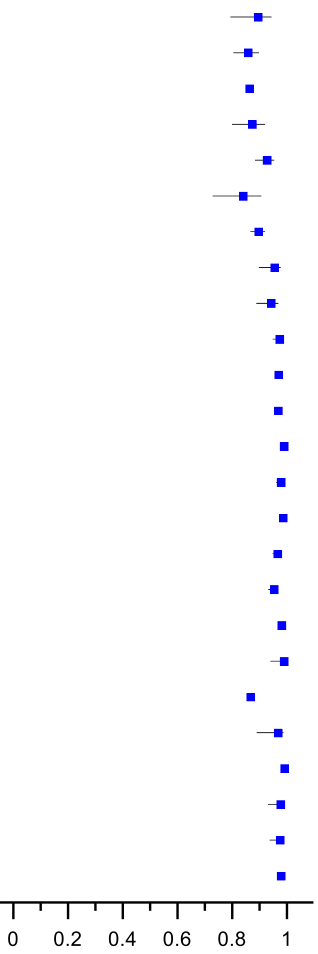

Figure S5. Summary of Standard Meta-analysis of Screening Accuracy.

Standalone tests or combinations algorithm were ranked by the point estimates for the average sensitivity. Meta analysis was performed when there were ≥3 studies. ASCH, atypical squamous cells cannot exclude high grade lesion; ASCUS, atypical squamous cells of undetermined significance; CrI, credible interval; HC2, Hybrid Capture 2; HPV16/18(/45), genotyping for HPV types 16 or 18 (or 45); HSIL, high-grade squamous intraepithelial lesion; LBC, liquid-based cytology; LSIL, low-grade squamous intraepithelial lesion; mRNA, messenger ribonucleic acid; PCR, polymerase chain reaction.

Figure S6 A

ROC plot; part 1 of 5

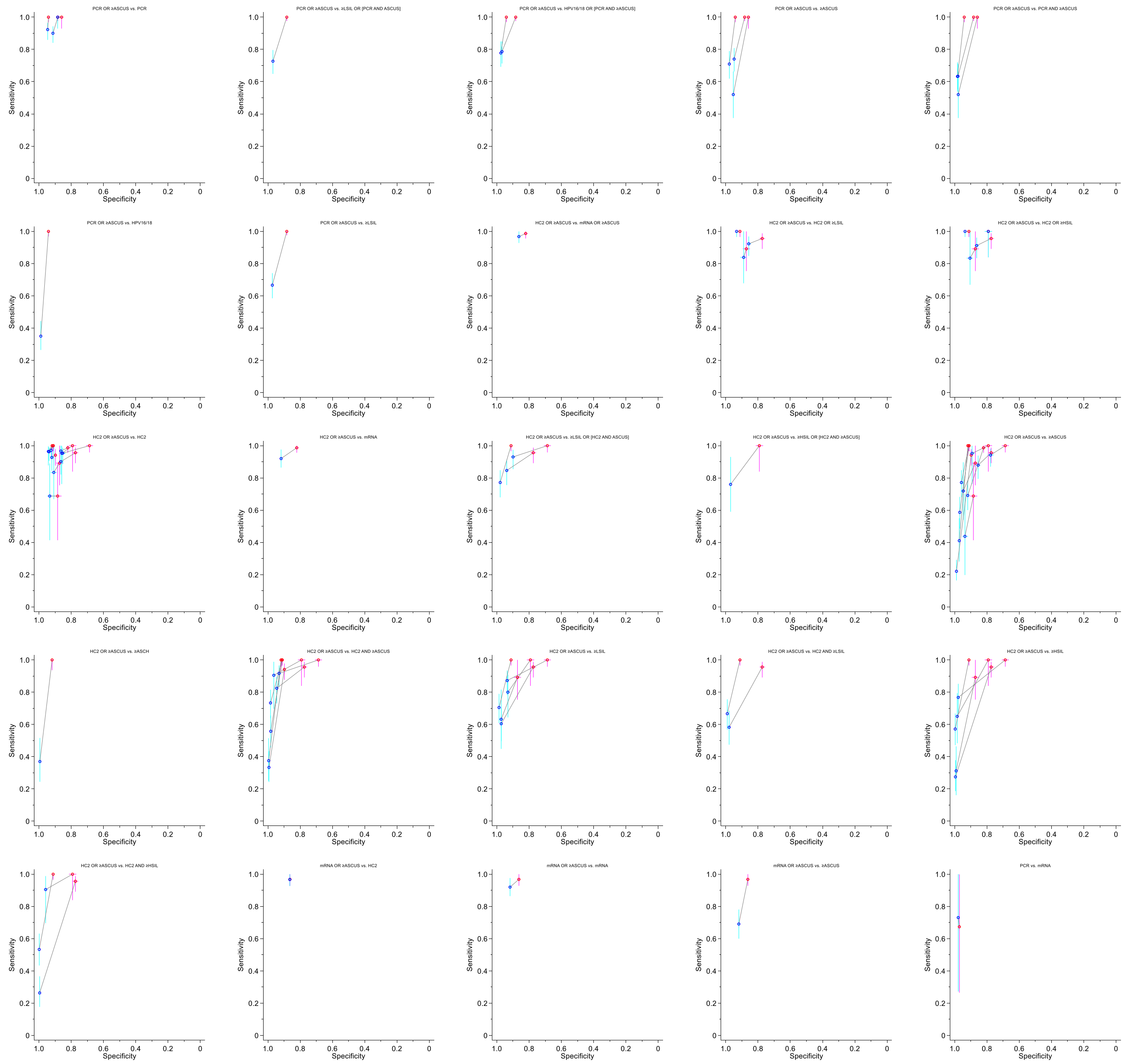

Figure S6 B

ROC plot; part 2 of 5

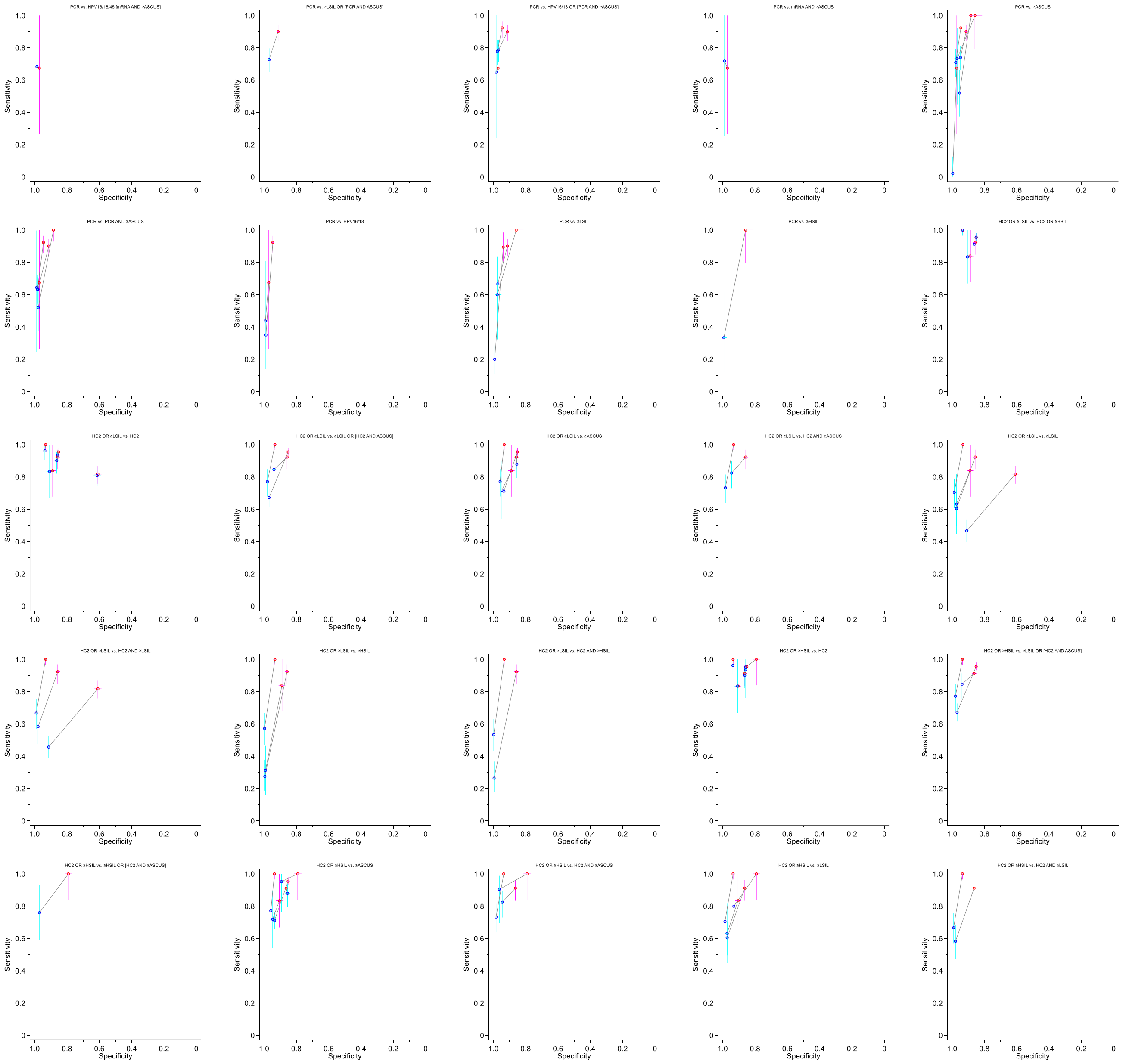

Figure S6 C

ROC plot; part 3 of 5

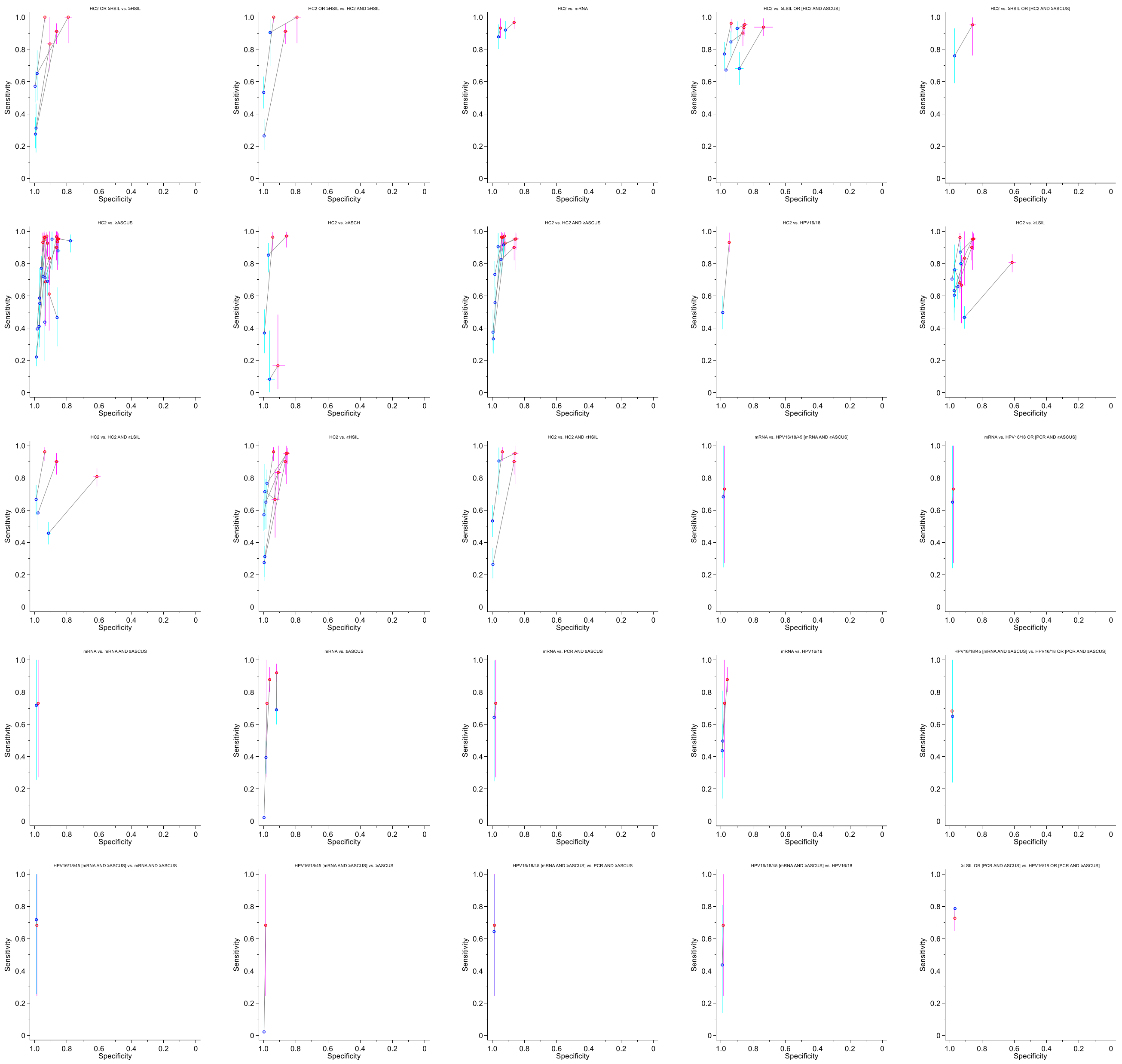

Figure S6 D

ROC plot; part 4 of 5

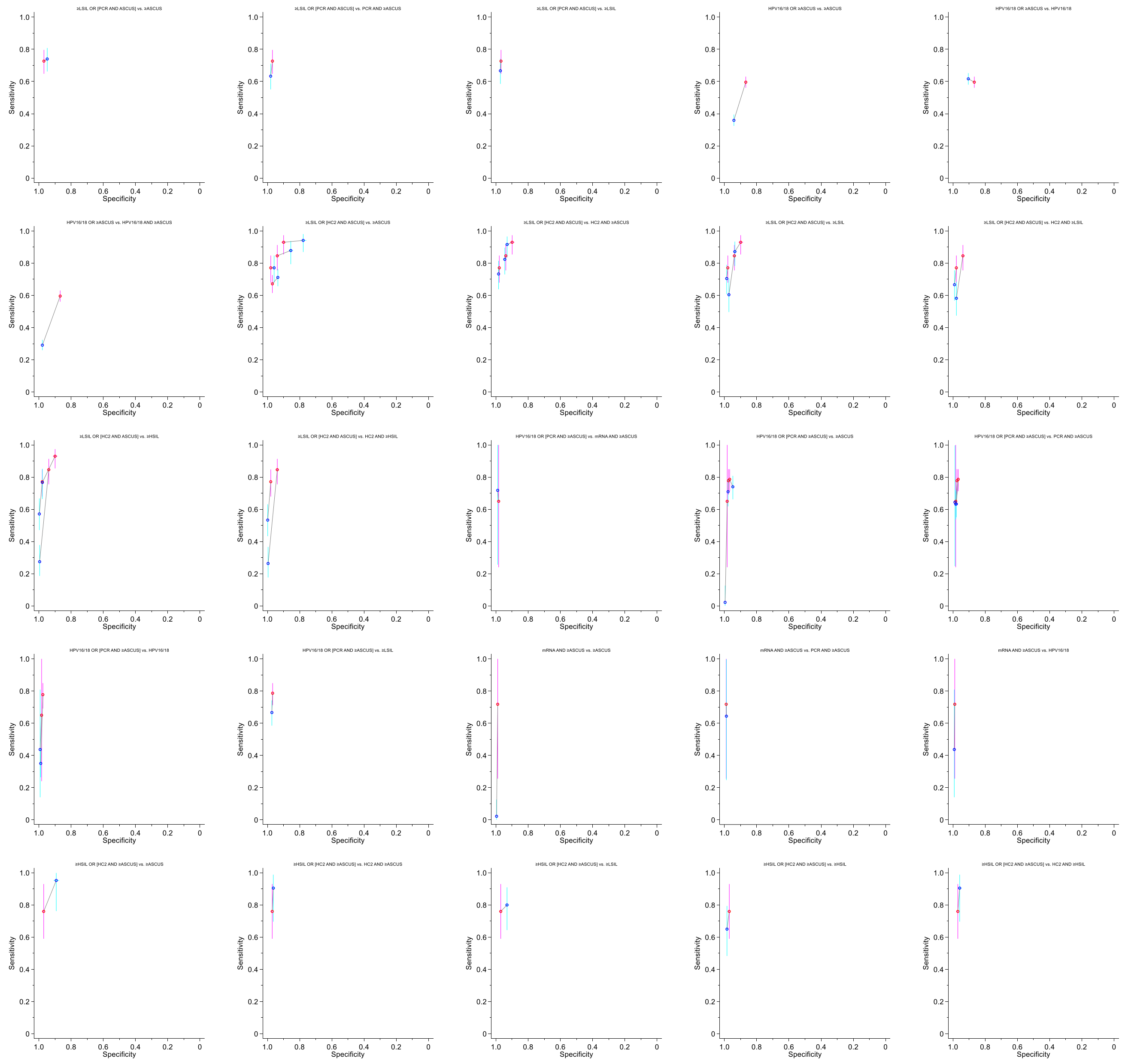

Figure S6 E

ROC plot; part 5 of 5

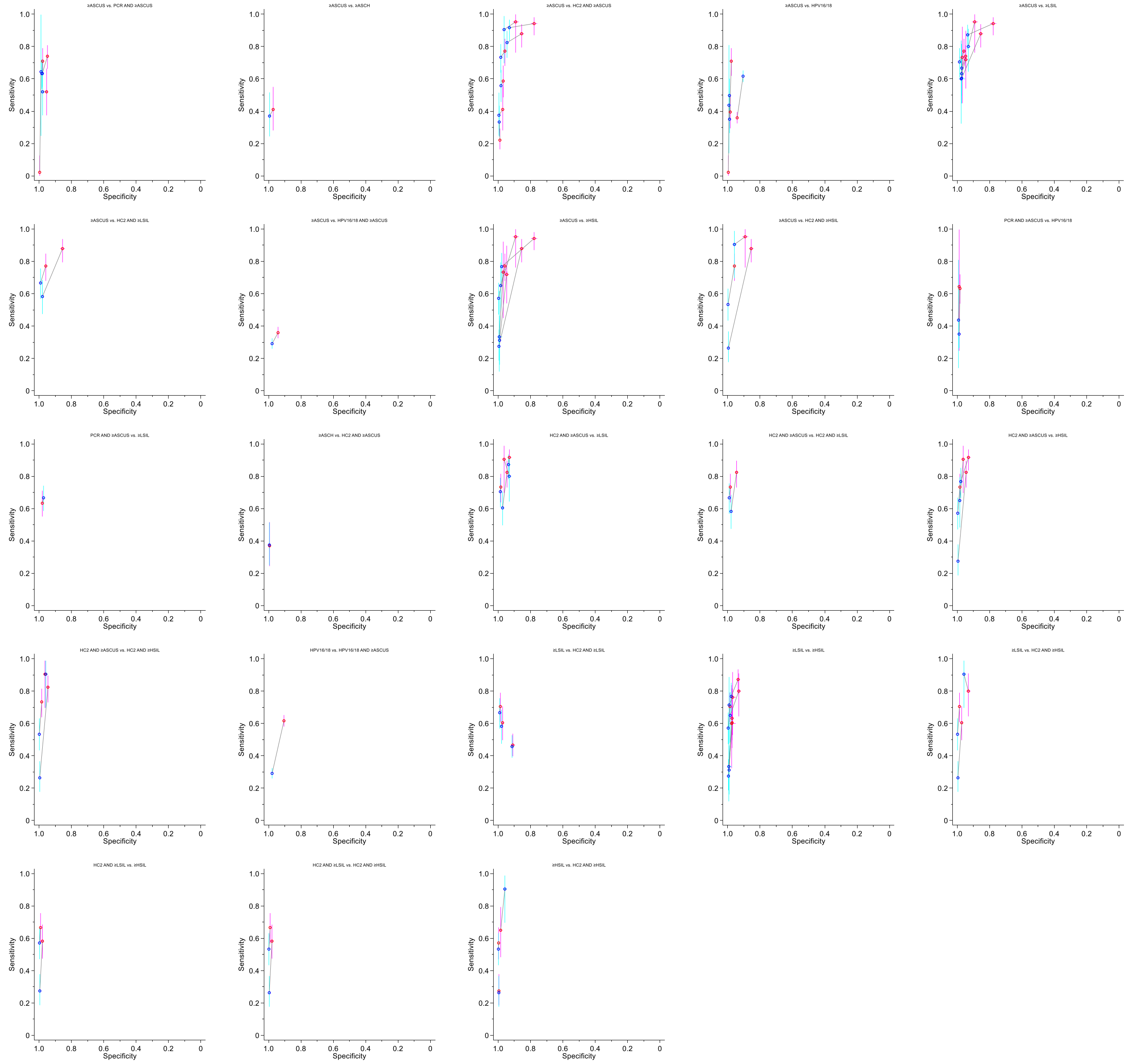

Figure S6. Comparison of Screening Accuracy Among Alternative Tests and/or Combination Algorithms

Cross-hair ROC plots show reported point estimates (shown as circles), and confidence intervals (shown as extended lines) for the first test or algorithm (red) and the second test or algorithm (blue). Figure S6 consists of 5 parts (A, B, C, D, and E).

ASCH, atypical squamous cells cannot exclude high grade lesion; ASCUS, atypical squamous cells of undetermined significance; HC2, Hybrid Capture 2; HPV16/18(/45), genotyping for HPV types 16 or 18 (or 45); HSIL, high-grade squamous intraepithelial lesion; LBC, liquid-based cytology; LSIL, low-grade squamous intraepithelial lesion; mRNA, messenger ribonucleic acid; PCR, polymerase chain reaction.

Figure S7

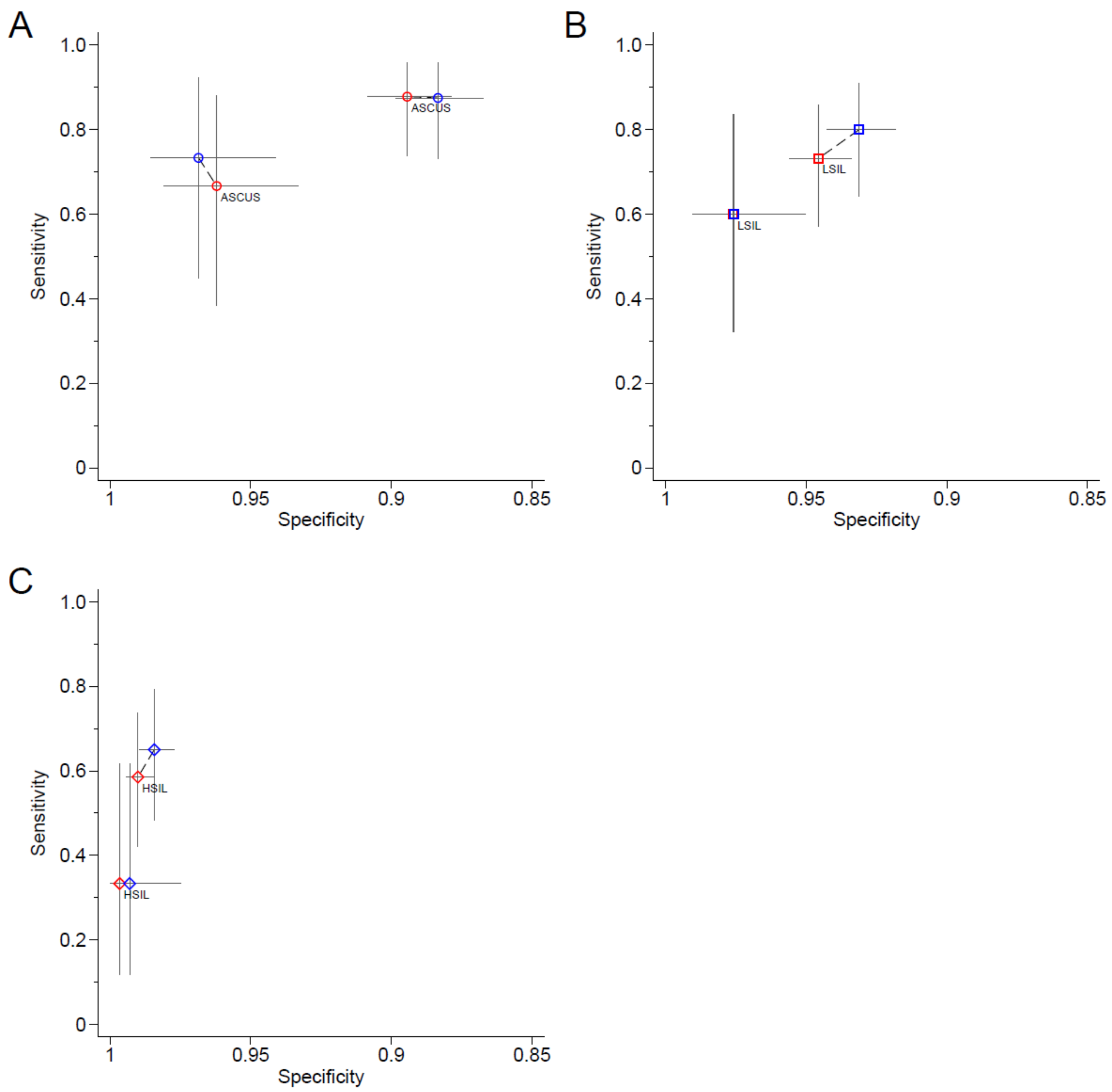

Figure S7. Comparison of Screening Accuracy Among Alternative Preparation Methods for Cytologic Testing

Cross-hair ROC plots and confidence intervals (extended lines) are shown for conventional cytology (red) and liquid-based cytology (blue). Comparisons are for (A) ≥ASCUS (open circles), (B) ≥LSIL (open squares), and (C) ≥HSIL (open diamonds) thresholds.

≥ASCUS, atypical squamous cells of undetermined significance or higher grades; CIN2+, cervical intraepithelial neoplasia grade 2 or worse; ≥HSIL, high-grade squamous intraepithelial lesion or higher grades; ≥LSIL, low-grade squamous intraepithelial lesion or higher grades; ROC, receiver operating characteristic.

Figure S8

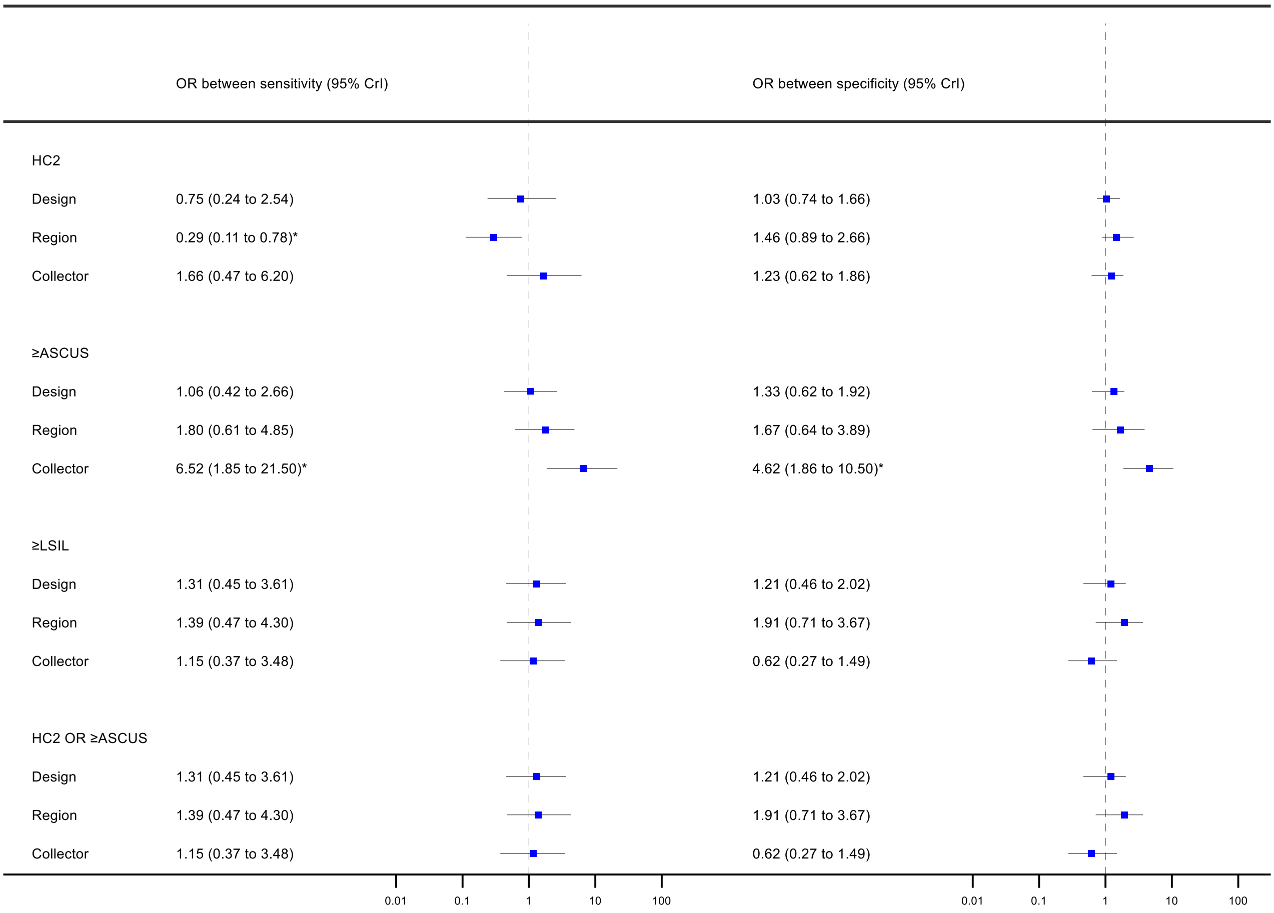

Figure S8. Modifiers of Sensitivity and Specificity

The results are based on univariable bivariate random-effects meta-regression allowing a prespecified clinical variable to affect both sensitivity and specificity. Squares represent point estimates; extended lines represent the 95% credible interval of each estimate. Single asterisks represent statistically significant results.

ASCUS, atypical squamous cells of undetermined significance; CrI, credible interval; HC2, hybrid capture 2; LSIL, low-grade squamous intraepithelial lesion; OR, odds ratio.

ONLINE-ONLY REFERENCES

1. Hamashima C, Aoki D, Miyagi E, et al. The Japanese guideline for cervical cancer screening. *Jpn J Clin Oncol.* 2010;40(6):485-502.
2. The Japanese Research Group for Systematic Review and Guideline Development for Cancer Screening. An evidence report for the Japanese guideline for cervical cancer screening 2019. 2020:1-258. http://canscreen.ncc.go.jp/guideline/shikyukeireport2019.pdf. Published 2020/03/31. Accessed May 5, 2021.
3. Naaktgeboren CA, de Groot JAH, Rutjes AWS, Bossuyt PMM, Reitsma JB, Moons KGM. Anticipating missing reference standard data when planning diagnostic accuracy studies. *BMJ.* 2016;352.
4. Begg CB, Greenes RA. Assessment of diagnostic tests when disease verification is subject to selection bias. *Biometrics.* 1983;39(1):207-215.
5. Owen RK, Cooper NJ, Quinn TJ, Lees R, Sutton AJ. Network meta-analysis of diagnostic test accuracy studies identifies and ranks the optimal diagnostic tests and thresholds for health care policy and decision-making. *J Clin Epidemiol.* 2018;99:64-74.
6. Menten J, Boelaert M, Lesaffre E. Bayesian meta-analysis of diagnostic tests allowing for imperfect reference standards. *Stat Med.* 2013;32(30):5398-5413.
7. Harbord RM, Deeks JJ, Egger M, Whiting P, Sterne JA. A unification of models for meta-analysis of diagnostic accuracy studies. *Biostatistics.* 2007;8(2):239-251.
8. Rutter CM, Gatsonis CA. A hierarchical regression approach to meta-analysis of diagnostic test accuracy evaluations. *Stat Med.* 2001;20(19):2865-2884.
9. Arends LR, Hamza TH, van Houwelingen JC, Heijenbrok-Kal MH, Hunink MG, Stijnen T. Bivariate random effects meta-analysis of ROC curves. *Med Decis Making.* 2008;28(5):621-638.
10. Human Development Indicators and Indices: 2018 Statistical Update Team. United Nations Development Programme; 2018. http://hdr.undp.org/sites/default/files/2018_human_development_statistical_update.pdf.
11. Chu H, Nie L, Cole SR, Poole C. Meta-analysis of diagnostic accuracy studies accounting for disease prevalence: alternative parameterizations and model selection. *Stat Med.* 2009;28(18):2384-2399.
12. Merckx J, Wali R, Schiller I, et al. Diagnostic Accuracy of Novel and Traditional Rapid Tests for Influenza Infection Compared With Reverse Transcriptase Polymerase Chain Reaction: A Systematic Review and Meta-analysis. *Ann Intern Med.* 2017;167(6):394-409.
13. Welton NJ, Sutton AJ, Cooper N, Abrams KR, Ades AE. *Evidence Synthesis for Decision Making in Healthcare.* Wiley; 2012.
14. Schunemann HJ, Oxman AD, Brozek J, et al. Grading quality of evidence and strength of recommendations for diagnostic tests and strategies. *Bmj.* 2008;336(7653):1106-1110.
15. Koliopoulos G, Nyaga VN, Santesso N, et al. Cytology versus HPV testing for cervical cancer screening in the general population. *Cochrane Database Syst Rev.* 2017;8:Cd008587.
16. McInnes MDF, Moher D, Thombs BD, et al. Preferred Reporting Items for a Systematic Review and Meta-analysis of Diagnostic Test Accuracy Studies: The PRISMA-DTA Statement. *Jama.* 2018;319(4):388-396.
17. Thompson J. *Bayesian Analysis with Stata.* Stata Press; 2014.
18. Belinson J, Qiao YL, Pretorius R, et al. Shanxi Province Cervical Cancer Screening Study: a cross-sectional comparative trial of multiple techniques to detect cervical neoplasia. *Gynecol Oncol.* 2001;83(2):439-444.

19. Pan Q, Belinson JL, Li L, et al. A thin-layer, liquid-based pap test for mass screening in an area of China with a high incidence of cervical carcinoma. A cross-sectional, comparative study. *Acta Cytol.* 2003;47(1):45-50.
20. Zhao FH, Zhang WH, Pan QJ, et al. [A study of cervical cancer screening algorithms]. *Zhonghua Zhong Liu Za Zhi.* 2010;32(6):420-424.
21. Cardenas-Turanzas M, Nogueras-Gonzalez GM, Scheurer ME, et al. The performance of human papillomavirus high-risk DNA testing in the screening and diagnostic settings. *Cancer Epidemiol Biomarkers Prev.* 2008;17(10):2865-2871.
22. Hovland S, Arbyn M, Lie AK, et al. A comprehensive evaluation of the accuracy of cervical pre-cancer detection methods in a high-risk area in East Congo. *Br J Cancer.* 2010;102(6):957-965.
23. Schneider A, Hoyer H, Lotz B, et al. Screening for high-grade cervical intra-epithelial neoplasia and cancer by testing for high-risk HPV, routine cytology or colposcopy. *Int J Cancer.* 2000;89(6):529-534.
24. Kulasingam SL, Hughes JP, Kiviat NB, et al. Evaluation of human papillomavirus testing in primary screening for cervical abnormalities: comparison of sensitivity, specificity, and frequency of referral. *JAMA.* 2002;288(14):1749-1757.
25. Balasubramanian A, Kulasingam SL, Baer A, et al. Accuracy and cost-effectiveness of cervical cancer screening by high-risk human papillomavirus DNA testing of self-collected vaginal samples. *J Low Genit Tract Dis.* 2010;14(3):185-195.
26. Bigras G, de Marval F. The probability for a Pap test to be abnormal is directly proportional to HPV viral load: results from a Swiss study comparing HPV testing and liquid-based cytology to detect cervical cancer precursors in 13,842 women. *Br J Cancer.* 2005;93(5):575-581.
27. Mayrand MH, Duarte-Franco E, Coutlee F, et al. Randomized controlled trial of human papillomavirus testing versus Pap cytology in the primary screening for cervical cancer precursors: design, methods and preliminary accrual results of the Canadian cervical cancer screening trial (CCCaST). *Int J Cancer.* 2006;119(3):615-623.
28. Li N, Shi JF, Franceschi S, et al. Different cervical cancer screening approaches in a Chinese multicentre study. *Br J Cancer.* 2009;100(3):532-537.
29. Castle PE, Stoler MH, Wright TC, Jr., Sharma A, Wright TL, Behrens CM. Performance of carcinogenic human papillomavirus (HPV) testing and HPV16 or HPV18 genotyping for cervical cancer screening of women aged 25 years and older: a subanalysis of the ATHENA study. *Lancet Oncol.* 2011;12(9):880-890.
30. Mahmud SM, Sangwa-Lugoma G, Nasr SH, et al. Comparison of human papillomavirus testing and cytology for cervical cancer screening in a primary health care setting in the Democratic Republic of the Congo. *Gynecol Oncol.* 2012;124(2):286-291.
31. Sangrajrang S, Laowahutanont P, Wongsena M, et al. Comparative accuracy of Pap smear and HPV screening in Ubon Ratchathani in Thailand. *Papillomavirus Res.* 2017;3:30-35.
32. Sangrajrang S, Laowahutanont P, Wongsena M, et al. Human papillomavirus (HPV) DNA and mRNA primary cervical cancer screening: Evaluation and triaging options for HPV-positive women. *J Med Screen.* 2019;26(4):212-218.
33. Kurokawa T, Onuma T, Shinagawa A, Chino Y, Kobayashi M, Yoshida Y. The ideal strategy for cervical cancer screening in Japan: Result from the Fukui Cervical Cancer Screening Study. *Cytopathology.* 2018;29(4):361-367.
34. Blumenthal PD, Gaffikin L, Chirenje ZM, McGrath J, Womack S, Shah K. Adjunctive testing for cervical cancer in low resource settings with visual inspection, HPV, and the Pap smear. *Int J Gynaecol Obstet.* 2001;72(1):47-53.
35. Coste J, Cochand-Priollet B, de Cremoux P, et al. Cross sectional study of conventional cervical smear, monolayer cytology, and human papillomavirus DNA testing for cervical cancer screening. *Bmj.* 2003;326(7392):733.
36. de Cremoux P, Coste J, Sastre-Garau X, et al. Efficiency of the hybrid capture 2 HPV DNA test in cervical cancer screening. A study by the French Society of Clinical Cytology. *Am J Clin Pathol.* 2003;120(4):492-499.

37. Sankaranarayanan R, Chatterji R, Shastri SS, et al. Accuracy of human papillomavirus testing in primary screening of cervical neoplasia: results from a multicenter study in India. *Int J Cancer.* 2004;112(2):341-347.
38. Qiao YL, Sellors JW, Eder PS, et al. A new HPV-DNA test for cervical-cancer screening in developing regions: a cross-sectional study of clinical accuracy in rural China. *Lancet Oncol.* 2008;9(10):929-936.
39. McAdam M, Sakita J, Tarivonda L, Pang J, Frazer IH. Evaluation of a cervical cancer screening program based on HPV testing and LLETZ excision in a low resource setting. *PLoS One.* 2010;5(10):e13266.
40. Quincy BL, Turbow DJ, Dabinett LN, Dillingham R, Monroe S. Diagnostic accuracy of self-collected human papillomavirus specimens as a primary screen for cervical cancer. *J Obstet Gynaecol.* 2012;32(8):795-799.
41. Cuzick J, Szarewski A, Cubie H, et al. Management of women who test positive for high-risk types of human papillomavirus: the HART study. *Lancet.* 2003;362(9399):1871-1876.
42. Petry KU, Menton S, Menton M, et al. Inclusion of HPV testing in routine cervical cancer screening for women above 29 years in Germany: results for 8466 patients. *Br J Cancer.* 2003;88(10):1570-1577.
43. Gravitt PE, Paul P, Katki HA, et al. Effectiveness of VIA, Pap, and HPV DNA testing in a cervical cancer screening program in a peri-urban community in Andhra Pradesh, India. *PLoS One.* 2010;5(10):e13711.
44. Moy LM, Zhao FH, Li LY, et al. Human papillomavirus testing and cervical cytology in primary screening for cervical cancer among women in rural China: comparison of sensitivity, specificity, and frequency of referral. *Int J Cancer.* 2010;127(3):646-656.
45. Monsonego J, Hudgens MG, Zerat L, et al. Evaluation of oncogenic human papillomavirus RNA and DNA tests with liquid-based cytology in primary cervical cancer screening: the FASE study. *Int J Cancer.* 2011;129(3):691-701.
46. Ferreccio C, Barriga MI, Lagos M, et al. Screening trial of human papillomavirus for early detection of cervical cancer in Santiago, Chile. *Int J Cancer.* 2013;132(4):916-923.
47. Agorastos T, Chatzistamatiou K, Katsamagkas T, et al. Primary screening for cervical cancer based on high-risk human papillomavirus (HPV) detection and HPV 16 and HPV 18 genotyping, in comparison to cytology. *PLoS One.* 2015;10(3):e0119755.
48. Iftner T, Becker S, Neis KJ, et al. Head-to-Head Comparison of the RNA-Based Aptima Human Papillomavirus (HPV) Assay and the DNA-Based Hybrid Capture 2 HPV Test in a Routine Screening Population of Women Aged 30 to 60 Years in Germany. *J Clin Microbiol.* 2015;53(8):2509-2516.
49. Wu Q, Zhao X, Fu Y, et al. A cross-sectional study on HPV testing with type 16/18 genotyping for cervical cancer screening in 11,064 Chinese women. *Cancer Med.* 2017;6(5):1091-1101.
50. Ratnam S, Franco EL, Ferenczy A. Human papillomavirus testing for primary screening of cervical cancer precursors. *Cancer Epidemiol Biomarkers Prev.* 2000;9(9):945-951.
51. Almonte M, Ferreccio C, Winkler JL, et al. Cervical screening by visual inspection, HPV testing, liquid-based and conventional cytology in Amazonian Peru. *Int J Cancer.* 2007;121(4):796-802.
52. Zhou XH, McClish DK, Obuchowski NA. *Statistical Methods in Diagnostic Medicine.* Wiley; 2002.
53. Choi BC. Sensitivity and specificity of a single diagnostic test in the presence of work-up bias. *J Clin Epidemiol.* 1992;45(6):581-586.
54. Menten J, Boelaert M, Lesaffre E. Bayesian latent class models with conditionally dependent diagnostic tests: a case study. *Stat Med.* 2008;27(22):4469-4488.
55. Zhou XH. Correcting for verification bias in studies of a diagnostic test's accuracy. *Stat Methods Med Res.* 1998;7(4):337-353.

56. Nofuentes JAR, del Castillo JdDL. Comparing two binary diagnostic tests in the presence of verification bias. *Computational Statistics & Data Analysis.* 2006;50(6):1551-1564.

57. Katki HA, Li Y, Edelstein DW, Castle PE. Estimating the agreement and diagnostic accuracy of two diagnostic tests when one test is conducted on only a subsample of specimens. *Stat Med.* 2012;31(5):436-448.

58. Sasieni P. Estimating prevalence when the true disease status is incompletely ascertained. *Stat Med.* 2001;20(6):935-949.

59. Mayrand MH, Duarte-Franco E, Rodrigues I, et al. Human papillomavirus DNA versus Papanicolaou screening tests for cervical cancer. *N Engl J Med.* 2007;357(16):1579-1588.

60. Womack SD, Chirenje ZM, Blumenthal PD, et al. Evaluation of a human papillomavirus assay in cervical screening in Zimbabwe. *BJOG.* 2000;107(1):33-38.

LIST OF EXCLUDED PUBLICATIONS (n = 222)

No correction for verification bias (n = 73)

1. Agorastos T, Dinas K, Lloveras B, de Sanjose S, Kornegay JR, Bonti H, et al. Human papillomavirus testing for primary screening in women at low risk of developing cervical cancer. The Greek experience. Gynecol Oncol 2005;96:714–20.
2. Asthana S, Labani S, Sodhani P et al. Co-testing of cervical screening tests in detection of high grade cervical intraepithelial neoplasia. Journal of Clinical and Diagnostic Research 2017; 11:10 (XC10-XC14).
3. Austin RM, Onisko A, Zhao C et al. Enhanced Detection of Cervical Cancer and Precancer Through Use of Imaged Liquid-Based Cytology in Routine Cytology and HPV Cotesting. American Journal of Clinical Pathology. 2018;150(5):385-392.
4. Belinson JL, Du H, Yang B, et al. Improved sensitivity of vaginal self-collection and high-risk human papillomavirus testing. Int J Cancer. 2012;130(8):1855-60.
5. Belinson JL, Qiao YL, Pretorius RG, Zhang WH, Rong SD, Huang MN, et al. Shanxi Province cervical cancer screening study II: self-sampling for high-risk human papillomavirus compared to direct sampling for human papillomavirus and liquid based cervical cytology. Int J Gynecol Cancer 2003;13:819–26.
6. Belinson SE, Wulan N, Li R, Zhang W, Rong X, Zhu Y, et al. SNIPER: a novel assay for human papillomavirus testing among women in Guizhou, China. International Journal of Gynecological Cancer 2010;20:1006-10.
7. Chatzistamatiou K, Moysiadis T, Moschaki V, et al. Comparison of cytology, HPV DNA testing and HPV 16/18 genotyping alone or combined targeting to the more balanced methodology for cervical cancer screening. Gynecol Oncol. 2016;142(1):120-127.
8. Clavel C, Masure M, Bory JP, Putaud I, Mangeonjean C, Lorenzato M, et al. Human papillomavirus testing in primary screening for the detection of high-grade cervical lesions: a study of 7932 women. Br J Cancer 2001;84:1616–23.
9. Cook DA, Mei W, Smith LW, et al. Comparison of the Roche cobas® 4800 and Digene Hybrid Capture® 2 HPV tests for primary cervical cancer screening in the HPV FOCAL trial. BMC Cancer. 2015;15:968.
10. Cook DA, Smith LW, Law J et al. Aptima HPV Assay versus Hybrid Capture 2 HPV test for primary cervical cancer screening in the HPV FOCAL trial. Journal of Clinical Virology.2017;87:23-29.
11. Cook DA, Smith LW, Law JH et al. Comparative performance of human papillomavirus messenger RNA versus DNA screening tests at baseline and 48 months in the HPV FOCAL trial. Journal of Clinical Virology 2018;108:32-37.
12. Cuzick J, Beverley E, Ho L, Terry G, Sapper H, Mielzynska I, et al. HPV testing in primary screening

of older women. Br J Cancer 1999;81:554–8.

13. Cuzick J, Szarewski A, Terry G, Ho L, Hanby A, Maddox P, et al. Human papillomavirus testing in primary cervical screening. Lancet 1995; 345:1533–6.

14. Denny L, Kuhn L, Pollack A, Wainwright H, Wright Jr TC. Evaluation of alternative methods of cervical cancer screening for resource poor settings. Cancer 2000;89:826–33.

15. Duan LF, Du H, Xiao AM, et al. [Relationship between cervical lesions and the type-specific viral load of high risk HPV reflected by the Ct value of Cobas 4800 HPV system]. Zhonghua fu chan ke za zhi. 2019;54(7):458-463.

16. Fujiwara H, Suzuki M, Morisawa H, Sayama M, Kimura K. The Impact of Triage for Atypical Squamous Cells of Undetermined Significance with Human Papillomavirus Testing in Cervical Cancer Screening in Japan. Asian Pacific journal of cancer prevention : APJCP. 2019;20(1):81-85.

17. Girianelli VR, Thuler LC, Szklo M, et al. Comparison of human papillomavirus DNA tests, liquid-based cytology and conventional cytology for the early detection of cervix uteri cancer. Eur J Cancer Prev 2006;15:504–10.

18. Granados R, Tellez-Safina H, Solis I et al. Cervical cancer screening cotesting with cytology and MRNA HPV E6/E7 yields high rates of CIN2+ lesions in young women. Diagnostic Cytopathology 2017;45(12):1065-1072.

19. Han L, Chang X, Song P, et al. An on-going study of three different cervical cancer screening strategies based on primary healthcare facilities in Beijing China. Journal of infection and public health. 2020;13(4):577-583.

20. Holt HK, Zhang L, Zhao FH, et al. Evaluation of multiple primary and combination screening strategies in postmenopausal women for detection of cervical cancer in China. Int J Cancer. 2017;140(3):544-554.

21. Iacobellis M, Violante C, Notarachille G et al. Clinical validation of REALQUALITY RQ-HPV Screen according to the international guidelines for human papillomavirus DNA test requirements for cervical screening. Virology Journal 2018;15(1):48.

22. Jaworek H, Koudelakova V, Drabek J et al. A Head-to-Head Analytical Comparison of Cobas 4800 HPV, PapilloCheck HPV: Screening, and LMNX Genotyping Kit HPV GP for Detection of Human Papillomavirus DNA in Cervical and Cervicovaginal Swabs. Journal of Molecular Diagnostics 2018;20(6):849-858.

23. Jeronimo J, Bansil P, Lim J, et al. A multicountry evaluation of careHPV testing, visual inspection with acetic acid, and papanicolaou testing for the detection of cervical cancer. Int J Gynecol Cancer 2014;24:576-85.

24. Jiang MY, Feng RM, Wang L, et al. [Performance of combined liquid based cytology and HPV nucleic acid test for detecting cervical precancer among women attending screening]. Zhonghua zhong liu za zhi [Chinese journal of oncology]. 2018;40(10):750-756.

25. Kaleli I, Aksoy L, Demir M, et al. [Prevalence and genotype distribution of human papillomavirus in patients attending to gynecology polyclinics]. Mikrobiyoloji bulteni. 2019;53(2):170-178.
26. Kares S, Veijalainen O, Kholova I, et al. HIGH-RISK HPV testing as the primary screening method in an organized regional screening program for cervical cancer: the value of HPV16 and HPV18 genotyping? APMIS : acta pathologica, microbiologica, et immunologica Scandinavica. 2019;127(11):710-716.
27. Kononova IN, Bashmakova NV, Gaeva OE, Krestyaninova TV. Regional experience with virological cervical screening in the Sverdlovsk Region. Akusherstvo i ginekologiya/Obstetrics and gynecology. 2019;8:84-89.
28. Kotaniemi-Talonen L, Nieminen P, Anttila A, et al. Routine cervical screening with primary HPV testing and cytology triage protocol in a randomised setting. Br J Cancer. 2005;93(8):862-7.
29. Kuhn L, Denny L, Pollack A, Lorincz A, Richart RM, Wright TC. Human papillomavirus DNA testing for cervical cancer screening in low-resource settings. J Natl Cancer Inst 2000;92:818–25.
30. Labani S, Asthana S, Sodhani P, Gupta S, Bhambhani S, Pooja B, et al. Care HPV cervical cancer screening demonstration in a rural population of North India. European Journal of Obstetrics & Gynecology and Reproductive Biology 2014;176:75-9.
31. Lazcano-Ponce E, Lorincz AT, Cruz-Valdez A, et al. Self-collection of vaginal specimens for human papillomavirus testing in cervical cancer prevention (MARCH): a community-based randomized controlled trial. Lancet 2011; 378: 1868–73.
32. Levi JE, Martins TR, Longatto-Filho A, et al. High-Risk HPV Testing in Primary Screening for Cervical Cancer in the Public Health System, Sao Paulo, Brazil. Cancer prevention research (Philadelphia, Pa). 2019;12(8):539-546.
33. Liu ZH, Lin W, Wang YY, et al. [Risk stratification of type-specific human papillomavirus for cervical precancers: evidence from a cross-sectional study in Shenzhen]. Zhonghua zhong liu za zhi [Chinese journal of oncology]. 2018;40(10):757-763.
34. Longatto-Filho A, Naud P, Derchain SF, et al. Performance characteristics of Pap test, VIA, VILI, HR-HPV testing, cervicography, and colposcopy in diagnosis of signifi cant cervical pathology. Virchows Arch 2012; 460: 577–85.
35. Luo H, Du H, Belinson JL, Wu R. Evaluation of alternately combining HPV viral load and 16/18 genotyping in secondary screening algorithms. PloS One. 2019;14(7):e0220200.
36. Nakamura M, Nakade K, Orisaka S et al. Comparison Study of BD Onclarity HPV With digene HC2 High-Risk HPV DNA Test and Roche Cobas 4800 HPV for Detecting High-Risk Human Papillomavirus in Japan. American Journal of Clinical Pathology. 2019;151(3):263-269.
37. Nieves L, Enerson CL, Belinson S, et al. Primary cervical cancer screening and triage using an mRNA human papillomavirus assay and visual inspection. Int J Gynecol Cancer 2013; 23: 513–18.
38. Oh YL, Shin KJ, Han J, Kim DS. Significance of high-risk human papillomavirus detection by

polymerase chain reaction in primary cervical cancer screening. Cytopathology 2001;12:75–83.

39. Ostrbenk A, Xu L, Arbyn M et al. Clinical and analytical evaluation of the Anyplex II HPV HR Detection assay within the VALGENT-3 framework. Journal of Clinical Microbiology 2018;56(11). pii: e01176-18.
40. Ostrbenk Valencak A, Sterbenc A, Seme K, Poljak M. Alinity m HR HPV Assay Fulfills Criteria for Human Papillomavirus Test Requirements in Cervical Cancer Screening Settings. Journal of clinical microbiology. 2019;58(1).
41. Pankaj S, Kumari A, Kumari S, et al. Evaluation of Sensitivity and Specificity of Pap Smear, LBC and HPV in Screening of Cervical Cancer. Indian Journal of Gynecologic Oncology. 2018;16(3):49.
42. Paraskevaidis E, Malamou-Mitsi V, Koliopoulos G, Pappa L, Lolis E, Georgiou I, et al. Expanded cytological referral criteria for colposcopy incervical screening: comparison with human papillomavirus testing. Gynecol Oncol 2001;82:355–9.
43. Passamonti B, Gustinucci D, Giorgi Rossi P et al. Cervical human papilloma virus (HPV) DNA primary screening test: Results of a population-based screening programme in central Italy. Journal of Medical Screening.2017;24(3):153-162.
44. Pesic A, Krings A, Hempel M, Preyer R, Chatzistamatiou K, Agorastos T, Kaufmann AM. CIN2+ detection of the HPV DNA Array genotyping assay in comparison with the Cobas 4800 HPV test and cytology. Virol J. 2019 Jul 23;16(1):92.
45. Ronco G, Giorgi-Rossi P, Carozzi F, et al. Human papillomavirus testing and liquid-based cytology in primary screening of women younger than 35 years: results at recruitment for a randomised controlled trial. Lancet Oncol. 2006 Jul;7(7):547-55.
46. Ronco G, Giorgi-Rossi P, Carozzi F, et al. Results at recruitment from a randomized controlled trial comparing human papillomavirus testing alone with conventional cytology as the primary cervical cancer screening test. J Natl Cancer Inst. 2008 Apr 2;100(7):492-501.
47. Ronco G, Segnan N, Giorgi-Rossi P, et al. Human papillomavirus testing and liquid-based cytology: results at recruitment from the new technologies for cervical cancer randomized controlled trial. J Natl Cancer Inst. 2006 Jun 7;98(11):765-74.
48. Salmeron J, Lazcano-Ponce E, Lorincz A, Hernandez M, Hernandez P, Leyva A, et al. Comparison of HPV-based assays with Papanicolaou smears for cervical cancer screening in Morelos State, Mexico. Cancer Causes Control 2003;14:505–12.
49. Sarian LO, Derchain SF, Naud P, Roteli-Martins C, Longatto-Filho A, Tatti S, et al. Evaluation of visual inspection with acetic acid (VIA), Lugol's Iodine (VILI), cervical cytology and HPV testing as cervical screening tools in Latin America. This report refers to partial results from the LAMS study. J Med Screen 2005;12:142–9.
50. Saville M, Sultana F, Malloy MJ, et al. Clinical Validation of the cobas HPV Test on the cobas 6800 System for the Purpose of Cervical Screening. Journal of clinical microbiology. 2019;57(2).

51. Schiffman M, Herrero R, Hildesheim A, Sherman ME, Bratti M, Wacholder S, et al. HPV DNA testing in cervical cancer screening: results from women in a high-risk province of Costa Rica. JAMA 2000; 283:87–93.
52. Shipitsyna E, Zolotoverkhaya E, Kuevda D, Nasonova V, Romanyuk T, Khachaturyan A, et al. Prevalence of high-risk human papillomavirus types and cervical squamous intraepithelial lesions in women over 30 years of age in St. Petersburg, Russia. Cancer Epidemiology 2011;35:160-4.
53. Sun P, Song Y, Ruan G et al. Clinical validation of the PCR-reverse dot blot human papillomavirus genotyping test in cervical lesions from Chinese women in the Fujian province: a hospital-based population study. Journal of Gynecologic Oncology 2017;28(5):e50.
54. Syrjaenen S, Shabalova I. Human papillomavirus testing and conventional Pap smear cytology as optional screening tools of women at different risks for cervical cancer in the countries of the former Soviet Union. J Low Genit Tract Dis 2002;6:97–110.
55. Szarewski A, Cadman L, Mallett S, et al. Human papillomavirus testing by self-sampling: assessment of accuracy in an unsupervised clinical setting. J Med Screen 2007; 14: 34–42.
56. Toliman PJ, Kaldor JM, Badman SG et al. Evaluation of self-collected vaginal specimens for the detection of high-risk human papillomavirus infection and the prediction of high-grade cervical intraepithelial lesions in a high-burden, low-resource setting. Clinical Microbiology & Infection. 2019;25(4):496-503.
57. Tshomo U, Franceschi S, Tshokey T et al. Evaluation of cytology versus human papillomavirus-based cervical cancer screening algorithms in Bhutan. Oncotarget. 2017; 8(42):72438-72446.
58. Viti J, Poljak M, Ostrbenk A et al. Validation of EUROArray HPV test using the VALGENT framework. Journal of Clinical Virology 2018;108:38-42.
59. Wang HR, Liao GD, Chen W et al. [Clinical value of p16/Ki-67 immunocytochemical dual staining in cervical cancer screening]. [Chinese]. Chung-Hua Chung Liu Tsa Chih [Chinese Journal of Oncology]. 2017;39(8):636-640.
60. Wang HR, Liao GD, Jiang Y et al. [Diagnostic value of p16/mcm2 dual staining in cervical intraepithelial neoplasia and its association with high-risk HPV infection]. [Chinese]. Chung-Hua Liu Hsing Ping Hsueh Tsa Chih Chinese Journal of Epidemiology. 2017;38(9):1241-1245.
61. Wang HR, Liao GD, Jiang Y, Li YC, Qiao YL, Chen W. [Diagnostic value of p16/mcm2 dual staining in cervical intraepithelial neoplasia and its association with high-risk HPV infection]. Zhonghua Liu Xing Bing Xue Za Zhi. 2017;38(9):1241-1245.
62. Wang JJ, Dong J, Deng ZX, Wang PF, Zhang XX, Du Y. [HPV E6 and E7 mRNA combined with HPV 16 and 18 or 45 genotyping testing as a means of cervical cancer opportunistic screening]. Zhonghua fu chan ke za zhi. 2019;54(5):301-306.
63. Wang L, Jiang MY, Qin Y et al. [Application of isothermal HPV DNA amplification test in cervical cancer screening]. [Chinese]. Chung-Hua Chung Liu Tsa Chih [Chinese Journal of Oncology].

2018;40(4):313-318.
64. Wang Y, Gao S, Wang Y, Chen F, Deng H, Lu Y. The Efficiency of Type-Specific High-Risk Human Papillomavirus Models in the Triage of Women with Atypical Squamous Cells of Undetermined Significance. Cancer Manag Res. 2020 Jul 1;12:5265-5275.
65. Wikström I, Lindell M, Sanner K, Wilander E. Self-sampling and HPV testing or ordinary Pap-smear in women not regularly attending screening: a randomised study. Br J Cancer 2011; 105: 337–39.
66. Wright T, Denny L, Kuhn L, Pollack A, Lorincz A. HPV DNA testing of self-collected vaginal samples compared with cytologic screening to detect cervical cancer. JAMA 2000;283:81-6.
67. Wu R, Belinson SE, Du H, Na W, Qu X, Wu R, et al. Human papillomavirus messenger RNA assay for cervical cancer screening: the Shenzhen Cervical Cancer Screening Trial I. International Journal of Gynecological Cancer 2010;20:1411-4.
68. Zhang SK, Luo XP, Li ZF, Su Z, Xia JC, Hu GY, Zhu YJ, Xie LX, Feng XX, Sun XB, Chen W, Qiao YL. [Performance of human papillomavirus typing test in cervical precancer lesions and cervical cancer screening]. Zhonghua Zhong Liu Za Zhi. 2020 Mar 23;42(3):252-256.
69. Zhang X, Zhao G, Bi H, Zhou M, Wang X, Juan J. Exploring an Appropriate Method of Cervical Cancer Screening in Rural China. Asia Pac J Public Health. 2019 Oct;31(7):652-658.
70. Zhao FH, Lewkowitz AK, Chen F, et al. Pooled analysis of a self-sampling HPV DNA Test as a cervical cancer primary screening method. J Natl Cancer Inst 2012; 104: 178–88.
71. Zhao X, Wu Q, Wang X et al. The performance of human papillomavirus DNA detection with type 16/18 genotyping by hybrid capture in primary test of cervical cancer screening: a cross-sectional study in 10,669 Chinese women. Clin Microbiol Infect. 2018 Dec;24(12):1322-1327.
72. Zhao XL, Remila R, Hu SY et al. [Comparison of screening performance between primary high-risk HPV screening and high-risk HPV screening plus liquid-based cytology cotesting in diagnosis of cervical precancerous or cancerous lesions]. [Chinese]. Chung-Hua Yu Fang i Hsueh Tsa Chih [Chinese Journal of Preventive Medicine]. 2018; 52(5):469-474.
73. Zhao YQ, Dai Y, Dang L, et al. [Real-world research on cervical cancer screening program and effect evaluation for Chinese population]. Zhonghua zhong liu za zhi [Chinese journal of oncology]. 2018;40(10):764-771.

Ineligible population or relevant data separately unextractable (n = 55)

1. Angstetra D, Tait T, Tan J, et al. Should liquid-based cytology be performed prior to colposcopy? A comparison of the accuracy, unsatisfactory rates and cost in a tertiary referral setting. Aust N Z J Obstet Gynaecol. 2009 Dec;49(6):681-4.
2. Asciutto KC,Ernstson A,Forslund O,Borgfeldt C, et al. Self-sampling with HPV mRNA analyses from vagina and urine compared with cervical samples. Journal of Clinical Virology 2018;101:69-73.
3. Bergeron C, Jeannel D, Poveda J, Cassonnet P, Orth G. Human papillomavirus testing in women with

mild cytologic atypia. Obstet. Gynecol. 2000;95:821–827.

4. Bhatla N, Dar L, Patro AR, Kumar P, Kriplani A, Gulati A, et al. Can human papillomavirus DNA testing of self-collected vaginal samples compare with physician-collected cervical samples and cytology for cervical cancer screening in developing countries? Cancer Epidemiol. 2009;33:446–450.
5. Bottari F, Boveri S, Iacobone AD et al. Transition from Hybrid Capture 2 to Cobas 4800 in Hpv detection: sensitivity and specificity for Cin2+ in two time periods. Infectious Diseases 2018;50:7:554-559.
6. Carozzi F, Bisanzi S, Sani C, Zappa M, Cecchini S, Ciatto S, et al. Agreement between the AMPLICOR human papillomavirus test and the hybrid capture 2 assay in detection of high-risk human papillomavirus and diagnosis of biopsy-confirmed high-grade cervical disease. J. Clin. Microbiol. 2007;45:364–369.
7. Castle PE, Gutierrez EC, Leitch SV, Maus CE, McMillian RA, Nussbaumer WA, et al. Evaluation of a new DNA test for detection of carcinogenic human papillomavirus. J. Clin. Microbiol. 2011;49:3029–3032.
8. Chernesky M, Jang D, Escott N et al. Detection of cervical precancerous lesions with Aptima HPV assays using SurePath preservative fluid specimens. Papillomavirus Research. 2017; 3:155-159.
9. Clarke MA, Gradissimo A, Schiffman M et al. Human papillomavirus DNA methylation as a biomarker for cervical precancer: Consistency across 12 genotypes and potential impact on management of hpv-positive women. Clinical Cancer Research 2018;24:9:2194-2202.
10. Costa S, Sideri M, Syrjanen K, Terzano P, De Nuzzo M, De Simone P, et al. Combined Pap smear, cervicography and HPV DNA testing in the detection of cervical intraepithelial neoplasia and cancer. Acta Cytol 2000;44:310–8.
11. Cuzick J, Ambroisine L, Cadman L, Austin J, Ho L, Terry G, et al. Performance of the Abbott RealTime high-risk HPV test in women with abnormal cervical cytology smears. J. Med. Virol. 2010;82:1186–1191.
12. Dai Y, Wang L, Li D et al. Effectiveness of novel folate receptor-mediated staining solution detection (FRD) for cervical cancer screening. Medicine.2018; 97(34):e11868.
13. De Vuyst H, Claeys P, Njiru S, Muchiri L, Steyaert S, De Sutter P, et al. Comparison of pap smear, visual inspection with acetic acid, human papillomavirus DNA-PCR testing and cervicography. Int J Gynecol Obstet 2005;89(2):120–6.
14. Fontaine V, Mascaux C, Weyn C, Bernis A, Celio N, Lefèvre P, et al. Evaluation of combined general primer-mediated PCR sequencing and type-specific PCR strategies for determination of human papillomavirus genotypes in cervical cell specimens. J. Clin. Microbiol. 2007;45:928–934.
15. Fontaine V, Mascaux C, Weyn C, Bernis A, Celio N, Lefèvre P, et al. Prospective evaluation of the hybrid capture 2 and AMPLICOR human papillomavirus (HPV) tests for detection of 13 high-risk HPV genotypes in atypical squamous cells of uncertain significance. J. Clin. Microbiol. 2007;45:313–

316.

16. Garcia F, Barker B, Santos C, et al. Cross-sectional study of patient- and physician-collected cervical cytology and human papillomavirus. Obstet Gynecol. 2003; 102: 266-272.
17. Guan Y, Gravitt PE, Howard R, et al. Agreement for HPV genotyping detection between self-collected specimens on a FTA cartridge and clinician-collected specimens. J Virol Methods 2013; 189: 167–71.
18. Guo GZ, Zhao Y. [Analysis of cervical cancer screening results in women aged 50 and older in Beijing and Guizhou]. Zhonghua zhong liu za zhi [Chinese journal of oncology]. 2018;40(12):922-926.
19. He P, Gong H, Gao Y, Chen M, Lu Q. Clinical Significance of Liquid-based Cytology Combined with Human Papillomavirus DNA Detection for Cervical Lesion Screening. Anti-Tumor Pharmacy. 2019;9(3):456-460.
20. Huang SL, Chao A, Hsueh S, Chao FY, Huang CC, Yang JE, et al. Comparison between the hybrid capture II test and an SPF1/GP6+ PCR-based assay for detection of human papillomavirus DNA in cervical swab samples. J. Clin. Microbiol. 2006;44:1733–1739.
21. Jiang L, Zeng Y, Li J, et al. Performance of high-risk human papillomavirus testing in the triage of abnormal cervical cytology among Chinese younger women in Shanghai, China. Asian Pac J Cancer Prev. 2011;12(11):2963-7.
22. Jin J, Yue CY. Analysis of the efficacy of liquid-based cytology combined with HPV genotypes in screening cervical lesions in women of different ages. Journal of Laboratory Medicine 2020;44(3):151-156.
23. Klug SJ, Molijn A, Schopp B, Holz B, Iftner A, Quint W, et al. Comparison of the performance of different HPV genotyping methods for detecting genital HPV types. J. Med. Virol. 2008;80:1264–1274.
24. Kocsis A, Takacs T, Jeney C et al. Performance of a new HPV and biomarker assay in the management of hrHPV positive women: Subanalysis of the ongoing multicenter TRACE clinical trial (n>6,000) to evaluate POU4F3 methylation as a potential biomarker of cervical precancer and cancer. International Journal of Cancer.2017;140(5):1119-1133.
25. Kuhn L, Saidu R, Boa R, Tergas A, Moodley J, Persing D, Campbell S, Tsai WY, Wright TC, Denny L. Clinical evaluation of modifications to a human papillomavirus assay to optimise its utility for cervical cancer screening in low-resource settings: a diagnostic accuracy study. Lancet Glob Health. 2020 Feb;8(2):e296-e304.
26. Kulmala SM, Syrjänen S, Shabalova I, Petrovichev N, Kozachenko V, Podistov J, et al. Human papillomavirus testing with the hybrid capture 2 assay and PCR as screening tools. J. Clin. Microbiol. 2004;42:2470–2475.
27. Leinonen MK, Schee K, Jonassen CM et al. Safety and acceptability of human papillomavirus testing of self-collected specimens: A methodologic study of the impact of collection devices and HPV assays on sensitivity for cervical cancer and high-grade lesions. J Clin Virol. 2018;99-100:22-30.

28. Lin CJ, Lai HC, Wang KH, Hsiung CA, Liu HW, Ding DC, et al. Testing for methylated PCDH10 or WT1 is superior to the HPV test in detecting severe neoplasms (CIN3 or greater) in the triage of ASC-US smear results. Am. J. Obstet. Gynecol. 2011;204:21.e1–21.e7.
29. Mao X, Ruan G, Dong B, et al. Clinical validation of the Cervista((R)) high-risk human papillomavirus test in Chinese women from Fujian province: a cross-sectional study. Therapeutics and clinical risk management. 2018;14:2243-2253.
30. Moon JH, Jeong K, Kim K, Lee C, Jin MS, Ryu HS. Comparison of clinical performance of two high-throughput liquid bead microarray assays, GeneFinder and CareGENE, for cervical screening in the general population. Arch Virol. 2019 Nov;164(11):2699-2706.
31. Nakamura M, Ueda M, Iwata T, et al. A Clinical Trial to Verify the Efficiency of the LC-1000 Exfoliative Cell Analyzer as a New Method of Cervical Cancer Screening. Acta Cytologica. 2019;63(5):391-400.
32. Nonogaki S, Wakamatsu A, Longatto Filho A, Pereira SM, Utagawa ML, Ferreira Alves VA, et al. Hybrid capture II and polymerase chain reaction for identifying HPV infections in samples collected in a new collection medium: a comparison. Acta Cytol. 2004;8:514–520.
33. Pan D, Zhang CQ, Liang QL, Hong XC. An efficient method that combines the ThinPrep cytologic test with E6/E7 mRNA testing for cervical cancer screening. Cancer Manag Res. 2019;11:4773-4780.
34. Pesic A, Krings A, Hempel M, Preyer R, Kaufmann AM. Clinical performance of the HPV DNA Array genotyping assay in detection of CIN2+ lesions with BS GP5+/6+ MPG Luminex tested cervical samples. J Med Virol. 2020;92(1):113-118.
35. Prigenzi KCK, Heinke T, Salim RC et al. Dual p16 and Ki-67 Expression in Liquid-Based Cervical Cytological Samples Compared to Pap Cytology Findings, Biopsies, and HPV Testing in Cervical Cancer Screening: A Diagnostic Accuracy Study. Acta Cytologica. 62(2):104-114, 2018.
36. Pruski D, Millert-Kalinska S, Lewek A, Kedzia W. Sensitivity and specificity of HR HPV E6/E7 mRNA test in detecting cervical squamous intraepithelial lesion and cervical cancer. Ginekol Pol. 2019;90(2):66-71.
37. Ruan G, Song Y, Dong B et al. Cervical cancer screening using the Cervista high-risk human papillomavirus test: opportunistic screening of a hospital-based population in Fujian province, China. Cancer management and research.2018;10:3227-3235.
38. Sarian LO, Derchain S, Shabalova I, et al. Optional screening strategies for cervical cancer using standalone tests and their combinations among low- and medium-income populations in Latin America and Eastern Europe. J Med Screen. 2010;17(4):195-203.
39. Sasagawa T, Maehama T, Osaka Y et al. Comparison of the digene hybrid capture 2 and Roche cobas 4800 HPV tests for detection of CIN2+ in a referral population in Japan. J Med Virol. 2018 May;90(5):972-980.
40. Satake H, Inaba N, Kanno K, Mihara M, Takagi Y, Kondo N, Sagae S. Comparison Study of Self-

Sampled and Physician-Sampled Specimens for High-Risk Human Papillomavirus Test and Cytology. Acta Cytol. 2020;64(5):433-441.

41. Schiffman M, Wheeler CM, Dasgupta A, Solomon D, Castle PE. ALTS Group. A comparison of a prototype PCR assay and hybrid capture 2 for detection of carcinogenic human papillomavirus DNA in women with equivocal or mildly abnormal Papanicolaou smears. Am. J. Clin. Pathol. 2005;124:722–732.
42. Söderlund-Strand A, Rymark P, Andersson P, Dillner J, Dillner L. Comparison between the hybrid capture II test and a PCR-based human papillomavirus detection method for diagnosis and posttreatment follow-up of cervical intraepithelial neoplasia. J. Clin. Microbiol. 2005;43:3260–3266.
43. Sodhani P, Gupta S, Sharma JK, Parashari A, Halder K, Singh V, et al. Test characteristics of various screening modalities for cervical cancer: a feasibility study to develop an alternative strategy for resource-limited settings. Cytopathology 2006;17(6):348–52.
44. Stenvall H, Wikström I, Backlund I, Wilander E. Accuracy of HPV testing of vaginal smear obtained with a novel self-sampling device. Acta Obstet. Gynecol. Scand. 2007;86:16–21.
45. Stenvall H, Wikström I, Backlund I, Wilander E. Comparison of the AMPLICOR human papillomavirus test and the hybrid capture 2 assay for detection of high-risk human papillomavirus in women with abnormal PAP smear. J. Virol. Methods. 2008;147:10–17.
46. Stevens MP, Garland SM, Rudland E, Tan J, Quinn MA, Tabrizi SN. Comparison of the Digene hybrid capture 2 assay and Roche AMPLICOR and LINEAR ARRAY human papillomavirus (HPV) tests in detecting high-risk HPV genotypes in specimens from women with previous abnormal pap smear results. J. Clin. Microbiol. 2007;45:2130–2137.
47. Szarewski A, Ambroisine L, Cadman L, Austin J, Ho L, Terry G, et al. Comparison of predictors for high-grade cervical intraepithelial neoplasia in women with abnormal smears. Cancer Epidemiol. Biomarkers Prev. 2008;17:3033–3042.
48. Thrall MJ, Pambuccian SE, Stelow EB, et al. Impact of the more restrictive definition of atypical squamous cells introduced by the 2001 Bethesda System on the sensitivity and specificity of the Papanicolaou test: a 5-year follow-up study of Papanicolaou tests originally interpreted as ASCUS, reclassified according to Bethesda 2001 criteria. Cancer. 2008 25;114(3):171-9.
49. Torres-Ibarra L, Cuzick J, Lorincz AT, et al. Comparison of HPV-16 and HPV-18 Genotyping and Cytological Testing as Triage Testing Within Human Papillomavirus-Based Screening in Mexico. JAMA network open. 2019;2(11):e1915781.
50. Turanova OV, Belokrinitskaya TE. Diagnostic value of conventional cytology and HR-HPV testing in the detection of high grade cervical intraepithelial neoplasia. Akusherstvo i Ginekologiya (Russian Federation) 2020;1(Supplement):93-97.
51. Weynand B, Delvenne P, Polet R, Guiot Y, Arafa M, Somja J, et al. Validation of ThermoFisher's papspin for human papillomavirus detection in cervicovaginal specimens using PCR with GP5+/GP6+

primers and the hybrid capture II assay. Clin. Microbiol. Infect. 2010;16:671–675.

52. Wong OG, Lo CK, Szeto E, Cheung AN. Efficacy of Abbott RealTime high risk HPV test in evaluation of atypical squamous cells of undetermined significance from an Asian screening population. J. Clin. Virol. 2011;51:136–138.
53. Yu L, Jiang M, Qu P et al. Clinical evaluation of human papillomavirus 16/18 oncoprotein test for cervical cancer screening and HPV positive women triage. Int J Cancer. 2018 15;143(4):813-822.
54. Zhang JJ, Cao XC, Zheng XY et al. Feasibility study of a human papillomavirus E6 and E7 oncoprotein test for the diagnosis of cervical precancer and cancer. J Int Med Res. 2018;46(3):1033-1042.
55. Zhang R, Ge X, You K et al. p16/Ki67 dual staining improves the detection specificity of high-grade cervical lesions. J Obstet Gynaecol Res. 2018;44(11):2077-2084.

Cytology or pathology archive-based sampling (n = 17)

1. Barodawala SM, Chadha K, Kavishwar V, Murthy A, Shetye S. Cervical cancer screening by molecular Pap-transformation of gynecologic cytology. Diagnostic cytopathology. 2019;47(5):374-381.
2. Bhatia R, Serrano I, Wennington H et al. Evaluation of a novel single-tube method for extended genotyping of human papillomavirus. J Clin Microbiol. 2018;56(3). pii: e01687-17.
3. Bottari F, Boveri S, Iacobone AD et al. Transition from Hybrid Capture 2 to Cobas 4800 in Hpv detection: sensitivity and specificity for Cin2+ in two time periods. Infect Dis 2018;50(7):554-559.
4. Fokom Domgue J, Schiffman M, Wentzensen NH et al. Assessment of a New Lower-Cost Real-Time PCR Assay for Detection of High-Risk Human Papillomavirus: Useful for Cervical Screening in Limited-Resource Settings?. J Clin Microbiol 2017;55(8):2348-2355.
5. Fuller MY, Mody RR, Luna E, et al. Performance of Roche cobas high-risk human papillomavirus (hrHPV) testing in the two most common liquid-based Papanicolaou test platforms. Journal of the American Society of Cytopathology. 2018;7(3):142-148.
6. Ge Y, Christensen P, Luna E, et al. Role of HPV genotyping in risk assessment among cytology diagnosis categories: analysis of 4562 cases with cytology-HPV cotesting and follow-up biopsies. Int J Gynecol Cancer. 2019. pii: ijgc-2018-000024.
7. Han M, Li J, Austin M, Varma KR, Zhang H, Zhao C. Human Papillomavirus (HPV) 16 and 18/45 Genotyping-Directed Follow-up of Women With Messenger RNA HPV-Positive, Cytology-Negative Cervical Screening Test Results. American journal of clinical pathology. 2020;153(2):243-250.
8. Karbalaie Niya MH, Mobini Kesheh M, Keshtmand G, et al. Integration rates of human papilloma virus genome in a molecular survey on cervical specimens among Iranian patients. European journal of cancer prevention : the official journal of the European Cancer Prevention Organisation (ECP). 2019;28(6):537-543.

9. Kir G, Seneldir H, Cosan Sarbay B et al. The clinical performance of computer-assisted liquid-based cytology, primary hrHPV screening, and cotesting at a Turkish Tertiary Care Hospital. Diagnostic Cytopathology.2018; 46(1):3-8.
10. McDermott JE, Symanowski JT, Livasy CA, Greger G, Longshore JW. A retrospective analysis of the clinical performance of human papillomavirus testing using SurePath sample collection. Journal of the American Society of Cytopathology. 2014;3(3):165-169.
11. Polman NJ, Ostrbenk A, Xu L et al. Evaluation of the Clinical Performance of the HPV-Risk Assay Using the VALGENT-3 Panel. J Clin Microbiol. 2017;55(12):3544-3551.
12. Wang HY, Kim H, Park KH. Diagnostic performance of the E6/E7 mRNA-based Optimygene HR-HPV RT-qDx assay for cervical cancer screening. Int J Infect Dis. 2019 Jan;78:22-30.
13. Wong OG, Ho MW, Tsun OK et al. An automated quantitative DNA image cytometry system detects abnormal cells in cervical cytology with high sensitivity. Cytopathology. 2018;29(3):267-274.
14. Wong OGW, Ng IFY, Tsun OKL, Pang HH, Ip PPC, Cheung ANY. Machine Learning Interpretation of Extended Human Papillomavirus Genotyping by Onclarity in an Asian Cervical Cancer Screening Population. Journal of clinical microbiology. 2019;57(12).
15. Wong OGW, Tsun OKL, Tsui EY, Chow JNK, Ip PPC, Cheung ANY. HPV genotyping and E6/E7 transcript assays for cervical lesion detection in an Asian screening population-Cobas and Aptima HPV tests. Journal of clinical virology : the official publication of the Pan American Society for Clinical Virology. 2018;109:13-18.
16. Xu L, Otrbenk A, Poljak M et al. Assessment of the Roche Linear Array HPV Genotyping Test within the VALGENT framework. J Clin Virol. 2018;98:37-42.
17. Yoon YA, Kim BH, Heo SH et al. Comparative evaluation of the Omniplex-HPV and RFMP HPV PapilloTyper for detecting human papillomavirus genotypes in cervical specimens. Arch Virol. 2018;163(4):969-976.

Longitudinal data (n = 9)

1. Castle PE, Lorincz AT, Scott DR, Sherman ME, Glass AG, Rush BB, et al. Comparison between prototype hybrid capture 3 and hybrid capture 2 human papillomavirus DNA assays for detection of high-grade cervical intraepithelial neoplasia and cancer. J. Clin. Microbiol. 2003;41:4022–4030.
2. Cuzick J, Szarewski A, Mesher D, Cadman L, Austin J, Perryman K, et al. Long-term follow-up of cervical abnormalities among women screened by HPV testing and cytology-results from the Hammersmith study. Int. J. Cancer. 2008;122:2294–2300.
3. Depuydt CE, Makar AP, Ruymbeke MJ, et al. BD-ProExC as adjunct molecular marker for improved detection of CIN2+ after HPV primary screening. Cancer Epidemiol Biomarkers Prev. 2011;20(4):628-37.
4. Depuydt CE, Benoy IH, Beert JF, et al. Clinical validation of a type-specific real-time quantitative

human papillomavirus PCR against the performance of hybrid capture 2 for the purpose of cervical cancer screening. J Clin Microbiol. 2012;50(12):4073-7.
5. Dong B, Chen L, Lin W, Su Y, Mao X, Pan D, Ruan G, Xue H, Kang Y, Sun P. Cost-effectiveness and accuracy of cervical cancer screening with a high-risk HPV genotyping assay vs a nongenotyping assay in China: an observational cohort study. Cancer Cell Int. 2020 Aug 28;20:421.
6. Horn J, Denecke A, Luyten A, Rothe B, Reinecke-Lüthge A, Mikolajczyk R, Petry KU. Reduction of cervical cancer incidence within a primary HPV screening pilot project (WOLPHSCREEN) in Wolfsburg, Germany. Br J Cancer. 2019 May;120(10):1015-1022.
7. Kang Y, Sun P, Mao X, Dong B, Ruan G, Chen L. PCR-reverse dot blot human papillomavirus genotyping as a primary screening test for cervical cancer in a hospital-based cohort. J Gynecol Oncol. 2019 May;30(3):e29. doi: 10.3802/jgo.2019.30.e29.
8. Tracht J, Wrenn A, Eltoum IE et al. Primary HPV testing verification: A retrospective ad-hoc analysis of screening algorithms on women doubly tested for cytology and HPV. Diagn Cytopathol. 2017;45(7):580-586.
9. Zhang L, Xu XQ, Hu SY et al. Durability of clinical performance afforded by self-collected HPV testing: A 15-year cohort study in China. Gynecol Oncol. 2018;151(2):221-228.

Accuracy data unextractable (n = 8)

1. Bulkmans NW, Rozendaal L, Snijders PJ, et al. POBASCAM, a population-based randomised controlled trial for implementation of high-risk HPV testing in cervical screening. Int J Cancer 2004; 110: 94–110.
2. Jones HE, Mansukhani MM, Tong GX, et al. Validity and reliability of using a self-lavaging device for cytology and HPV testing for cervical cancer screening: findings from a pilot study. PLoS One. 2013 Dec 20;8(12):e82115.
3. Lorenzato FR, Singer A, Ho L, et al. Human papillomavirus detection for cervical cancer prevention with polymerase chain reaction in self-collected samples. Am J Obstet Gynecol. 2002;186(5):962-8.
4. Nonogaki S, Wakamatsu A, Filho AL, Roteli-Martins C, Maeda C, di Loreto MY, et al. Molecular strategies for identifying human papillomavirus infection in routinely processed samples: focus on paraffin sections. J. Low. Genit. Tract Dis. 2005;9:219–224.
5. Naucler P, Ryd W, Törnberg S, et al. Human papillomavirus and Papanicolaou tests to screen for cervical cancer. N Engl J Med. 2007 Oct 18;357(16):1589-97.
6. Sankaranarayanan R, Nene BM, Dinshaw KA, Mahe C, Jayant K, Shastri S, et al. A cluster randomized controlled trial of visual, cytological and human papillomavirus screening for cancer of the cervix in rural India. Int J Cancer 2005;116:617–23.
7. Venturoli S, Cricca M, Bonvicini F, Giosa F, Pulvirenti FR, Galli C, et al. Human papillomavirus DNA testing by PCR-ELISA and hybrid capture II from a single cytological specimen: concordance

and correlation with cytological results. J. Clin. Virol. 2002;25:177–185.

8. Yarkin F, Chauvin S, Konomi N, Wang W, Mo R, Bauchman G, et al. Detection of HPV DNA in cervical specimens collected in cytologic solution by ligation-dependent PCR. Acta Cytol. 2003;47:450–456.

Duplicate publication (n = 2)

1. Pan Q, Li L, Qiao Y. Comparative study on liquid-based cytology for cervical carcinoma screening in a high-risk area of China [in Chinese]. Zhonghua Zhong Liu Za Zhi. 2001; 23: 309-312.
2. Wu LY, Li N, Zhang WH, et al. Value of acetic acid smear test for cervical cancer screening [in Chinese]. Ai Zheng. 2003; 22: 1096-1098.

1st generation hybrid capture (n = 1)

1. Ratnam S, Franco EL, Ferenczy A. Human papillomavirus testing for primary screening of cervical cancer precursors. Cancer Epidemiol Biomarkers Prev. 2000;9(9):945-51.

Protocol (n = 1)

1. Morisada T, Saika K, Saito E et al. Population-based cohort study assessing the efficacy of cervical cytology (Pap smear) and human papillomavirus (HPV) testing as modalities for cervical cancer screening. Jpn J Clin Oncol. 2018;48(5):495-498.

Irrelevant (n = 56)

1. Aitken CA, van Agt HME, Siebers AG, van Kemenade FJ, Niesters HGM, Melchers WJG, Vedder JEM, Schuurman R, van den Brule AJC, van der Linden HC, Hinrichs JWJ, Molijn A, Hoogduin KJ, van Hemel BM, de Kok IMCM. Introduction of primary screening using high-risk HPV DNA detection in the Dutch cervical cancer screening programme: a population-based cohort study. BMC Med. 2019 Dec 11;17(1):228.
2. Aoyama-Kikawa S, Fujita H, Hanley SJB et al. Comparison of human papillomavirus genotyping and cytology triage, COMPACT Study: Design, methods and baseline results in 14 642 women. Cancer Sci 2018;109(6):2003-2012.
3. Arbyn M, Peeters E, Benoy I et al. VALHUDES: A protocol for validation of human papillomavirus assays and collection devices for HPV testing on self-samples and urine samples. J Clin Virol 2018;107:52-56.
4. Belinson JL, Pan QJ, Biscotti C, et al. Primary screening with liquid-based cytology in an unscreened population in rural China, with an emphasis on reprocessing unsatisfactory samples. Acta Cytol. 2002; 46: 470-474.
5. Belinson JL, Pretorius RG, Zhang WH, et al. Cervical cancer screening by simple visual inspection

after acetic acid. Obstet Gynecol. 2001; 98: 441-444.
6. Benoy I, Xu L, Vanden Broeck D, et al. Using the VALGENT-3 framework to assess the clinical and analytical performance of the RIATOL qPCR HPV genotyping assay. Journal of clinical virology : the official publication of the Pan American Society for Clinical Virology. 2019;120:57-62.
7. Bergengren L, Lillsunde-Larsson G, Helenius G, Karlsson MG. HPV-based screening for cervical cancer among women 55-59 years of age. PLoS One. 2019 Jun 14;14(6):e0217108.
8. Dhakal R, Makaju R, Sharma S, Bhandari S, Shrestha S, Bastakoti R. Correlation of Cervical Pap Smear with Biopsy in the Lesion of Cervix. Kathmandu University medical journal (KUMJ). 2016;14(55):254-257.
9. Ferrera A, Valladares W, Cabrera Y, et al. Performance of an HPV 16/18 E6 oncoprotein test for detection of cervical precancer and cancer. International journal of cancer. 2019;145(8):2042-2050.
10. Gultekin M, Zayifoglu Karaca M, Kucukyildiz I et al. Initial results of population based cervical cancer screening program using HPV testing in one million Turkish women. Int J Cancer 2018;142:9:1952-1958.
11. Guney G, Arslan E, Bas Y, Turgal E. Two-Year Results of Community-Based Screening Program for Human Papilloma Virus DNA in Corum Province. Turk patoloji dergisi. 2019;35(2):102-106.
12. Gustavsson I, Aarnio R, Myrnas M, et al. Clinical validation of the HPVIR high-risk HPV test on cervical samples according to the international guidelines for human papillomavirus DNA test requirements for cervical cancer screening. Virology journal. 2019;16(1):107.
13. Han L, Chang X, Song P, Gao L, Zhang Y, An L, Shen J. An on-going study of three different cervical cancer screening strategies based on primary healthcare facilities in Beijing China. J Infect Public Health. 2020 Apr;13(4):577-583.
14. Hashiguchi M, Nakao Y, Honda A, et al. What Has Changed Since the Introduction of Human Papillomavirus Testing with the Cytology-Based Cervical Cancer Screening System in Japan A Social Experiment. Acta cytologica. 2019;63(5):385-390.
15. Heideman DAM, Xu L, Hesselink AT, et al. Clinical performance of the HPV-Risk assay on cervical samples in SurePath medium using the VALGENT-4 panel. Journal of clinical virology : the official publication of the Pan American Society for Clinical Virology. 2019;121:104201.
16. Hooi DJ, Lissenberg-Witte BI, Kenter G, et al. Human papillomavirus (HPV) prevalence and associated risk factors in women from Curacao. PloS one. 2018;13(7):e0199624.
17. Jeon J-S, Kim JW, Kim JK et al. Prevalence of human papillomavirus infection and genotype distribution determined via real-time PCR in a Korean medical check-up population. Microbiology and Biotechnology Letters 2018;46:2:171-179.
18. Kamineni A, Tiro JA, Beaber EF, et al. Cervical cancer screening research in the PROSPR I consortium: Rationale, methods and baseline findings from a US cohort. International journal of cancer. 2019;144(6):1460-1473.

19. Kang Y, Sun P, Mao X, Dong B, Ruan G, Chen L. PCR-reverse dot blot human papillomavirus genotyping as a primary screening test for cervical cancer in a hospital-based cohort. Journal of gynecologic oncology. 2019;30(3):e29.
20. Kares S, Veijalainen O, Kholová I, Tirkkonen M, Vuento R, Huhtala H, Tuimala V, Mäenpää J, Kujala P. HIGH-RISK HPV testing as the primary screening method in an organized regional screening program for cervical cancer: the value of HPV16 and HPV18 genotyping? APMIS. 2019 Nov;127(11):710-716.
21. Katanga J, Kjaer SK, Manongi R, Wu CS, Iftner T, Waldstrom M, Pembe AB, Mwaiselage J, Rasch V. Performance of careHPV, hybrid capture 2 and visual inspection with acetic acid for detection of high-grade cervical lesion in Tanzania: A cross-sectional study. PLoS One. 2019 Jun 19;14(6):e0218559.
22. Kurokawa T, Yoshida Y, Iwanari O, et al. Implementation of primary HPV testing in Japan. Mol Clin Oncol. 2020;13(4):22.
23. Landy R, Schiffman M, Sasieni PD, Cheung LC, Katki HA, Rydzak G, Wentzensen N, Poitras NE, Lorey T, Kinney WK, Castle PE. Absolute risks of cervical precancer among women who fulfill exiting guidelines based on HPV and cytology cotesting. Int J Cancer. 2020 Feb 1;146(3):617-626.
24. Leinonen MK, Schee K, Jonassen CM et al. Safety and acceptability of human papillomavirus testing of self-collected specimens: A methodologic study of the impact of collection devices and HPV assays on sensitivity for cervical cancer and high-grade lesions. J Clin Virol 2018;99-100:22-30.
25. Li L, Zheng Z, Li L. Evaluation of human-papillomavirus screening for cervical cancer in China's rural population. PeerJ. 2019 Dec 20;7:e8152.
26. Lindroth Y, Borgfeldt C, Thorn G, Bodelsson G, Forslund O. Population-based primary HPV mRNA cervical screening compared with cytology screening. Preventive medicine. 2019;124:61-66.
27. Ma Y, Di J, Bi H, Zhao Q, Qin T, Xu W, Liu Z, Yi N, Zhao J, Zhou D, Chen J, Yang Q. Comparison of the detection rate of cervical lesion with TruScreen, LBC test and HPV test: A Real-world study based on population screening of cervical cancer in rural areas of China. PLoS One. 2020 Jul 7;15(7):e0233986.
28. Machalek DA, Roberts JM, Garland SM, et al. Routine cervical screening by primary HPV testing: early findings in the renewed National Cervical Screening Program. The Medical journal of Australia. 2019;211(3):113-119.
29. Mo LZ, Monnier-Benoit S, Kantelip B, Petitjean A, Riethmuller D, Prétet JL, et al. Comparison of AMPLICOR and hybrid capture II assays for high risk HPV detection in normal and abnormal liquid-based cytology: use of INNO-LiPA genotyping assay to screen the discordant results. J. Clin. Virol. 2008;41:104–110.
30. Morisada T, Teramoto K, Takano H et al. CITRUS, cervical cancer screening trial by randomization of HPV testing intervention for upcoming screening: Design, methods and baseline data of 18,471

women. Cancer Epidemiol 2017;50(Pt A):60-67.

31. Murillo R, Luna J, Gamboa O, et al. Cervical cancer screening with naked-eye visual inspection in Colombia. Int J Gynaecol Obstet. 2010;109(3):230-4.
32. Muwonge R, Manuel Mda G, et al. Visual screening for early detection of cervical neoplasia in Angola. Int J Gynaecol Obstet. 2010;111(1):68-72.
33. Newman H, Hu J, Li X, et al. Evaluation of portable colposcopy and human papillomavirus testing for screening of cervical cancer in rural China. International journal of gynecological cancer : official journal of the International Gynecological Cancer Society. 2019;29(1):23-27.
34. Ngelangel CA, Limson GM, Cordero CP, et al. Acetic-acid guided visual inspection vs. cytology-based screening for cervical cancer in the Philippines. Int J Gynaecol Obstet. 2003;83(2):141-50.
35. Ngoma T, Muwonge R, Mwaiselage J, et al. Evaluation of cervical visual inspection screening in Dar es Salaam, Tanzania. Int J Gynaecol Obstet. 2010;109(2):100-4.
36. Rebolj M, Rimmer J, Denton K, et al. Primary cervical screening with high risk human papillomavirus testing: observational study. BMJ (Clinical research ed). 2019;364:l240.
37. Riethmuller D, Gay C, Bertrand X, Bettinger D, Schaal JP, Carbillet JP, et al. Genital human papillomavirus infection among women recruited for routine cervical cancer screening or for colposcopy determined by hybrid capture II and polymerase chain reaction. Diagn. Mol. Pathol. 1999;8:157–164.
38. Ruan G, Song Y, Dong B, et al. Cervical cancer screening using the Cervista high-risk human papillomavirus test: opportunistic screening of a hospital-based population in Fujian province, China. Cancer management and research. 2018;10:3227-3235.
39. Sahlgren H, Elfstrom KM, Lamin H, et al. Colposcopic and histopathologic evaluation of women with HPV persistence exiting an organized screening program. American journal of obstetrics and gynecology. 2020;222(3):253.e251-253.e258.
40. Sankaranarayanan R, Basu P, Wesley RS, et al. Accuracy of visual screening for cervical neoplasia: Results from an IARC multicentre study in India and Africa. Int J Cancer. 2004;20;110(6):907-13.
41. Sankaranarayanan R, Shastri SS, Basu P, et al. The role of low-level magnification in visual inspection with acetic acid for the early detection of cervical neoplasia. Cancer Detect Prev. 2004;28(5):345-51.
42. Sankaranarayanan R, Thara S, Sharma A, Roy C, Shastri S, Mahe C, et al. Accuracy of conventional cytology: results from a multicentre screening study in India. J Med Screen 2004;11:77–84.
43. Sankaranarayanan R, Wesley R, Thara S, et al. Test characteristics of visual inspection with 4% acetic acid (VIA) and Lugol's iodine (VILI) in cervical cancer screening in Kerala, India. Int J Cancer. 2003;106(3):404-8.
44. Schiffman M, Kinney WK, Cheung LC et al. Relative Performance of HPV and Cytology Components of Cotesting in Cervical Screening. J Natl Cancer Inst 2018;110(5):501-508.
45. Sherman ME, Lorincz AT, Scott DR,Wacholder S, Castle PE, Glass AG, et al. Baseline cytology,

human papillomavirus testing, and risk for cervical neoplasia: a 10-year cohort analysis. J Natl Cancer Inst 2003;95:46–52.

46. Takamatsu R, Nabandith V, Pholsena V et al. Cervical cytology and human papillomavirus among asymptomatic healthy volunteers in Vientiane, Lao PDR. BMC Cancer. 17(1):872, 2017 Dec 19.
47. Toliman PJ, Kaldor JM, Badman SG, et al. Performance of clinical screening algorithms comprising point-of-care HPV-DNA testing using self-collected vaginal specimens, and visual inspection of the cervix with acetic acid, for the detection of underlying high-grade squamous intraepithelial lesions in Papua New Guinea. Papillomavirus research (Amsterdam, Netherlands). 2018;6:70-76.
48. Turner SA, Deharvengt SJ, Lyons KD et al. Implementation of Multicolor Melt Curve Analysis for High-Risk Human Papilloma Virus Detection in Low- and Middle-Income Countries: A Pilot Study for Expanded Cervical Cancer Screening in Honduras. Journal of Global Oncology. (4):1-8, 2018 Sep
49. Ueda Y, Kawana K, Yanaihara N, et al. Development and evaluation of a cervical cancer screening system in Cambodia: A collaborative project of the Cambodian Society of Gynecology and Obstetrics and Japan Society of Obstetrics and Gynecology. The journal of obstetrics and gynaecology research. 2019;45(7):1260-1267.
50. University of Zimbabwe/JHPIEGO Cervical Cancer Project. Visual inspection with acetic acid for cervical-cancer screening: test qualities in a primary-care setting. University of Zimbabwe/JHPIEGO Cervical Cancer Project. Lancet. 1999 Mar 13;353(9156):869-73.
51. Veijalainen O, Kares S, Kujala P, et al. Implementation of HPV-based cervical cancer screening in an organised regional screening programme: 3 years of experience. Cytopathology : official journal of the British Society for Clinical Cytology. 2019;30(2):150-156.
52. Wang MZ, Feng RM, Wang S, Duan XZ, Li D, Zhang X, Mu R, Qiao Y, Smith JS. Clinical Performance of Human Papillomavirus Testing and Visual Inspection With Acetic Acid in Primary, Combination, and Sequential Cervical Cancer Screening in China. Sex Transm Dis. 2019 Aug;46(8):540-547.
53. Westre B, Giske A, Guttormsen H, Wergeland Sorbye S, Skjeldestad FE. Quality control of cervical cytology using a 3-type HPV mRNA test increases screening program sensitivity of cervical intraepithelial neoplasia grade 2+ in young Norwegian women-A cohort study. PloS one. 2019;14(11):e0221546.
54. Wu P, Xiong H, Yang M, et al. Co-infections of HPV16/18 with other high-risk HPV types and the risk of cervical carcinogenesis: A large population-based study. Gynecologic oncology. 2019;155(3):436-443.
55. Xue P, Gao LL, Yin J, et al. A direct comparison of four high-risk human papillomavirus tests versus the cobas test: Detecting CIN2+ in low-resource settings. Journal of medical virology. 2019;91(7):1342-1350.
56. Zhang X, Zhao G, Bi H, Zhou M, Wang X, Juan J. Exploring an Appropriate Method of Cervical

Cancer Screening in Rural China. Asia-Pacific journal of public health. 2019;31(7):652-658.
